# Finite element modelling of surface-bound quinone proton coupled electron transfer voltammetry

**DOI:** 10.1039/d6cp01907b

**Published:** 2026-08-03

**Authors:** Nafiz B. Biswas, Katherine J. Levey, Tania L. Read, Julie V. Macpherson

**Affiliations:** a Department of Chemistry, University of Warwick Coventry CV4 7AL UK katherine.j.levey@warwick.ac.uk j.macpherson@warwick.ac.uk; b Centre for Diamond Science and Technology, University of Warwick Coventry CV4 7AL UK; c Leiden Institute of Chemistry, Leiden University, Einsteinweg 55 2333 CC Leiden The Netherlands

## Abstract

A numerical (finite element) model has been developed to simulate the proton coupled electron transfer (PCET) reaction for surface bound quinone species. It is easily adaptable to a wide range of PCET systems. Employing a user-friendly interface we show how factors such as scan rate, pH, buffer capacity, quinone p*K*_a_ and surface coverage, along with protonation and electron transfer rate constant(s) can impact simulated voltammograms. It is further demonstrated how the model can be used to assess the validity of Laviron plots when applied to PCET reactions for the determination of apparent electron transfer (ET) rate constants. The extracted ET rate constants from simulated voltammograms of peak potential *versus* log scan rate are compared against those obtained from analytical expressions derived for use with PCET reactions. For the (1-electron, 1-proton) 1e^−^1H^+^ system, when a fast rate of proton transfer is employed, typical of aqueous systems, there is very good agreement between the ET rate constants extracted using the two methods. In contrast, for the case of 2e^−^2H^+^ PCET, good agreement is not seen and highlights for the more complex system that even when the rate of protonation is high, slower rates of deprotonation can result (and *vice versa*), moving the system away from being ET rate-limited. Hence Laviron analysis should not be applied quantitatively to 2e^−^2H^+^ PCET systems. The simulation model is also employed to offer practical solutions for use of quinone functionalised electrodes in the voltammetric sensing of solution pH. Experimentally, deviations from 2e^−^2H^+^ PCET Nernstian behaviour are typically seen in unbuffered solutions with monolayer coverage electrodes due to local depletion (or accumulation) of protons during voltammetry. By simulating the interfacial proton concentrations at the electrode surface as a function of quinone surface coverage, it is possible to determine the required reduction in surface coverage (*ca.* two orders of magnitude) to negate such effects.

## Abstract 2

A numerical model has been developed to simulate the voltammetric response of surface-bound proton coupled electron transfer reactions, enabling insights into Laviron plot applicability and optimal surface coverage for quinone pH sensors.

## Graphical abstract

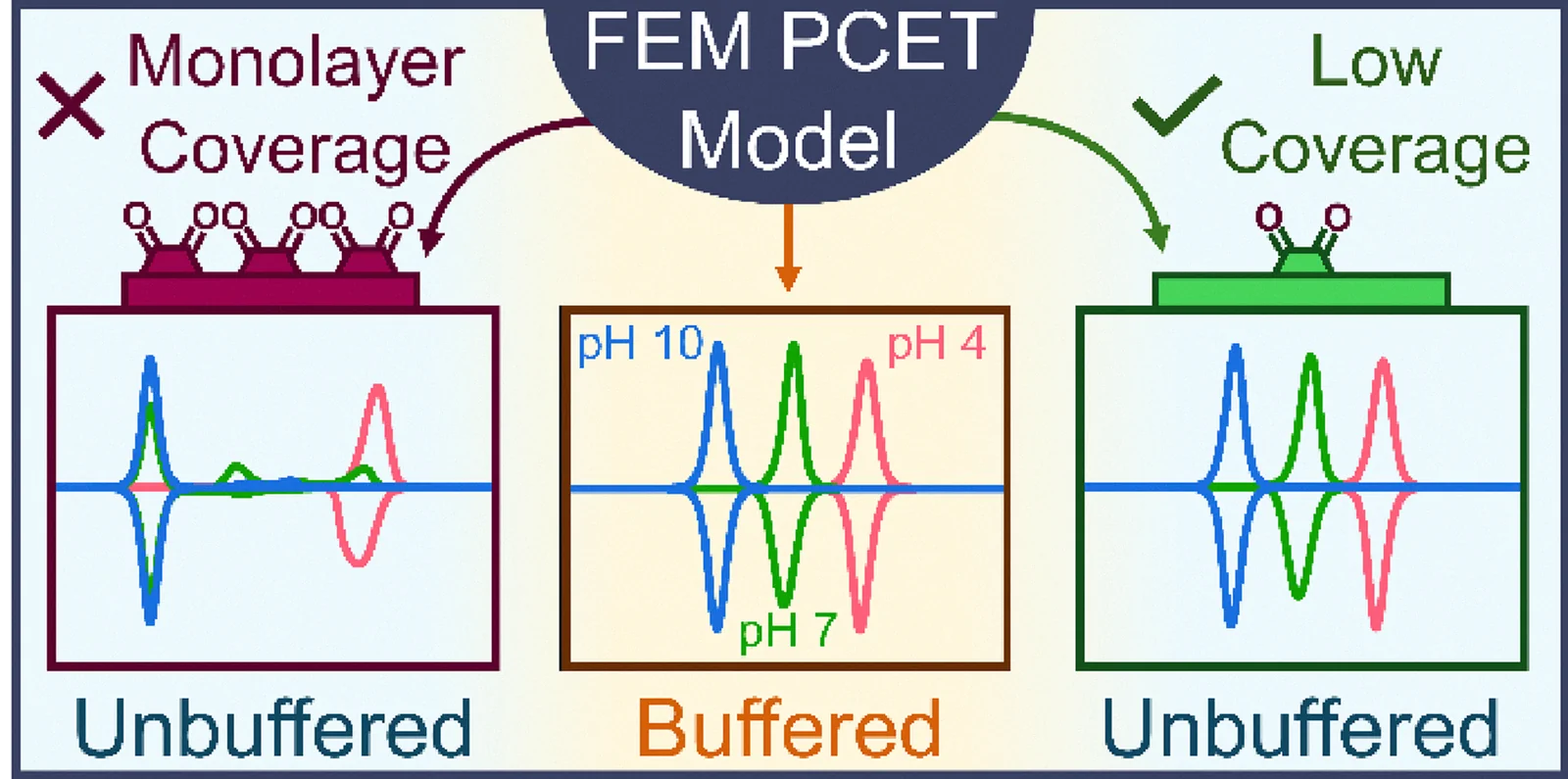

## 1. Introduction

Proton coupled electron transfer (PCET) reactions encompass a large range of processes found in both chemical and biological systems,^1–4^ where one or more proton (H^+^) and electron (e^−^) reactions occur simultaneously, either in a single (concerted) or multi (stepwise) step reaction. The mechanism depends on the reaction thermodynamics and is governed by the redox potentials (*E*_p_) and acid dissociation constants (*K*_a,*n*_) of the molecules undergoing PCET.^5^ PCET can occur homogeneously in solution or heterogeneously across the electrode/electrolyte interface.^4^ For electrode driven reactions, applications are wide ranging, including energy conversion and storage,^6–8^ catalysis and synthesis,^9,10^ electronics,^11^ environmental/medical applications^12,13^ and pH sensing.^14,15^ Many of these applications involve surface-bound molecules and to understand and optimise these PCET processes, robust models are required.

The PCET mechanism was first modelled by Jacq,^16^ who created the “Scheme of Squares”. Building on this model, Laviron laid the groundwork for detailing how voltammetric methods could be used for characterising the thermodynamics, kinetics, and mechanisms of surface-bound ET and PCET reactions.^17,18^ Quinones, which undergo 2e^−^2H^+^ transfer, were considered by Laviron to be an important class of PCET reaction.^17,19^ Laviron introduced mathematical expressions to relate the shift in voltammetric peak position, as a function of pH, to the p*K*_a,*n*_ and the standard potential (*E*^0^_*n*_) of the different steps in the PCET process. The proposed analytical models by Laviron relied on the assumption that the proton concentration at the electrode/electrolyte interface and in bulk solution were the same. Whilst this is appropriate for buffered solutions, for unbuffered solutions the situation is more complex due to PCET removing/accumulating protons locally at the electrode/electrolyte interface during voltammetry.

Building on Laviron's work, Compton and co-workers numerically evaluated the effect of solution pH, ET rate constant, mass transport, molecule surface coverage and buffer capacity on the PCET voltammetric response.^20^ From this work, the relationship between proton mass transport and surface coverage was introduced, and various mechanistic pathways were explored. However, dimensionless parameters were employed making it more challenging to easily draw meaningful comparisons with the experimental data. The models also assumed the rate of proton transfer to be infinitely fast,^20^ which is not always the case, especially when moving to non-aqueous,^21^ solid-state, or viscous electrolytes.^22^

In this paper, a model is presented which outputs the voltammetric response of molecules that undergo surface bound PCET in response to an applied electrochemical potential. The focus is on quinones given their wide applicability, but the developed models can be easily adapted to other molecules and solution phase PCET systems.^4^ The model uses a commercially available finite element method (FEM) package that is relatively simple to implement and provides a user-friendly interface. In this way, the user can understand how factors appropriate to the experimental conditions and specific quinone species of interest, such as solution pH, scan rate, quinone p*K*_a_ and surface coverage, impact the simulated voltammogram. The user can input any value for ET and protonation rates. In doing so this allows direct comparisons to be made with experimental results, enabling information on p*K*_a_ values and/or ET rate constants to be extracted, if not previously known.

The importance of such a model in combination with a user-friendly interface is further illustrated with simulations which explore the voltammetric response of surface bound quinone molecules in weakly or unbuffered solutions. Such systems are employed for voltammetric pH sensing,^23–29^ where the voltammetric peak position shifts in a Nernstian manner with changing solution pH. Whilst exemplary performance is observed in buffered solutions, the response deviates from theory in unbuffered solutions, especially in the pH range 6–8, due to perturbation of local proton concentrations during voltammetry.^29^ Reducing surface coverage below monolayer was postulated as a mitigation strategy based on experimental data.^29^ This paper shows how FEM simulations can be used to provide theoretical support for experimental predictions and provide quantitative information on the required surface coverages to enable successful operation in unbuffered solutions.

## 2. Experimental/methods section (model details)

### 2.1. Model introduction

**Fig. 1:**
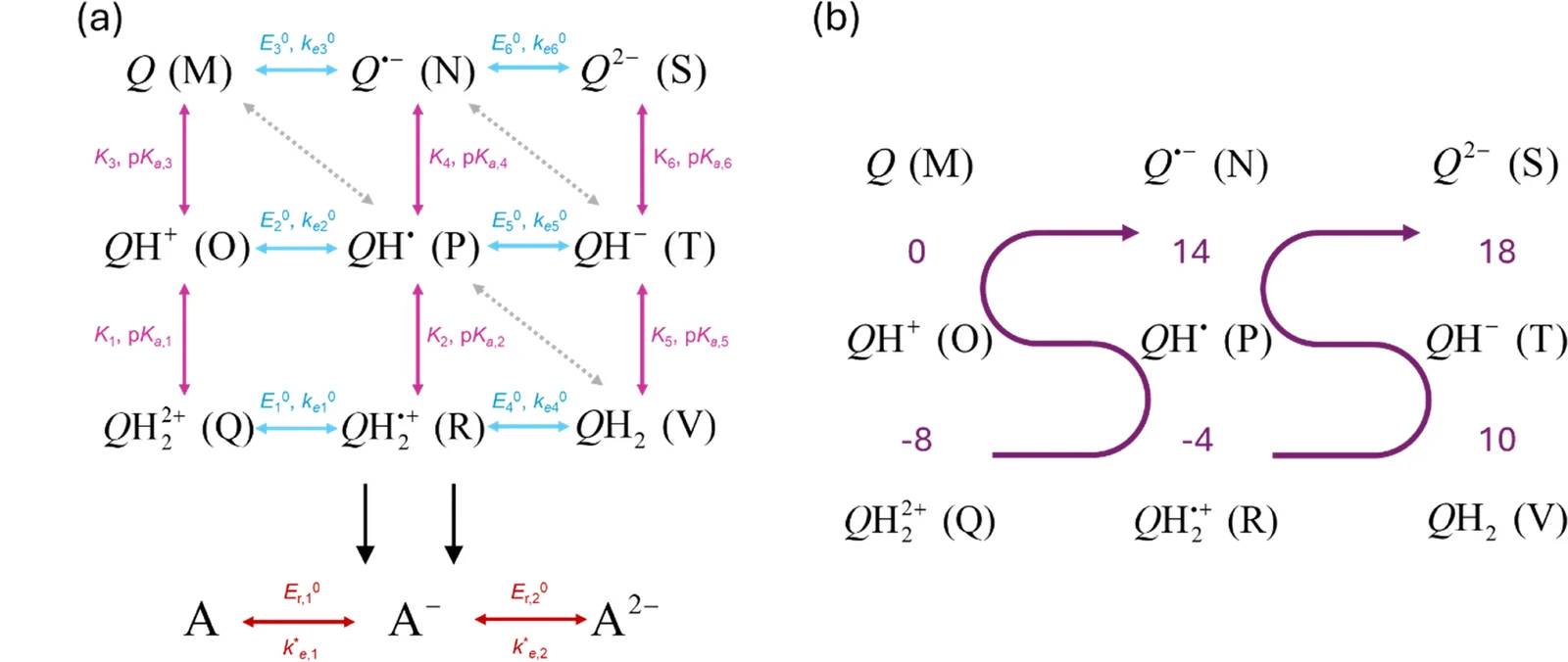
(a) Jacq “Scheme of Squares” model to illustrate the PCET reactions for a quinone molecule, both quinone notation, *e.g. Q*, and corresponding Laviron notation, shown in brackets (*e.g.* M) are used. (b) Example of an SS system and the p*K*_a,*n*_ values (purple) modelled in this work.

Whilst this model focuses on the 2e^−^2H^+^ PCET reaction of the quinone molecule, it can be applied to other 2e^−^2H^+^ PCET reactions. The model can also be adapted for smaller schemes *e.g.* 1e^−^1H^+^ (as also modelled, *vide infra*) such as hydrogen atom transfer,^30^ transition metal redox electrochemistry,^31^ or larger ones *e.g.* up to 6e^−^8H^+^ as in nitrogen fixation.^32^ The basis of the model originates from Jacq's “Scheme of Squares”,^16^Fig. 1a, which shows the nine distinct states an individual PCET molecule can exist in at any one time, here illustrated for a quinone, *Q*, top left (referred to as species M in the original notation used by Laviron; oxidised and fully deprotonated),^33^ moving to *Q*H_2_, bottom right (species V, Laviron notation; reduced and fully protonated) formed *via* addition of 2e^−^ and 2H^+^. The ET reactions move the quinone molecule horizontally between species (*e.g. Q* → *Q*^2−^), and are controlled by the standard redox potential (*E*^0^_*n*_) of each individual 1e^−^ reaction. The proton transfer (PT) reactions move the quinone molecule vertically between species (*e.g. Q*H_2_^2+^ → *Q*) and are controlled by the acid dissociation constants (p*K*_a,*n*_ = −log_10_*K*_a,*n*_) as well as the equilibrium constants (*K*_*n*_) for the PT reactions (*K*_*n*_ = [H^+^]_surf_/*K*_a,*n*_, where [H^+^]_surf_ is the proton concentration at the electrode surface).

When describing PCET reaction mechanisms, the terms “stepwise” and “concerted” are typically used. Stepwise mechanisms are displayed as solid blue and solid pink double-headed arrows in Fig. 1a, while concerted mechanisms are displayed as light grey, dashed double-headed arrows. A stepwise mechanism occurs when either an ET (blue) or PT (pink) reaction occurs individually and is indicated as any horizontal or vertical conversion of species respectively (*e.g. Q* → *Q*˙^−^ or *Q* → *Q*H^+^). A concerted mechanism occurs when an e^−^ and H^+^ come from the same bond in one of the reactants,^4^ and are therefore transferred simultaneously. Here a species directly converts into its diagonal neighbour (*e.g. Q* → *Q*H˙), with none of the adjacent species forming as intermediates. In this model, all ET and PT reactions are modelled individually with no prior assumptions made as to whether the mechanism is stepwise or concerted. Instead, the mechanism is determined by the reaction parameters provided as inputs to the model. For example, when ET and PT reactions are faster than the measurement timescale, no intermediate species are observed giving the appearance of concerted mechanisms.

The p*K*_a,*n*_ values for the PT steps are given in Fig. 1b. p*K*_a,*n*_ can take a range of values that are not always physically measurable. By considering each half of the “Scheme of Squares” (going left to right) the numerical order of p*K*_a,*n*_ values (from smallest to largest) of each vertical half (“ladder”) affects the final PCET mechanism. For example, the p*K*_a,*n*_ values presented in Fig. 1b, represent an ‘SS’ system, which shows a 2e^−^2H^+^ potential-pH relationship in the pH range 0–10.^19^ Experimental observation of 2e^−^2H^+^ for quinone PCET within a measurable pH range is common.^25,34^ In Fig. 1b, in the first half of the square, p*K*_a,1_ < p*K*_a,2_ < p*K*_a,3_ < p*K*_a,4_, whilst in the second half, p*K*_a,2_ < p*K*_a,5_ < p*K*_a,4_ < p*K*_a,6_. The purple arrows show the numerical order of the p*K*_a,*n*_ values and as shown, appear to form two letter S's, hence the terminology ‘SS’. Other PCET systems (*e.g.* ‘NN’, ‘NS’, and ‘SN’) are also described by Laviron,^19^ and could also be explored when adapting the model described herein.

The “Scheme of Squares” model assumes no dimerization of the intermediate radicals (N, P, and R), and no disproportionation (*i.e.* only simple 1e^−^ and 1H^+^ steps occur successively).^19^ When PT is assumed to be at equilibrium, the model can be further simplified by compressing the three rows into a two-step process comprising two, 1e^−^ reaction steps with rates 
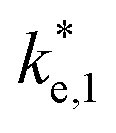
 and 
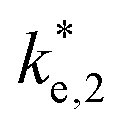
, and apparent standard redox potentials *E*^0^_r,1_ and *E*^0^_r,2_. The analytically determined values for these parameters, qualitatively follow values that have been obtained from experimental observations, as demonstrated by Laviron for the *p*-benzoquinone/hydroquinone system.^35^

### 2.2. General model information

Full details of the continuum model can be found in SI-1. A 1 dimensional (1D) geometry, Fig. S1, provides a simple model of a macroscale disk electrode covered uniformly by a defined coverage of quinone molecules (Γ_tot_, mol cm^−2^). The potential (*E*) waveform of the cyclic voltammetric (CV) simulation is described in SI-1.2. A list of the parameters, and their expressions, used for this model can be found in Table S1 and SI-1.3. The model describes mass transport using the Nernst-Planck equation (eqn (S3) and SI-1.4), and assumes diffusion-only conditions (*i.e.* constant temperatures, no stirring, and a supporting electrolyte in excess). However, migration,^36^ and convection can be added to the model if required. Mesh (Fig. S2) and convergence (Fig. S3) tests are found in SI-1.5 and SI-1.6 respectively. Table S2, SI-1.7 provides a list of p*K*_a_ values, ET rate constants, quinone monolayer surface coverages and diffusion coefficients (including literature sources) used in this model. Additional literature values are provided in Table S3, SI-1.7, for comparison. The COMSOL-generated model report is also provided as a separate file for use and adaptation.

### 2.3. Quinone reactions

The reactions happening at the quinone sites on the surface of the electrode can be separated into; (1) (ET) (*e.g.* M → N), and (2) PT reactions (*e.g.* M → O). These separate reactions can be summed to provide a total surface reaction (3.3) for each species.

To observe the law of mass conservation, eqn (1) must be observed at all times, as each quinone can only exist in one state (M to V) at any point in time.

1 Γ_tot_ = Γ_M_ + Γ_N_ + Γ_S_ + Γ_O_ + Γ_P_ + Γ_T_ + Γ_Q_ + Γ_R_ + Γ_V_

#### 2.3.1. Electron transfer reactions

An example of a redox ET reaction scheme equation for the quinone states in the left-most column (M, O, Q), is given in eqn (2); here illustrated for M. The rate equation found from this scheme equation is provided in eqn (3). Similar equations can be written for the right-most column (S, T, V).2
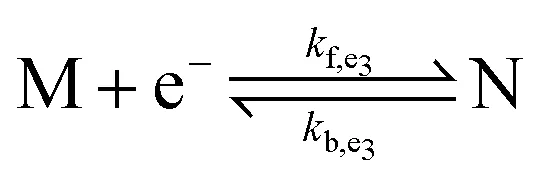
3
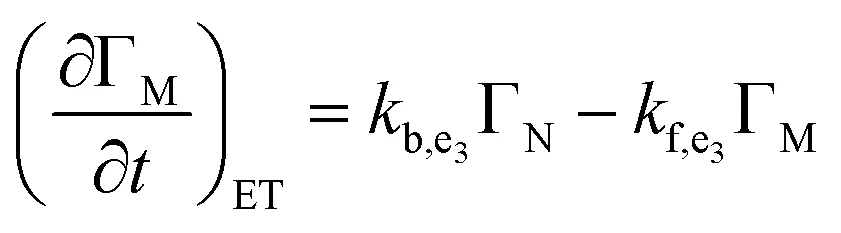


Eqn (3) shows the change in surface coverage, Γ_*i*_, of the quinone state over time due to electron gain/loss. The forward (*k*_f,e_*n*__, reduction) and backward (*k*_b,e_*n*__, oxidation) ET reaction rate constants are defined by Butler–Volmer kinetics, eqn (4) and (5).^37^

4 *k*_f,e_*n*__ = *k*^0^_e,*n*_exp[−α*f*(*E* − *E*^0^_*n*_)]

5 *k*_b,e_*n*__ = *k*^0^_e,*n*_exp[(1 − α)*f*(*E* − *E*^0^_*n*_)]

where *k*^0^_e,*n*_ is the heterogeneous rate constant for the surface-bound quinone system of interest, *α* is the charge transfer coefficient, and 
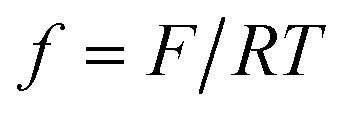
 where *F* is Faraday's constant, *R* is the ideal gas constant, and *T* is the temperature. The *E*^0^_*n*_ values used for this model are described by eqn (6)–(11). Here, *E*^0^_2_ is the only potential assigned a value in the model, in this case 0.25 V, and a potential difference of 0.5 V between *E*^0^_2_ and *E*^0^_5_ is chosen to mimic the ‘SS’ 2e^−^2H^+^ system described by Laviron (Fig. 7 in ref. 19) and to centre the voltammetric response ∼0 V. To the experimentalist, *E*^0^_2_ is the redox potential for *Q*H^+^ → *Q*H˙ and *E*^0^_5_ is the redox potential for *Q*H˙ → *Q*H^−^ (travelling across the middle row of the scheme of squares). Between these potentials is where 2e^−^2H^+^ PCET occurs. In this work, everything is modelled with respect to an arbitrary reference at 0 V.6
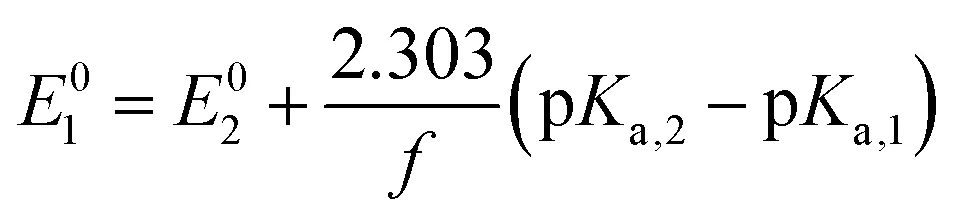

7 *E*^0^_2_ = 0.25 V

8
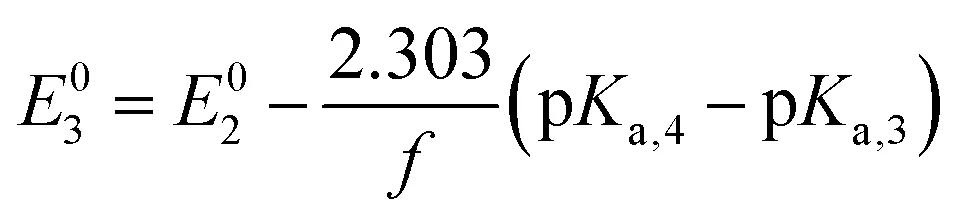
9
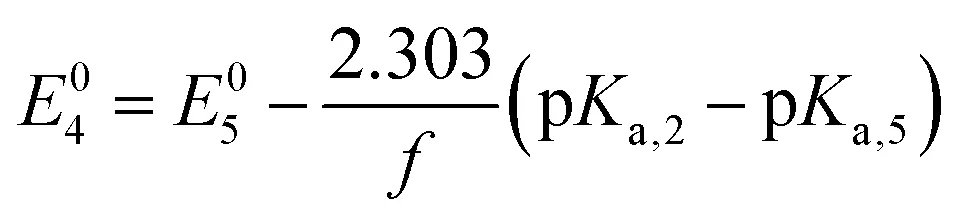

10 *E*^0^_5_ = *E*^0^_2_ − 0.5 V

11
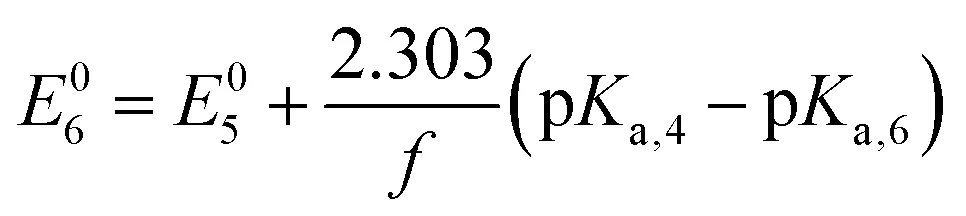


The change in surface coverage of the central species (in the scheme of squares) N, P, and R can be written in terms of the change in surface coverage of the species either side of them, demonstrated for species N in eqn (12).12
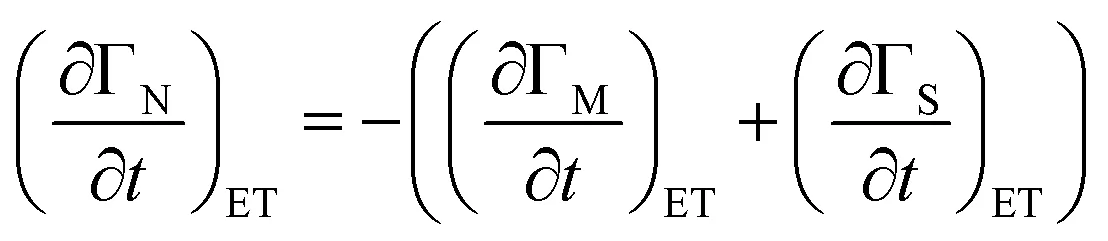


For all individual species ET rate equations see SI-2.

#### 2.3.2. Proton transfer reactions

As for the ET reactions, an example of PT reaction equations for the top row (M, N, S), is demonstrated in eqn (13) for species M. A corresponding rate equation, eqn (14), can be written where the rate of change of Γ_*i*_ due to the addition/loss of H^+^ is described. Similar equations can be written for the bottom row (Q, R, V).13
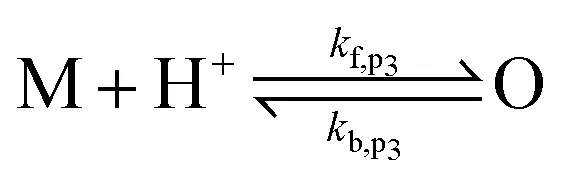
14
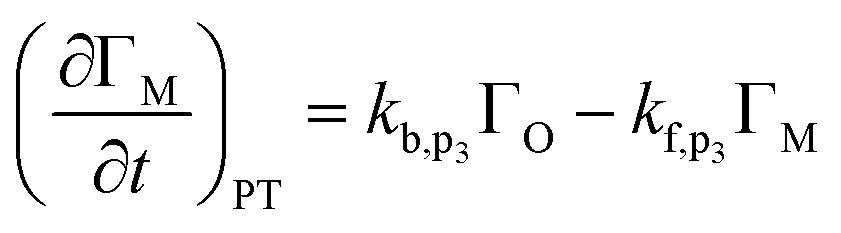


[H^+^]_surf_ is the concentration of H^+^ at the electrode surface (*x* = 0). The backwards (deprotonation, *k*_b,p_*n*__) rate constant is initially set to 1 × 10^9^ s^−1^ (based on ref. 38), which means deprotonation is assumed to be very fast. The forwards (protonation, *k*_f,p_*n*__) rate constant is given by eqn (15), and *K*_*n*_ is given by eqn (16), where *K*_a,*n*_ is the acid dissociation constant of the respective proton reaction (Fig. 1a), and [H^+^]_surf_ is the concentration of H^+^ at the electrode surface (*x* = 0). It is equally possible to set *k*_f,p_*n*__ first and determine *k*_b,p_*n*__*via*eqn (15) and (16). All p*K*_a,*n*_ values used for this model can be found in Fig. 1a. *k*_f,p_*n*__ therefore depends on [H^+^]_surf_ (*i.e.* the availability of protons at the electrode surface) and *K*_a,*n*_. In certain cases, this leads to the proton reaction rate being the rate limiting reaction (*vide infra*).

15 *k*_f,p_*n*__ = *k*_b,p_*n*__*K*_*n*_

16
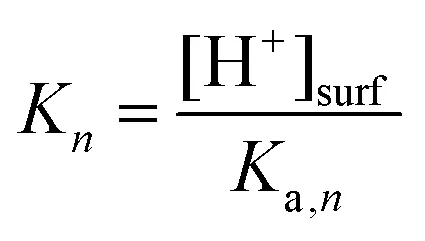


Similarly to the ET reactions, the change in surface coverage of the centre species O, P, and T can be written in terms of the change in surface coverage of the species above and below it in the “Scheme of Squares”, this is demonstrated for species O in eqn (17).17
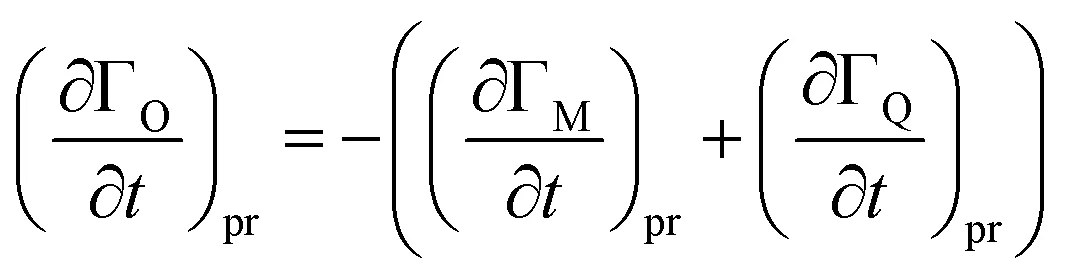


The PT rate equations for all species can be found in SI-3.

##### 2.3.2.1. Buffered solutions

For an ideal buffered solution it is assumed that the H^+^ concentration is the same everywhere and the pH at the electrode surface is the same as in bulk solution. Therefore, the rate of change of [H^+^]_surf_, can be set to 0. For a 1D model, as used herein, this is done by setting the flux of H^+^ normal to the electrode surface, **J**_H^+^_, to 0, eqn (18), where **n** is the unit vector normal to the electrode surface.

18 −**n**·**J**_H^+^_ = 0

##### 2.3.2.2. Unbuffered solutions

In an unbuffered solution, [H^+^]_surf_ deviates from the bulk pH as H^+^ are removed/added during the protonation/deprotonation PCET reactions respectively.^29,39^ Here, **J**_H^+^_ is determined by the change of species M, N, S to O, P, T respectively, in addition to the change of O, P, T to Q, R, V respectively (PT reactions). This can be written using the rate of change of species M, N, S, Q, R, and V, as in eqn (19).19
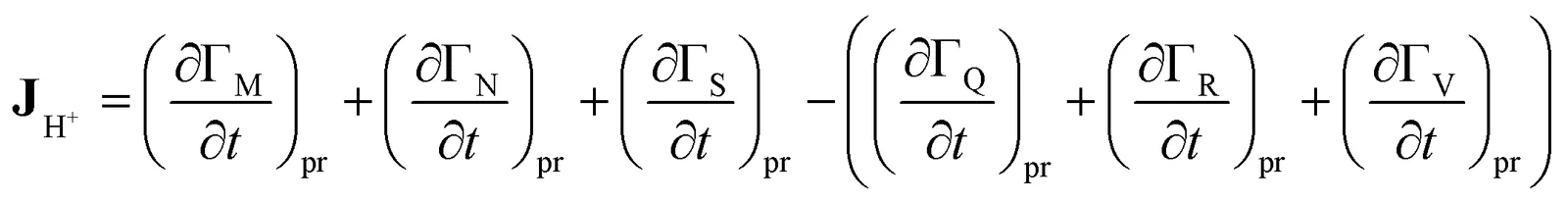


As the basicity of a solution increases, the decreased concentration of H^+^ in unbuffered solution means any perturbation of H^+^ at the electrode surface due to quinone reduction (involving proton transfer) is emphasised. Additionally, as the pH increases, the H^+^ donor switches from the hydronium ion (H_3_O^+^) to water, which itself acts as a weak H^+^ donor *via* the self (or auto) ionisation of water reaction, eqn (20).20
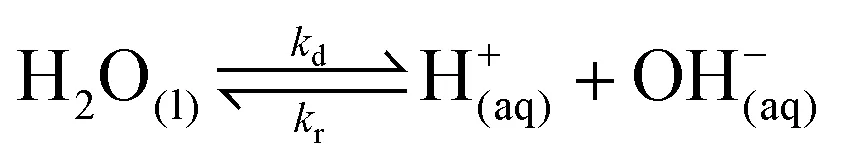


This accounts for the very weak buffer capacity of water. The self-ionisation of water reaction is not always considered in models, and when included is often represented as a simple equilibrium reaction which neglects the finite kinetics of the reaction.^39,40^ The importance of the self-ionisation of water reaction is explored in further detail *vide infra*. In eqn (20), *k*_d_ represents the rate of dissociation of water (= 2.6 × 10^−5^ s^−1 ^^41^) and *k*_r_ is the rate of recombination (= 1.4 × 10^11^ dm^3^ mol^−1^ s^−1 ^^42^). The total chemical reaction rate, *R*_w_, for the formation of both H^+^ and OH^−^ from H_2_O is given by eqn (21).

21 *R*_w_ = *k*_d_[H_2_O] − *k*_r_[H^+^][OH^−^]

### 2.4. Total reaction

The total change in species concentration can be calculated by simply adding the change due to ET reactions with the change due to PT, eqn (22), for all species M to V.22
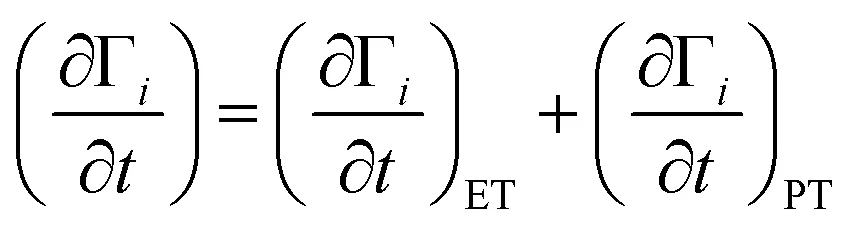


The current density, *j*, is related to the rate of change of the Γ_*i*_ with time (*t*) using eqn (23). A plot of *j versus E* provides the final simulated CVs. Here, *j* is linked to *E via* the scan rate (*ν*), and is therefore linked by time (*t*).23
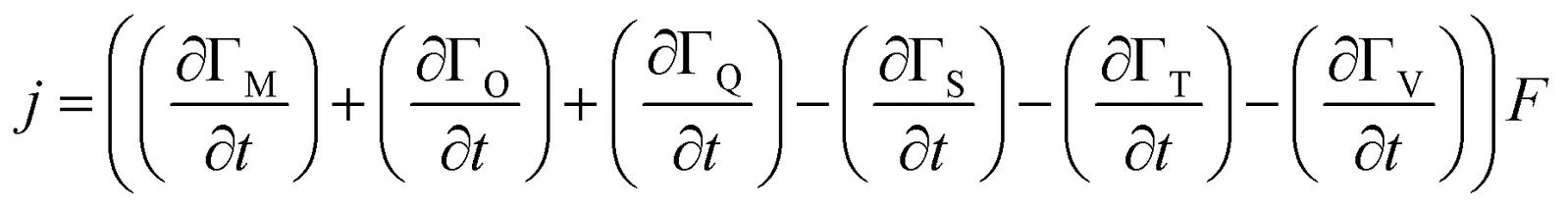


## 3. Results and discussion

### 3.1. Fast electron transfer in buffered solutions at monolayer coverage

**Fig. 2:**
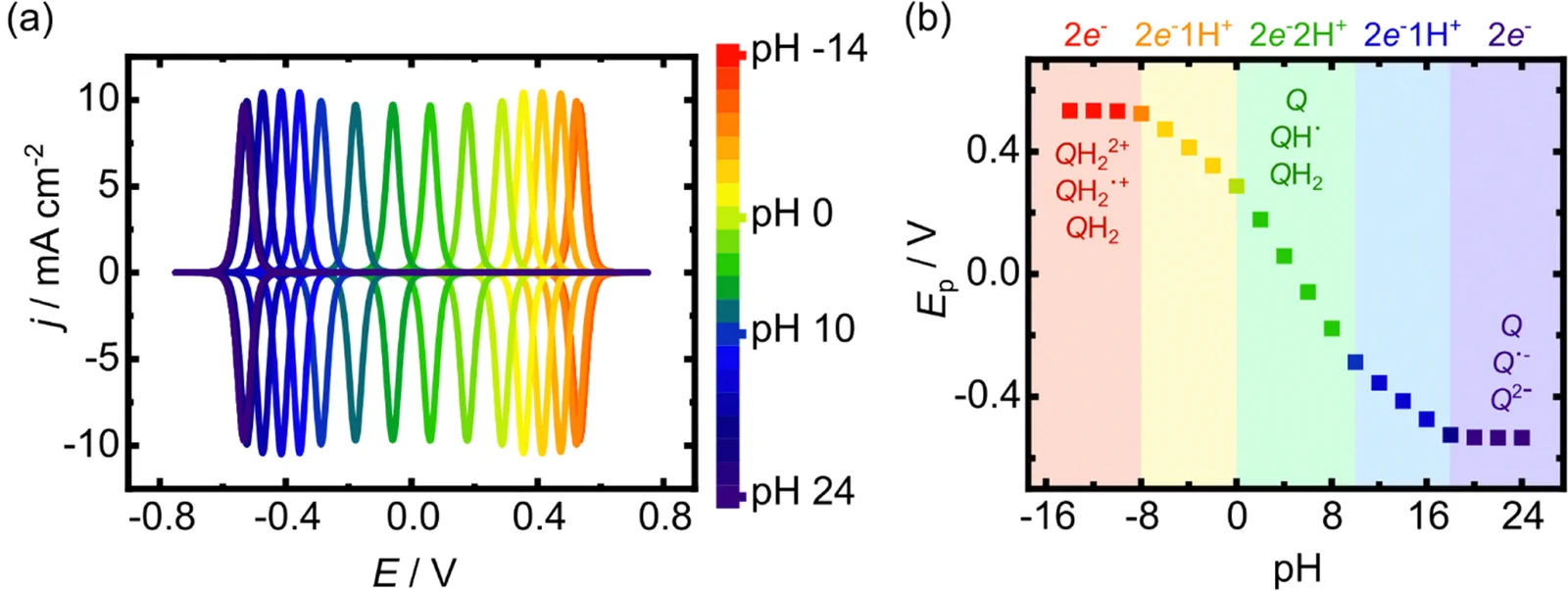
(a) Simulated CV's (*ν* = 0.1 V s^−1^) in buffered solution from pH −14 to 24, Γ_tot_ = 2.8 × 10^−10^ mol cm^−2^ (monolayer coverage), *k*^0^_e,*n*_ = 1 × 10^6^ s^−1^, *T* = 298.15 K. (b) Plot of the averaged *E*_p_ of the simulated CVs as a function of solution pH.

To determine the validity of the model, CVs for a monolayer quinone surface coverage (= 2.8 × 10^−10^ mol cm^−2^ from reference,^43^ Table S2, SI-1.7) and pH values from −14 to +24, were simulated under buffered conditions for *ν* = 0.1 V s^−1^, Fig. 2a. Though practically unrealistic, this pH range covers all the p*K*_a,*n*_ values (p*K*_a,1_ to p*K*_a,6_) originally used by Laviron for the SS equilibrium system,^19^ and in later work by Menshykau *et al*.^20^ For a reversible surface-bound reaction, the peak separation (Δ*E*_p_) for the anodic (*E*_p,a_) and cathodic (*E*_p,c_) peaks is ∼0, when *ν* is low,^18^ as shown in Fig. 2a. The surface-bound species is able to undergo fast ET free from diffusional limitations.^37^ As pH increases, from pH −14, the position of *E*_p_, determined by averaging *E*_p,a_ and *E*_p,c_, shifts negatively (Fig. 2a).

*E*_p_ plotted against pH is shown in Fig. 2b. The slope, which is Nernstian in nature, varies with pH. At sufficiently high or low pH values the slope is 0 mV pH^−1^ due to ET only (2e^−^), which occurs when either pH < p*K*_a,1_ (= −8, red) or pH > p*K*_a,6_ (= 18, purple). The stable quinone states at the different pH values are also shown on Fig. 2b. As the pH is decreased from high or increased from low values, the slope moves from 0 to −29.5 mV pH^−1^ (at 298.15 K) due to 2e^−^1H^+^; the pH is now in the range p*K*_a,1_ to p*K*_a,3_ (−8 to 0, yellow) or p*K*_a,5_ to p*K*_a,6_ (10 to 18, blue). Finally, when the pH is in the range p*K*_a,3_ to p*K*_a,5_ (0 to 10, green), the slope moves to −59.2 mV pH^−1^ (at 298.15 K) as a result of 2e^−^2H^+^. The simulated slope values presented in Fig. 2 agree with theory.^19^ A comparison of the system modelled in this work using finite element simulations *versus* that modelled numerically by Laviron,^19^ in the 2e^−^2H^+^ region is provided in Fig. S4, SI-4. Their similarity offers confidence to the PCET model developed here. The exact slope values can be found in Fig. S5, SI-5.

**Fig. 3:**
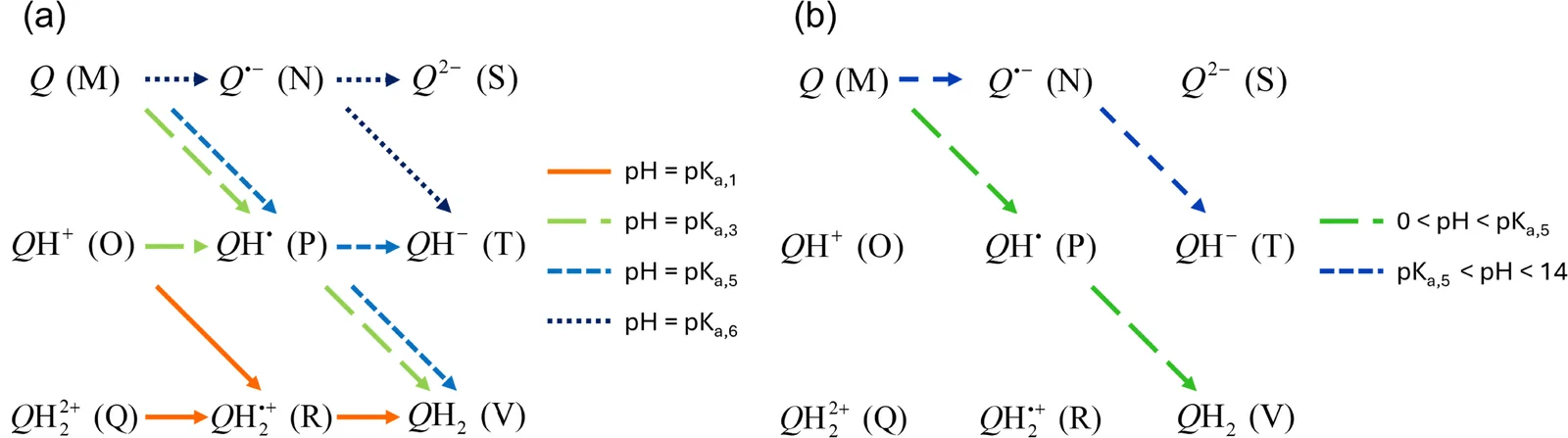
(a) The mechanism pathways when pH = p*K*_a,1_ (−8), p*K*_a,3_ (0), p*K*_a,5_ (10), or p*K*_a,6_ (18). (b) The mechanism pathways when 0 < pH < p*K*_a,5_ or p*K*_a,5_ < pH < 14.

The pathway that the quinone molecule takes through the “Scheme of Squares” during the CV changes depending on the pH of the buffer solution. Fig. S6 and SI-6 demonstrates the reaction pathway assuming theoretical pH values equal to p*K*_a,1_ (= −8), p*K*_a,3_ (= 0), p*K*_a,5_ (= 10) and p*K*_a,6_ (= 18) by following Γ_*i*_ for each species over time. These pathways are shown in Fig. 3a, when scanning reductively. When the pH is equal to p*K*_a,*n*_, there is equal splitting of the quinone species, in terms of surface concentration, either side of the reaction (*e.g. Q* and *Q*H^+^, when pH = p*K*_a,3_ = 0, green dashed arrow in Fig. 3a). As the reducing potential drives the reaction (moving from left to right through the “Scheme of Squares”) the dominant species is determined by the interfacial pH (= solution pH when in buffered solution) and p*K*_a,*n*_ values. For pH < p*K*_a,5_, *Q*H_2_ (V) is favoured, for p*K*_a,5_ < pH < p*K*_a,6_, *Q*H^−^ (T) is favoured, and for pH > p*K*_a,6_, *Q*^2−^ (S) is favoured. Notice however that none of the species move vertically, only diagonally and horizontally indicating you cannot drive PT without ET.

In the more realistic pH range of 0 – 14, reaction pathways predominantly follow the mechanisms in Fig. 3b, depending on whether the pH is greater or smaller than p*K*_a,3_ and p*K*_a,5_. The PCET reactions observed in the pH range 0–10 are concerted, as the individual reaction steps occur so quickly that the intermediate species are immediately used for the next step. See Fig. S7 and SI-6 for Γ_*i*_ as a function of time plots in the pH range 2–14. The very reactive quinone species *Q*^2−^ (S) and *Q*H_2_^2+^ (Q) do not form in the pH range 0–14.

### 3.2. Finite electron transfer in buffered solutions at monolayer coverage

**Fig. 4:**
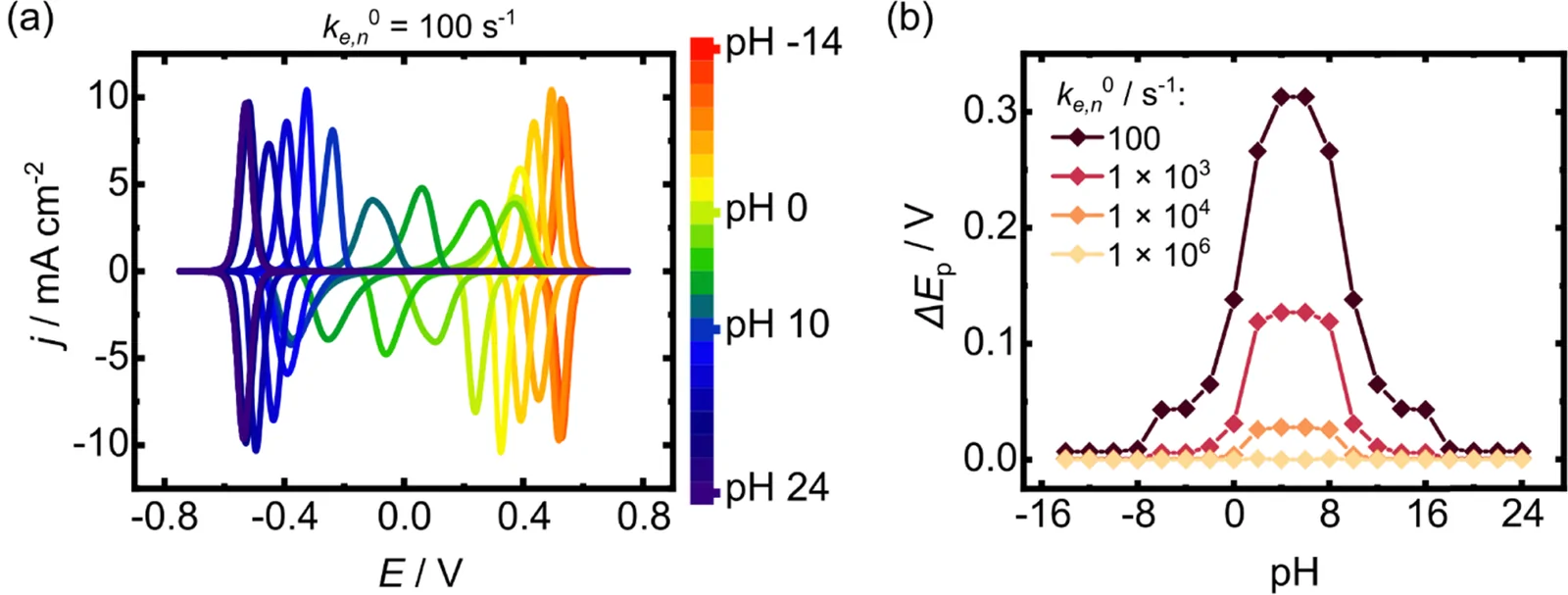
(a) Simulated CVs (*ν* = 0.1 V s^−1^) in buffered solution from pH −14 to 24, Γ_tot_ = 2.8 × 10^−10^ mol cm^−2^ (monolayer coverage), for *k*^0^_e,*n*_ values of 100 s^−1^. (b) Δ*E*_p_ as a function of pH for *k*^0^_e,*n*_ values of 100, 1 × 10^3^, 1 × 10^4^ and 1 × 10^6^ s^−1^. Lines in (b) are for visualisation purposes only.

The intrinsic ET rate constant for each step of the PCET reaction, *k*^0^_e,*n*_, is an important parameter when interpretating and comparing PCET reactions. In Fig. 2a, ET for all steps was reversible given the very high *k*^0^_e,*n*_ values employed (1 × 10^6^ s^−1^). Fig. 4a shows the simulated CV response in buffered solutions when *k*^0^_e,*n*_ is reduced by four orders of magnitude to 100 s^−1^ for *ν* = 0.1 V s^−1^. Δ*E*_p_ increases in size especially when p*K*_a,3_ < pH < p*K*_a,5_*i.e.* when 0 < pH < 10 (2e^−^2H^+^). Fig. 4b shows how Δ*E*_p_ changes as a function of pH and *k*^0^_e,*n*_, from 100 to 1 × 10^6^ s^−1^. As *k*^0^_e,*n*_ decreases, Δ*E*_p_ increases, with the largest Δ*E*_p_ again found in the pH range 0–10 (p*K*_a,3_ – p*K*_a,5_). Physically, the conversion of *Q* to *Q*H_2_ takes longer to complete at slower *k*^0^_e,*n*_ values, and oxidation and reduction reactions show peak broadening.

The observed change in Δ*E*_p_ with pH indicates that whilst the intrinsic ET rate constants, *k*^0^_e,*n*_, of the quinone are pH independent, the apparent ET rates of the system are pH dependent. One method used by Laviron to describe these apparent rate constants with respect to pH is *via* the analytical expressions, eqn (24) and (25),^19^ determined when modelling the PCET mechanism using the simplified “Scheme of Squares” model (A → A^−^ →A^2−^, Fig. 1a).24
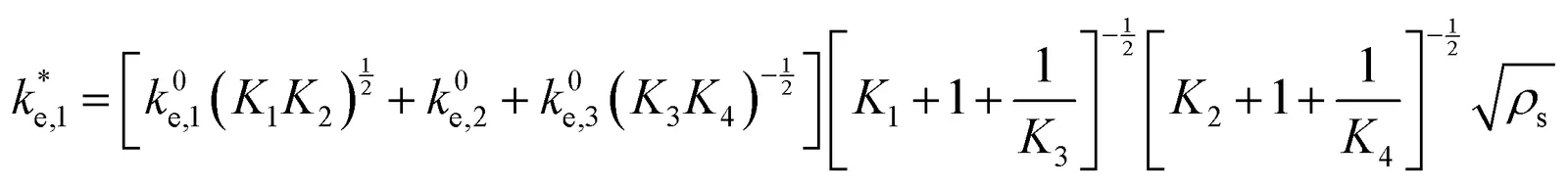
25
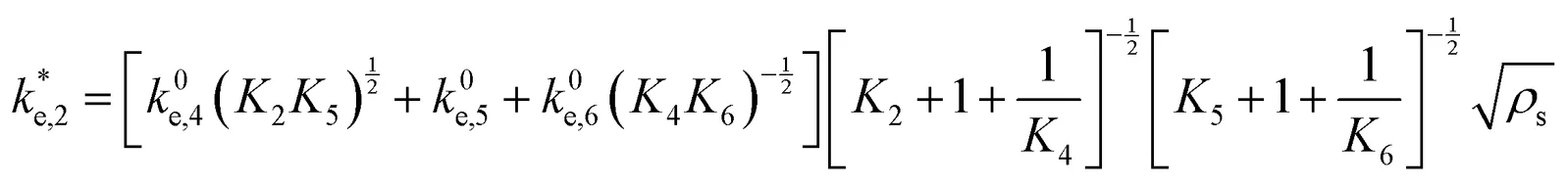
where:26
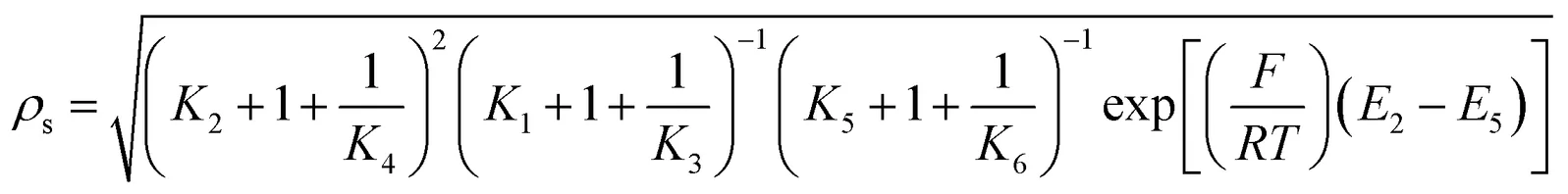

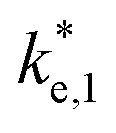
 and 
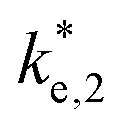
 describe the two individual 1e^−^ ET steps, and depend on the intrinsic *k*^0^_e,*n*_ values as well as *K*_*n*_ values (which are pH dependant). *ρ*_s_ represents the square root of the constant for semiquinone formation,^44^ and includes values for the apparent standard potentials of the system *E*_r,1_ and *E*_r,2_ (see SI-7 for equations), which are the redox potentials of the two separate steps.

**Fig. 5:**
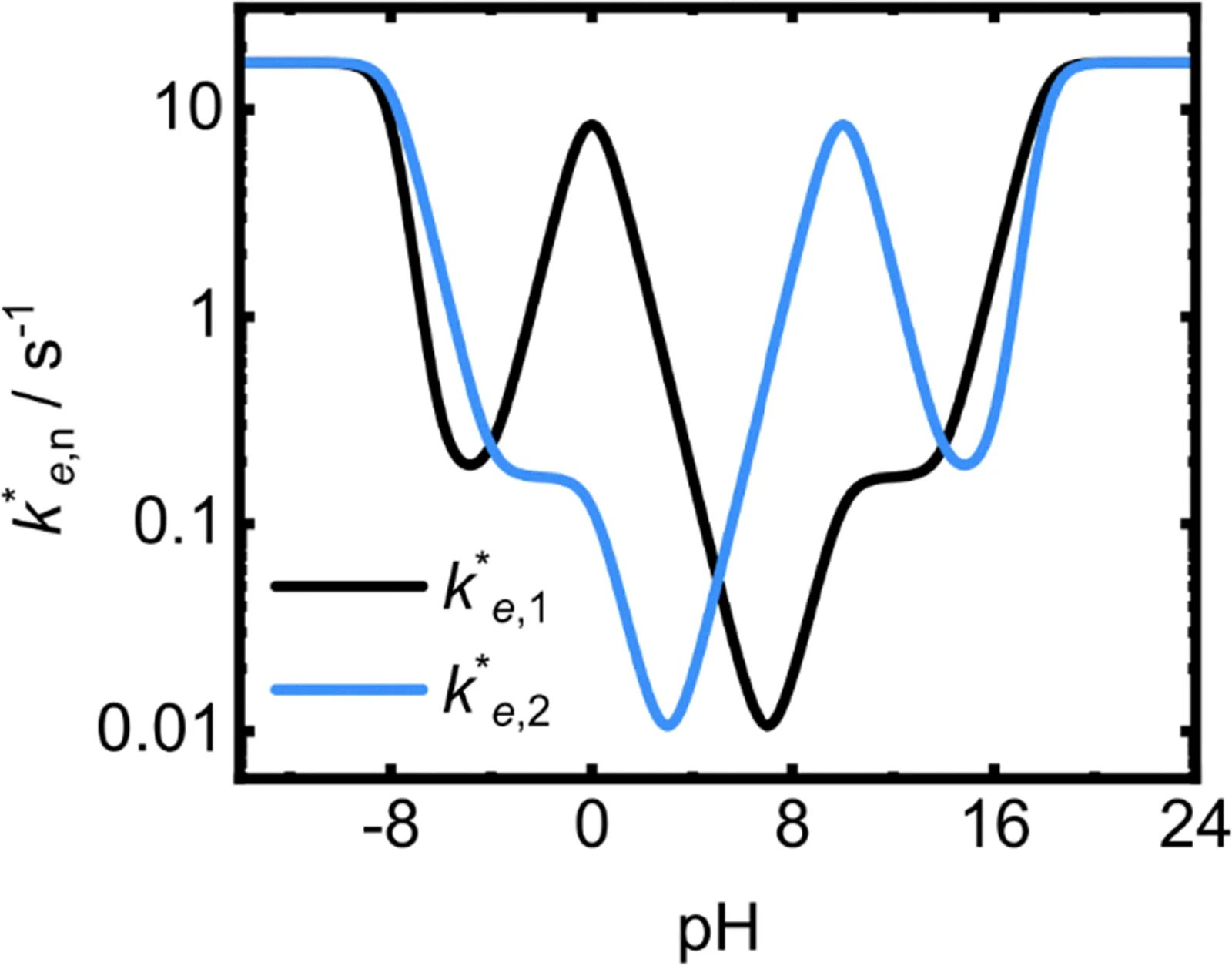
Plot of 
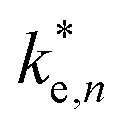
*vs.* pH (assuming buffered conditions) for *k*^0^_e,*n*_ values of 100 s^−1^.

Fig. 5 shows that these analytically derived apparent ET rate constants, 
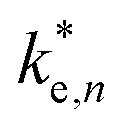
, vary significantly over the pH range −14 to 24 (assuming *k*^0^_e,*n*_ = 100 s^−1^, see Fig. S8, SI-8 for 
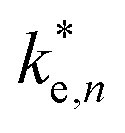
 plots for *k*^0^_e,*n*_ values in the range 500 to 1 × 10^6^ s^−1^). The fastest 
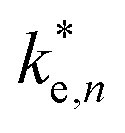
 occurs at very low (<−8) and very high (>18) pH and this maximum is 
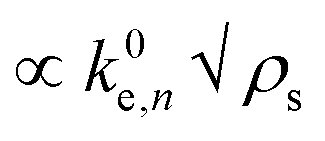
. Over the practical pH range (pH 0–14), the apparent ET rate constants still vary appreciably, decreasing from a maximum of 10 s^−1^ to a minimum of 0.026 s^−1^ between pH 3–7. This reflects the peak shapes observed in Fig. 4a where sharp, symmetrical peaks are observed for very low (<−8) and very high (>18) pH values, and larger peak separation and asymmetry is apparent over the practical pH range.

**Fig. 6:**
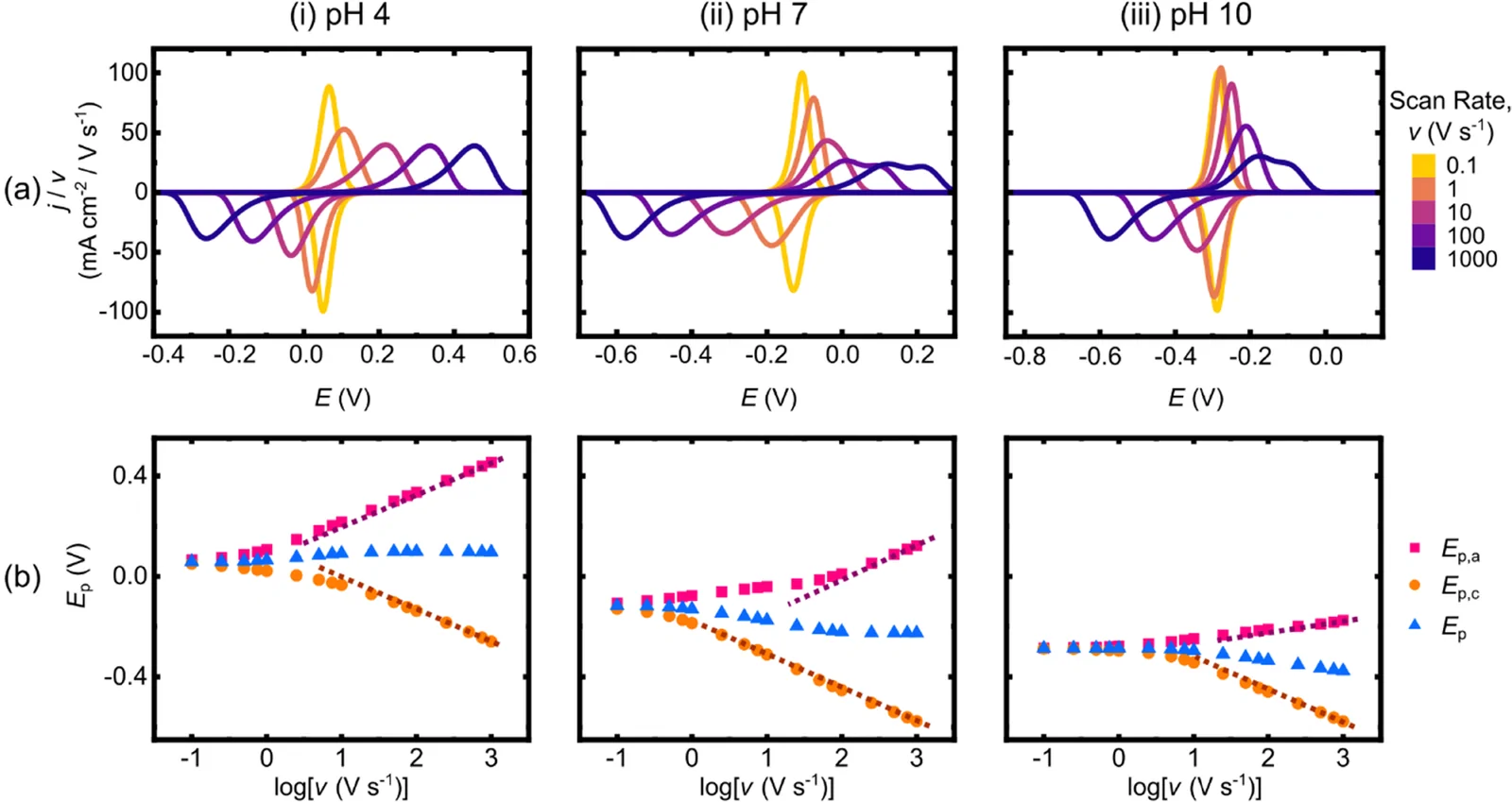
(a) Simulated CV scans (1 V potential range) at varying *ν* (0.1–1000 V s^−1^) with *j* normalised by *ν*, and (b) Laviron plots (0.1–1000 V s^−1^) for buffered (i) pH 4, (ii) pH 7, and (iii) pH 10. *k*^0^_e,*n*_ = 2 × 10^4^ s^−1^.

Laviron also described the use of peak potential *versus* log *ν* plots often referred to as Laviron plots or trumpet plots,^18^ to extract apparent ET rate constants, *k*_expt_ from CV experiments, for surface bound redox couples undergoing single step electron transfer. From plots of *E*_p,*a*_ (*a* = anodic) and *E*_p,*c*_ (*c* = cathodic) *versus* log *ν*, both *k*_expt_ and *α*, can be obtained. Whilst introduced for diffusion-less, single step electron transfer reactions, this approach has also been used in the literature to extract *k*_expt_ for surface bound quinone PCET systems, with values varying typically between 1 to 1000 s^−1^.^23,25,29,39,43,45–48^ It is thus useful to consider how analytically derived 
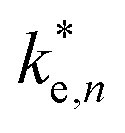
 compares to *k*_expt_ when applied to quinone systems given *k*_expt_ was postulated for use only with ET systems. Consideration was given to both a 1e^−^1H^+^ and 2e^−^2H^+^ system and an experimentally realistic *k*^0^_e,*n*_ = 2 × 10^4^ s^−1^ value was employed for all simulations.^29^ Details of the 1e^−^1H^+^ model are provided in SI-9, including the analytical equation used to determine 
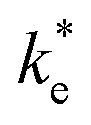
 (eqn (S28)). CVs for both a 1e^−^1H^+^ (Fig. S10, SI-10) and 2e^−^2H^+^ PCET mechanism (Fig. 6) were simulated, over the pH range 0–14; the current is now represented as the current density, *j*, normalised by *ν* to present the CV plots on the same scale. From this data the gradients, *m*, of the dotted pink (anodic) and orange (cathodic) lines in Fig. 6b are used to calculate *α* (eqn (27)), which is then used to determine *k*_expt_ (eqn (28) and (29) for the anodic and cathodic components respectively).27
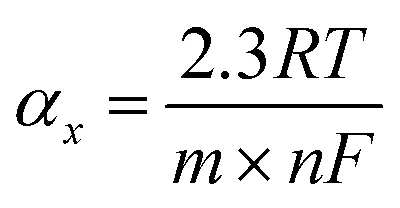
28
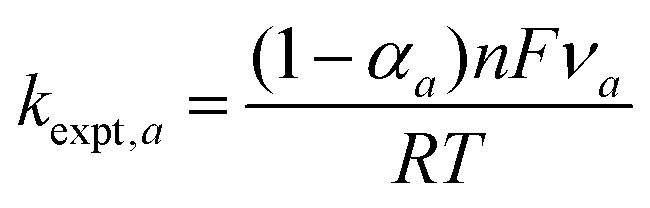
29
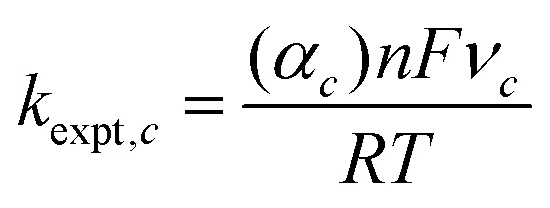
where *ν*_*x*_ is the scan rate at which the asymptote (from which *m* is found) crosses *E*_p_ (blue triangles).

As expected, *j* for both the anodic and cathodic peaks increase as *ν* increases, as does Δ*E*_p_. For the 1e^−^1H^+^ system there is no peak splitting (where one peak deconvolutes into two separate peaks) but some peak broadening is observed at fast scan rates. This indicates that PT is not rate limiting. The effect of lowering *k*_b,p_*n*__ is shown in Fig. S11, SI-11, where peak splitting is now evident. For the 2e^−^2H^+^ system, peak splitting of *E*_p,a_ can be seen for pH > 7, even with a high value for *k*_b,p_*n*__ (= 1 × 10^9^ s^−1^), at fast *ν* (1000 V s^−1^). The shorter experimental timescale separates processes where *Q* → *Q*H˙ is converted at a faster rate than *Q*H˙ → *Q*H_2_ (Fig. S13, SI-12). Even though *k*_b,p_*n*__ is high, slower rates of protonation result, in accordance with eqn (15) and (16), given the wider range of p*K*_a,*n*_ values in the 2e^−^2H^+^ system.

All values for *α*, and *k*_expt_ found from simulated Laviron plots are presented in SI-13 for both the 1e^−^1H^+^ (Table S4) and 2e^−^2H^+^ (Table S5) system. For the 1e^−^1H^+^ system, scan rates of up to 1 × 10^6^ V s^−1^ were required to obtain positive values for *α*. The same was true for the 2e^−^2H^+^ system for pH −10 and +20, whilst scan rates of 1000 V s^−1^ were sufficient for pH values in the range 0–14. For the 1e^−^1H^+^ system, *α* values of *ca.* 0.5 are determined, which is expected for a single-step 1e^−^ reversible reaction, again indicating that the influence of the PT step for 1e^−^1H^+^ is negligible. For a 2e^−^ (in two 1e^−^ steps) system, *α* values of 0.25 or 0.75, depending on whether the 1st or 2nd step is rate limiting, should result.^49^ This is observed for the 2e^−^2H^+^ system at extreme pH values of −10 and +20. However, inside this range, large deviations for *α* are observed, again suggesting that the PT steps are much more influential.

**Fig. 7:**
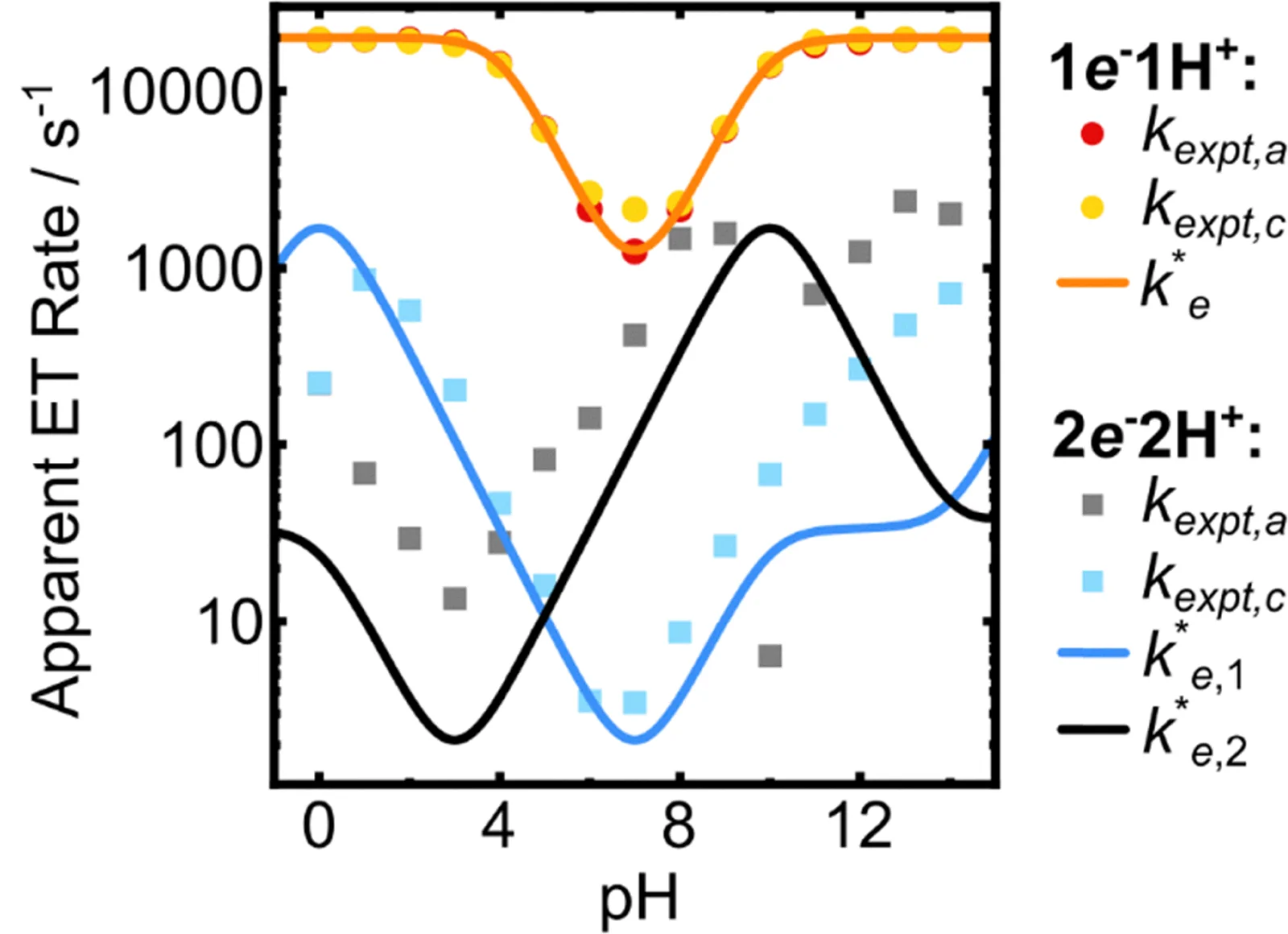
Comparison of *k*_expt_ (scatter plots) to 
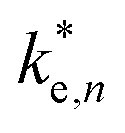
 (line graphs) for both the 1e^−^1H^+^ (circles) and 2e^−^2H^+^ (squares) systems, with *k*^0^_e,*n*_ = 2 × 10^4^ s^−1^.

*k*_expt_ values were compared to 
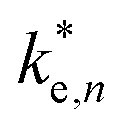
, calculated using eqn (S28), SI-9 for 1e^−^1H^+^, and eqn (24)–(26) for 2e^−^2H^+^, the results of which are presented in Fig. 7 for the pH range 0–14. For the 1e^−^1H^+^ system, *k*_expt_ closely matches 
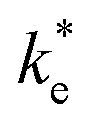
, suggesting that the Laviron plot method provides an accurate depiction of the apparent ET rate constant for a 1e^−^1H^+^ system provided that the PT rates are fast (see SI-11). The more complex 2e^−^2H^+^ mechanism shows less agreement, although the simulated *k*_expt_ values appear to follow the 
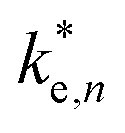
 trend, especially within the pH range 0 to 10. *k*_expt,*a*_ more closely follows 
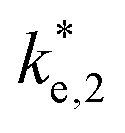
, while *k*_expt,*c*_ follows 
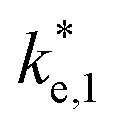
. Similar observations were made by Laviron when comparing 
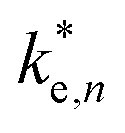
 to *k*_expt_ for benzoquinone;^33^ although here *k*_expt_ was determined from reaction half-life times.^33^ The higher 
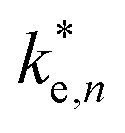
 values for the 1e^−^1H^+^ reaction, compared to 2e^−^2H^+^ (Fig. 7) also explains why a faster scan rate was required to see kinetic limitations for 1e^−^1H^+^*versus* the 2e^−^2H^+^ system over the same pH range.

Deviations between 
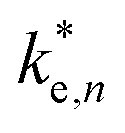
 and *k*_expt_ for 2e^−^2H^+^ again indicate PT is becoming rate limiting meaning that the assumptions required when using the Laviron plot method are no longer upheld. Additionally, given the peak separates into two it is difficult to know the correct peak potential to use. For Fig. 7, a MATLAB (MathWorks®) script was employed that simply picks the maximum current value in order to determine *E*_p_, which may not be representative of the true *E*_p_ of the 2e^−^2H^+^ quinone oxidation/reduction reaction.

### 3.3. Fast electron transfer in unbuffered solutions

Experimentally, quinone modified electrodes have found use as voltammetric pH sensors for environmental/medical applications *e.g.* drinking water, sea water, blood *etc.* They offer an attractive alternative to the glass pH probe due to *e.g.* improved robustness,^15^ ease of miniaturisation,^50^ and multiple analyte detection.^51^ However, they provide inaccurate measurements in unbuffered solutions, especially in the pH range 6–8,^29^ which is problematic for use in environmental and biological solutions. To understand why this happens and how to mitigate the problem, simulations were carried out in unbuffered solutions.

**Fig. 8:**
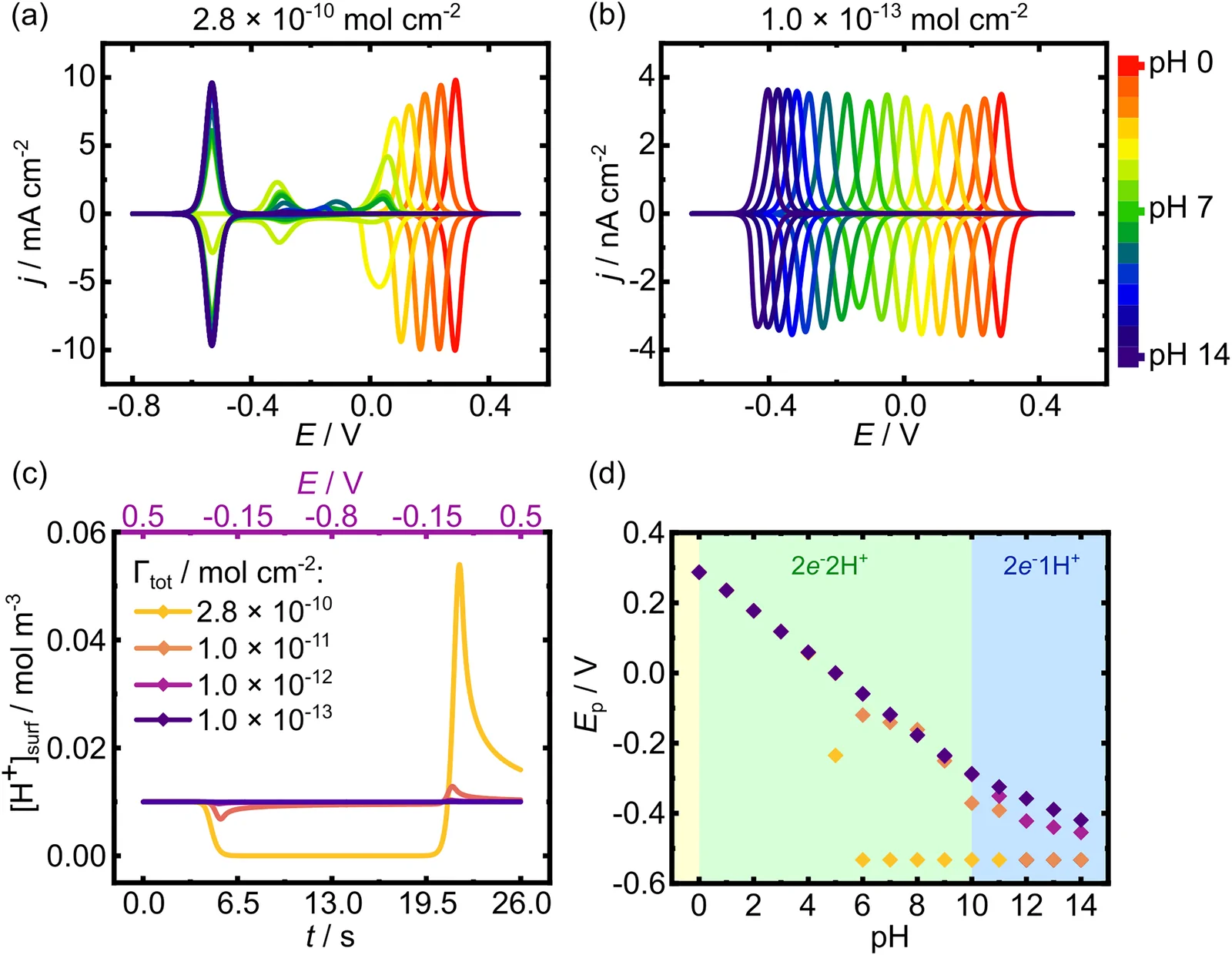
Simulated CVs (0.1 V s^−1^) in unbuffered solution from pH 0–14 when Γ_tot_ is (a) 2.8 × 10^−10^ mol cm^−2^ and (b) 1 × 10^−13^ mol cm^−2^. (c) [H^+^]_surf_*vs.* time plot at pH = 5, and (d) *E*_p_*vs.* pH calibration plot, for Γ_tot_ values of 2.8 × 10^−10^ (yellow square), 1 × 10^−11^ (orange circle), 1 × 10^−12^ (pink diamond), and 1 × 10^−13^ (purple triangle) mol cm^−2^. *k*^0^_e,*n*_ = 2 × 10^4^ s^−1^.

For a sensor it is important that the largest change in *E*_p,a_ or *E*_p,c_ with pH occurs over the pH range of relevance, as this is where the greatest sensitivity is offered, *i.e.* where 2e^−^2H^+^ occurs and the peak potential shifts −59.2 mV per pH unit (298.15 K). This means working with quinone molecules with p*K*_a,3_ and p*K*_a,5_ values within relevant ranges.^52^ For the model, we again use p*K*_a_ values of 0 for *Q* → *Q*H^+^ (p*K*_a,3_) and 10 for *Q*H^−^ → *Q*H_2_ (p*K*_a,5_). Fig. 8a shows the quinone modified electrode CV response as a function of pH, modelled under the same experimental conditions as Fig. 2a, except the solution is now unbuffered.

In the pH range 4 to 11, peak splitting is observed, with up to four anodic peaks (at pH 6–8) and three cathodic peaks (at pH 5) in a single scan. In the unbuffered system the voltammetric measurement perturbs the local pH, such that the local and bulk H^+^ concentrations are no longer equal, with [H^+^]_surf_ changing as the reaction progresses (eqn (19)). The peak splitting is indicative of a build-up of different quinone species, due to the proton reactions becoming rate limiting, and is especially prevalent in the pH range 5–9. A lack of available protons at the electrode surface prevents the more reduced quinone species (*e.g. Q*H_2_) from forming, see SI-14, and the mechanism shifts to *Q* → *Q*˙^−^ → *Q*^2−^ (for pH ≥ 10). For these simulations the self-ionisation of water reaction (eqn (20)) must also be considered as a source (and sink) of protons, providing minimal buffering to an otherwise unbuffered solution. A comparison of results with and without consideration of the self-ionisation of water is detailed in SI-15.

It has been experimentally suggested that lowering Γ_tot_ can move the unbuffered pH response towards that expected by buffered theory.^29^ The effect of lowering Γ_tot_ by over three orders of magnitude to 1 × 10^−13^ mol cm^−2^ on the CV response is shown in Fig. 8b for solution pH 0–14. At this very low coverage, the CV response in unbuffered solution now reflects CV scans observed in buffered solution, albeit with smaller peak currents due to the lower number of quinone groups on the electrode surface. Fig. 8c shows how [H^+^]_surf_ changes over the course of the CV scan for Γ_tot_ = 2.8 × 10^−10^, 1 × 10^−11^, 1 × 10^−12^, and 1 × 10^−13^ mol cm^−2^, for pH 5 (0.01 mM) which is the centred pH value between p*K*_a,3_ and p*K*_a,5_. As Γ_tot_ decreases, [H^+^]_surf_ is perturbed less compared to the bulk H^+^ concentration of 0.01 mM, with no perturbation predicted at Γ_tot_ = 1 × 10^−13^ mol cm^−2^. Additional plots of [H^+^]_surf_*versus* time in the pH range 0–14 are given in Fig. S16, SI-16. The quinone–quinone spacing increases from 0.8 nm to ∼41 nm (∼50 times increase), when decreasing Γ_tot_ from monolayer coverage (2.8 × 10^−10^ mol cm^−2^) to 1 × 10^−13^ mol cm^−2^.

Fig. 8d shows plots of *E*_p_*versus* pH for Γ_tot_ values of 2.8 × 10^−10^, 1 × 10^−11^, 1 × 10^−12^, and 1 × 10^−13^ mol cm^−2^. A larger range of Γ_tot_ (from 2.8 × 10^−10^ – 1 × 10^−14^ mol cm^−2^) is plotted in Fig. S17, SI-17. Interestingly, while Fig. 8c demonstrated that a Γ_tot_ of as low as 1 × 10^−13^ mol cm^−2^ was required for no measurable perturbation of [H^+^]_surf_, this surface coverage is not necessary to obtain a linear, Nernstian calibration, when plotting *E*_p_*versus* pH. Instead, a surface coverage an order of magnitude higher, Γ_tot_ = 1 × 10^−12^ mol cm^−2^ (∼12 nm quinone-quinone spacing) suffices, providing a linear slope (*R*^2^ = 0.99999) of −58.9 mV pH^−1^ between pH 0–10. This suggest some perturbation of [H^+^]_surf_ is tolerable. As Γ_tot_ increases further a deviation from the expected linear Nernstian behaviour is observed in the pH range 6–8, similar to that observed experimentally.^29^ At larger Γ_tot_ values the response deviates from linearity rapidly at pH > 5 as [H^+^]_surf_ drops to 0 during quinone reduction (yellow line in Fig. 8c).

As [H^+^]_surf_ is key in voltammetric pH measurements it is important to also consider the timescale of the reaction in relation to the flux of H^+^ towards/away from the surface during PCET in unbuffered solutions. The effects of increasing *ν* from 0.1 to 100 V s^−1^ on a monolayer quinone electrode are displayed in Fig. S18a, SI-18, where an increased Δ*E*_p_ and less Gaussian shaped peaks are observed. Note that non-faradaic contributions are not modelled here.^36^ The effect of lowering *ν* to 1 × 10^−6^ V s^−1^ is shown in Fig. S18b where a similar improvement to the unbuffered measurement is demonstrated as shown by lowering Γ_tot_. Natural convection is not included in the model. However, this is not a realistic solution as scanning this slowly takes 26 × 10^6^ s (301 days)! Decreasing *ν* significantly increases the time available for H^+^ to diffuse to the electrode surface, [H^+^]_surf_ gets depleted more slowly and the local pH reflects the bulk pH. Conversely, increasing *ν* means that there is less time for H^+^ to diffuse to the electrode surface, and [H^+^]_surf_ depletes faster and remains depleted for longer times.

## 4. Conclusion

A general finite element model has been developed that predicts the voltammetric response of surface bound redox molecules undergoing a PCET reaction, by explicitly coupling the surface reaction mechanism to mass transport of protons in an electrolyte solution. The model facilitates comparison of the simulated response to experimental data, thus enabling estimation of intrinsic parameters *e.g. k*^0^_e,*n*_, p*K*_a,*n*_, and *E*^0^_*n*_ values that would be difficult to measure directly. The model treats all proton and electron transfer steps individually, meaning the input parameters and measurement timescale determine whether the mechanism is concerted or stepwise. Whilst the focus of the work was on surface confined quinone 2e^−^2H^+^ PCET voltammetry (nine membered scheme of squares) the model (which is also provided for use) could be extended more generally to any PCET reaction.

For surface bound redox species undergoing electron transfer, the Laviron or trumpet plot method is widely used to determine experimentally apparent electron transfer rate constants, *k*_expt_, from CVs recorded at different scan rates. Literature has seen its extension to PCET reactions. The validity of this extension was assessed *via* comparison of *k*_expt_ values extracted from simulated Laviron CVs, with those determined analytically *via* expressions derived for surface bound PCET reactions. Through simulations it was shown that for a 1e^−^1H^+^ reaction both apparent ET rate constants were in agreement when very high rates of (de)protonation were used, typical of aqueous solutions. In contrast, agreement was not seen for the 2e^−^2H^+^ reaction, where the wider spread of p*K*_a_ values within the system allowed for slower rates of protonation over the same pH range, even though the rates of deprotonation were high. Hence care must be taken in quantitative interpretation of *k*_expt_ values obtained from Laviron plots for PCET schemes larger than 1e^−^1H^+^, especially as this method was only ever intended for use with single step electron transfer reactions. Consideration of non-voltammetric methods for measuring kinetics in PCET systems is also important.^53^

In addition to extracting experimental parameters, it was also demonstrated how the model could be used to optimise and guide the design of experimental systems, with a focus on quinone functionalised voltammetric pH sensors for use in unbuffered solutions. Simulations were used to show that in order to minimise depletion of protons at the electrode surface during voltammetry in unbuffered solutions, the monolayer surface coverage of quinones needed to be reduced by ∼ two orders of magnitude. In this way the model offered practical guidance to maintaining a 2e^−^2H^+^ Nernstian slope, across the appropriate pH range, for PCET-based voltammetric pH sensors in unbuffered solutions.

Further model refinements could include electrostatic effects between charged head groups,^54^ the impact of an electrical double layer,^36^ inclusion of a potential dependent transfer coefficient,^55^ along with treatment of the active species as finite-sized objects as opposed to point charges. Translation from a 1D to 2D model would also allow for surfaces other than an infinite homogeneously active surface to be modelled. This would also enable information to be obtained on how quinone molecule spacing in sub-monolayer surface coverages impacts the diffusion profile of protons during PCET voltammetry.

## Author contributions

Nafiz B Biswas: writing – original draft, writing – review and editing, software, data curation, visualisation. Katherine J Levey: writing – review and editing, conceptualisation, software, validation. Tania L Read: writing – review and editing, supervision. Julie V Macpherson: writing – review and editing, conceptualisation, supervision, funding acquisition.

## Conflicts of interest

There are no conflicts of interest.

## Supplementary Material

CP-028-D6CP01907B-s001

CP-028-D6CP01907B-s002

## Data Availability

Raw data is found in https://wrap.warwick.ac.uk/202496/. Supplementary information (SI): (1) model details; (2) electron transfer equations; (3) proton transfer equations; (4) buffered model comparison with Laviron; (5) *E*_p_*vs.* pH slopes in buffered solution; (6) surface concentrations during CV scan; (7) *E*_r,1_ and *E*_r,2_ equations; (8) 
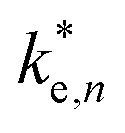
 plots for various *k*^0^_e,*n*_ Values; (9) 1e^−^1H^+^ model; (10) 1e^−^1H^+^ Laviron plot data; (11) 1e^−^1H^+^ Laviron plot data for slow proton transfer rates; (12) surface concentrations during different timescales; (13) Laviron plot data tables; (14) PCET mechanism with limited H^+^; (15) self-ionisation of water reaction; (16) [H^+^] at the electrode surface over time at varying Γ_tot_ and pH values; (17) *E*_p_*vs.* pH for a range of Γ_tot_; (18) effect of scan rate on the unbuffered PCET reaction. A separate COMSOL model report document is also available. See DOI: https://doi.org/10.1039/d6cp01907b.
