# Supplementary material for: Finite element modelling of surface-bound quinone proton coupled electron transfer voltammetry
Source: Phys Chem Chem Phys. 2026 Aug 3;28(34):20742–54. doi: 10.1039/d6cp01907b (PMC13473934; doi:10.1039/d6cp01907b)
Supplement: Supplementary file 1 [file CP-028-D6CP01907B-s001.pdf]

Supplementary Information (SI) for Physical Chemistry Chemical Physics.
This journal is © the Owner Societies 2026

Supplementary Information

Finite Element Modelling of Surface-Bound Quinone Proton Coupled Electron Transfer Voltammetry

Nafiz B. Biswas[1,2], Katherine J. Levey[1,3,*], Tania L. Read[1], Julie V. Macpherson[1,*]

[1]Department of Chemistry, University of Warwick, Coventry, CV4 7AL

[2]Centre for Diamond Science and Technology, University of Warwick, Coventry, CV4 7AL

[3]Leiden Institute of Chemistry, Leiden University, Einsteinweg 55, 2333 CC Leiden, The Netherlands

Corresponding authors: *katherine.j.levey@warwick.ac.uk and *j.macpherson@warwick.ac.uk

Contents

SI 1: Model Details 3
- S1.1: Model Geometry 3
- SI-1.2: Cyclic Voltammogram Simulation 3
- SI-1.3: General System Parameters 4
- SI-1.4: Diffusion of Ions in Solution 5
- SI-1.5: Mesh Test 5
- SI-1.6: Convergence Test 6
- SI-1.7: Model Parameter Reference Lists 7

SI-2: Electron Transfer Equations 10
SI-3: Proton Transfer Equations 11
SI-4: Buffered Model Comparison With Laviron 12
SI-5: E_p *vs* pH slopes in Buffered Solution 13
SI-6: Surface Concentrations During CV Scan 14
SI-7: $E_{r,1}$ and $E_{r,2}$ Equations 16
SI-8: $k^*_{e,n}$ Plots for Various $k_{e,n}^0$ Values 17
SI-9: $1e^-1H^+$ Model 18
SI-10: $1e^-1H^+$ Laviron Plot Data 19
SI-11: $1e^-1H^+$ Laviron Plot Data for Slow Proton Transfer Rates 20
SI-12: Surface Concentrations During Different Timescales 22
SI-13: Laviron Plot Data Tables 23
SI-14: PCET Mechanism With Limited H^+ 25

SI-15: Self-Ionisation of Water Reaction 26
SI-16: $[H^+]$ at the Electrode Surface Over Time at Varying Γ_{tot} and pH Values 27
SI-17: E_p *vs* pH for a Range of Γ_{tot} 28
SI-18: Effect of Scan Rate on the Unbuffered PCET Reaction 29
References 30

SI 1: Model Details

S1.1: Model Geometry

This model uses a simple 1 dimensional (1D) geometry, ***Figure S1***, to model a macrodisk electrode (infinite plane) covered uniformly by surface bound quinone molecules with a total quinone surface coverage (Γ_{tot}, mol cm^{-2}) that can be varied. The model consists of 2 boundary points; boundary 1 is the electrode surface (where the quinones are bound), and boundary 2 represents the bulk solution. The boundary points are separated by a single 1D domain (1 cm long), through which protons (H^+) in solution diffuse. The quinones are treated as points on the surface, where each point is a single active site.

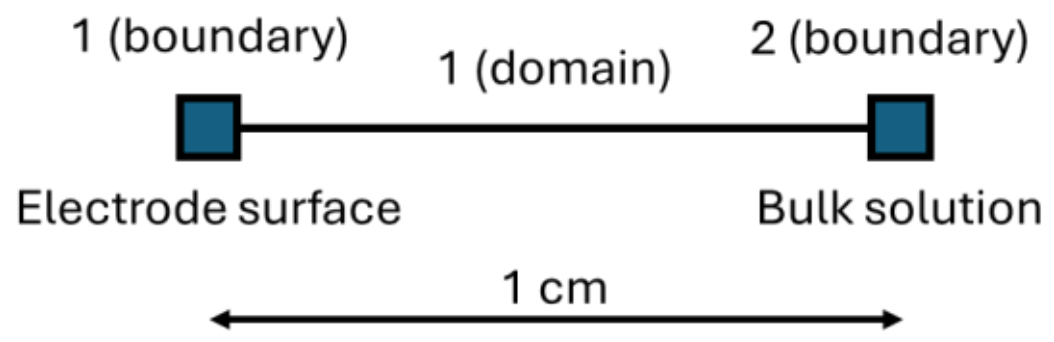

Figure S1. 1D geometry used for the numerical model.

SI-1.2: Cyclic Voltammogram Simulation

The cyclic voltammogram (CV) is simulated using a piecewise function using ***Equations S1*** and ***S2***. The applied voltage (E) first decreases from an initial potential (E_{in}) until a switching potential (E_s) is reached, where v is the scan rate, and t is the time. In this case, a reductive scan occurs first (e.g. converting M to V, see ***Figure 1*** in main text), before scanning oxidatively (e.g. converting V back to M).

$$E = E_{\mathrm{in}} - vt \qquad (S1)$$

$$E = E_{\mathrm{s}} + vt \qquad (S2)$$

SI-1.3: General System Parameters

The required system parameters and their expressions can be found in ***Table S1***.

Table S1. Table containing the system parameters required for this model, including the initial values used (where applicable) and a Parameter description.

Parameter Name	Symbol	Expression [Units]	Description
H^+ diffusion coefficient	D_{H+}	5×10^{-5} [cm^2 s^{-1}],[1]	Diffusion coefficient of H^+ in solution (aqueous)
Hydroxide (OH^-) diffusion coefficient	D_{OH-}	5.27×10^{-5} [cm^2 s^{-1}],[2]	Diffusion coefficient of OH^- in solution (aqueous)
Bulk pH	pH	7 (e.g.)	The pH of the electrolyte solution (value changed by the user as necessary)
Bulk H^+ concentration	$[H^+]_{bulk}$	10^{-pH} [M]	The initial concentration of H^+ at all boundaries/domains
Bulk pOH	pOH	$14 - \mathrm{pH}$	The pOH of the electrolyte solution
Bulk OH^- concentration	$[OH^-]_{bulk}$	$10^{-\mathrm{pOH}}$ [M]	The initial concentration of OH^- at all boundaries/domains
Number of e^- transferred	n	1	Number of e^- transferred for each e^- transfer reaction (e.g. $M \rightarrow N$)
Temperature	T	298.15 [K]	Temperature of system
Butler-Volmer constant	f	$\frac{F}{RT}$ [V^{-1}]	Where F is Faraday's constant, and R is the gas constant
Total Quinone site density	Γ_{tot}	2.8×10^{-10} [mol cm^{-2}] (monolayer coverage)[3]	The total (max.) quinone site density available (value changed by the user as necessary)

SI-1.4: Diffusion of Ions in Solution

The diffusion of H^+ and OH^- through the solution (domain 1) is dictated by the their respective diffusion coefficients, and the Nernst-Planck equation (***Equation S3***, where **J** is the flux). The flux of H^+ ($[H^+]_{bulk}$) and OH^- ($[OH^-]_{bulk}$) in the bulk boundary is always 0, ***Equation S4*** and ***S5***. This ensures that the pH of the bulk remains constant. The flux of OH^- at the electrode surface ($[OH^-]_{surf}$) is also 0, as no OH^- is required for the PCET reactions, ***Equation S6***. However, the flux of H^+ at the electrode surface ($[H^+]_{surf}$) is dependent on whether a buffered or unbuffered solution is being modelled, discussed in section **3.2. Proton Reactions** in the main text. No convection or migration is modelled as the solution is assumed to be stagnant with high background electrolyte concentrations.[4]

$$\frac{\partial\left[\mathrm{H}^{+}\right]}{\partial t}+\nabla\cdot\mathbf{J}=0 \qquad (S3)$$

$$\frac{\partial\left[\mathrm{H}^{+}\right]_{\mathrm{bulk}}}{\partial t}=0 \qquad (S4)$$

$$\frac{\partial\left[\mathrm{OH}^{-}\right]_{\mathrm{bulk}}}{\partial t}=0 \qquad (S5)$$

$$\frac{\partial\left[\mathrm{OH}^{-}\right]_{\mathrm{surf}}}{\partial t}=0 \qquad (S6)$$

SI-1.5: Mesh Test

As the reaction is occurring, the major changes are all happening at the electrode surface. One of the most challenging scenarios to solve is at pH 7 in unbuffered media; this is when H^+ is perturbed the most and apparent ET rates are slowest. Therefore, to determine the maximum permissible size of the mesh at the electrode surface (without affecting the results), the size was initially varied from 1 mm to 1 nm for the unbuffered model at pH 7. As seen in ***Figure S2***, when a mesh size of 1 nm was reached, the result overlapped exactly with that of 10 nm (and by extension every size in between), before deviating at larger values. Therefore, to minimise computational requirements while maintaining accuracy, a minimum mesh size of 10 nm was used for all simulations.

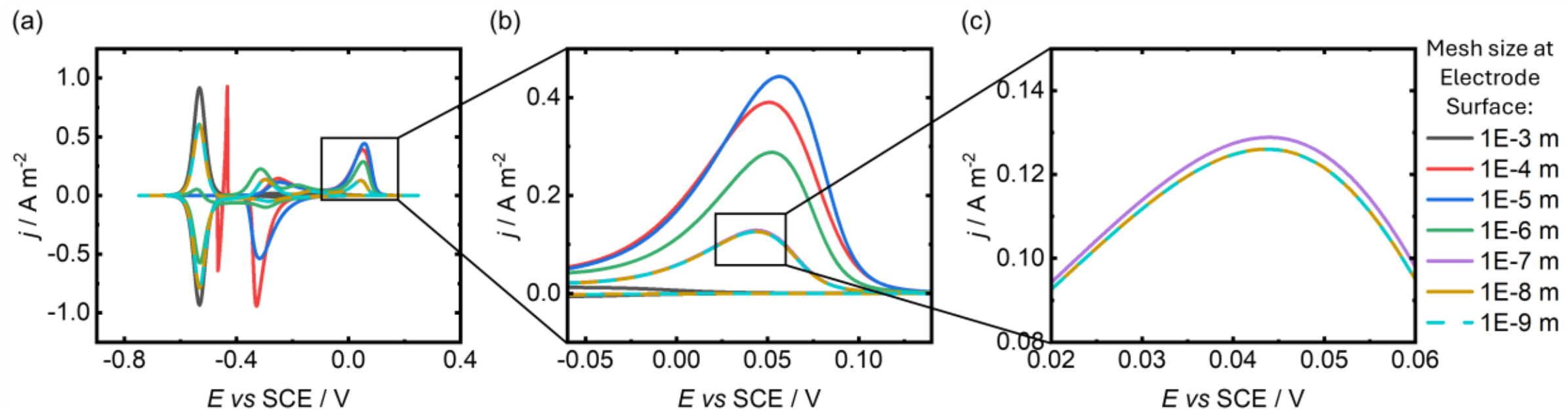

Figure S2. Mesh test where the mesh size was changed from 1 mm to 1 nm at pH 7 in unbuffered media. (a) full CV scan, (b) magnified area of (a), (c) magnified area of (b).

SI-1.6: Convergence Test

A convergence test was conducted by altering the relative tolerance of the solver. The relative tolerance refers to the maximum amount of error that is allowed between consecutive iterations of a solver. No significant changes were seen in the range 1×10^{-2} to 1×10^{-4}, (as seen in ***Figure S3***) therefore the relative tolerance was left at 1×10^{-3} (the default set by COMSOL).

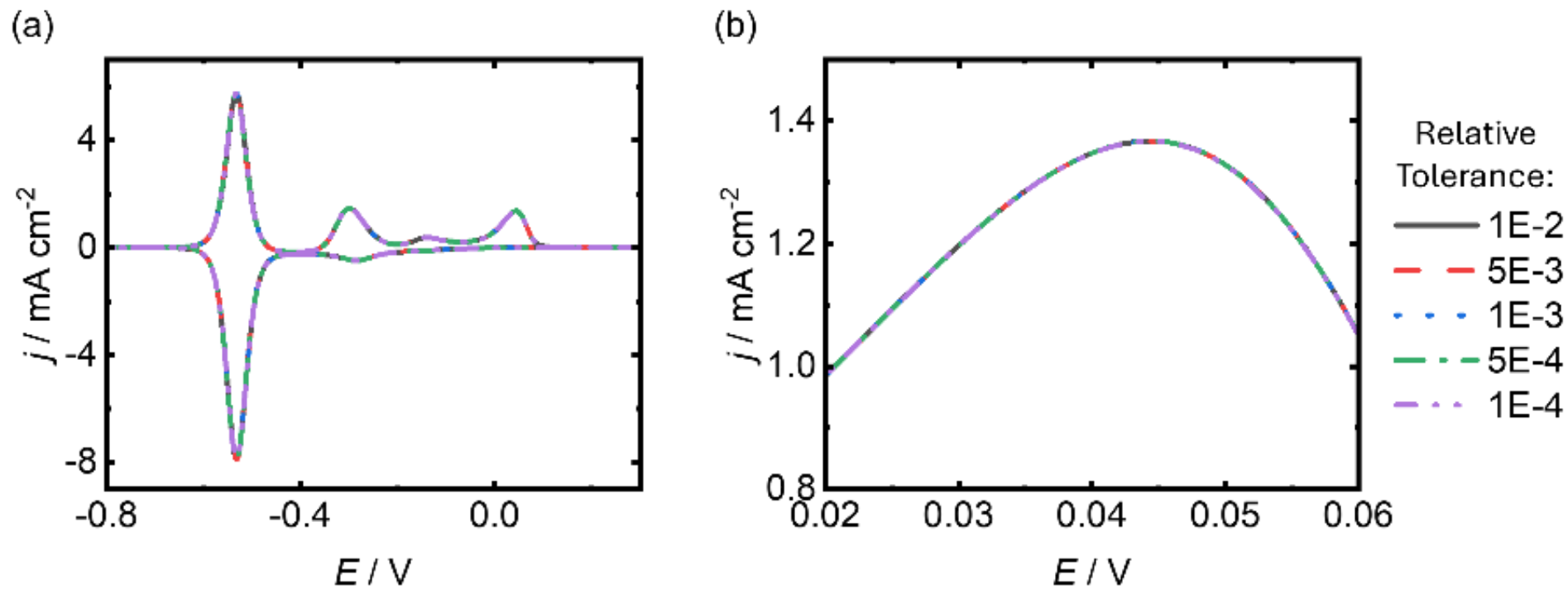

Figure S3. Convergence test where the relative tolerance was changed from 1×10^{-2} to 1×10^{-4}. (a) the full CV scan, (b) a magnified portion of the scan

SI-1.7: Model Parameter Reference Lists

Table S2. Parameters used in the model

Parameter	Value	References
p$K_{a,n}$ Values	The p$K_{a,n}$ values chosen will determine the pH window in which the $2e^-2H^+$ and $2e^-1H^+$ reaction mechanisms will occur. The most important is p$K_{a,3}$ (lower limit for $2e^-2H^+$) and p$K_{a,5}$ (upper limit for $2e^-2H^+$). For most quinone systems, setting p$K_{a,3}$ = 0 and p$K_{a,5}$ = 10 is sensible, with the other pK_a's following the SS equilibrium (*vide supra*). These are the same values used by Laviron (Figure 7 in reference 5)	5
E^0 Values	E_2^0 can be set to any arbitrary number that determines the E_p range, we use 0.25 V and $E_5^0 = E_2^0 - 0.5$ V. The remaining E^0's are calculated from the following equations: $E_1^0 = E_2^0 + \frac{2.303}{f}\left(\mathrm{p}K_{a,2} - \mathrm{p}K_{a,1}\right)$ $E_3^0 = E_2^0 - \frac{2.303}{f}\left(\mathrm{p}K_{a,4} - \mathrm{p}K_{a,3}\right)$ $E_4^0 = E_5^0 - \frac{2.303}{f}\left(\mathrm{p}K_{a,2} - \mathrm{p}K_{a,5}\right)$ $E_6^0 = E_5^0 + \frac{2.303}{f}\left(\mathrm{p}K_{a,4} - \mathrm{p}K_{a,6}\right)$	5
Γ_{tot} / mol cm^{-2}	Quinone Monolayer Coverage = 2.8×10^{-10} mol cm^{-2} for 2-mercaptohydroquinone on gold – the impact of lowering surface coverage is shown in ***Figure 8***.	3
$k_{b,Pn}$ / s^{-1}	The deprotonation reaction rate is assumed to be fast (1×10^{-9} s^{-1}). See section **3.2** in the main text for nuance. The impact of reducing $k_{b,Pn}$ is in ***Figure S11***.	6

$k_{e.n}^{0}$ / s^{-1}	Maximum of 1×10^{6} s^{-1} used in ***Figure 2***, main text.<br>Impact of reducing to 100 s^{-1} shown in ***Figures 4*** and ***5***, main text.<br>2×10^{4} s^{-1} used in ***Figures 6 – 8*** (Laviron's analytical expressions,[5] compared to experimental data obtained for BDD-Q electrodes in reference [7] to back-calculate intrinsic $k_{e,n}^{0}$ values)	5,7
Diffusion Coefficients	$D_{H+} = 5 \times 10^{-5}$ cm^2 s^{-1}	1
	$D_{OH-} = 5.27 \times 10^{-5}$ cm^2 s^{-1}	2
Self-Ionisation of Water Reaction	Concentration of water, $[H_2O] = 55.5$ mol dm^{-3}	
	Rate of dissociation of water, $k_d = 2.6 \times 10^{-5}$ [s^{-1}]	8
	Rate of recombination of water, $k_r = 1.4 \times 10^{-11}$ [dm^3 mol^{-1} s^{-1}]	9

Table S3. Examples of parameter values from literature not used in the model, and experimental results.

Parameter	Value	References
p$K_{a,n}$ Values	Hashemi – p$K_{a,n}$ values found using DFT modelling for various quinone structures	10
Γ_{tot} / mol cm^{-2}	Bisdiaminobenzoquinone dyad monolayer on gold, $\Gamma_{tot} = 1.3 \times 10^{-10}$ mol cm^{-2}	11
	5-hydroxy-3-hexanedithiol-1,4-naphthoquinone (JUG_{thio}) monolayer on gold, $\Gamma_{tot} = 2.2 \times 10^{-10}$ mol cm^{-2}	12
	2,5-Dihydroxythiophenol on Pt $\Gamma_{tot} = 5.6 \times 10^{-10}$,and on Au, $\Gamma_{tot} = 5.4 \times 10^{-10}$ mol cm^{-2}	13
$k_{expt,x}$ from Laviron Plots for $2e^-2H^+$ PCET / s^{-1}	**Surface bound Q's on BDD**, introduced through laser micromachining ($k_{expt,a}/k_{expt,c}$)<br>Buffered: pH 4 → 250/260, pH 7 → 230/73, pH 10 → 220/28 s^{-1}<br>Unbuffered: pH 4 → 250/190, pH 7 → 5.4/4.8, pH 10 → 57/14 s^{-1}	7
	**Immobilised anthraquinone in pH 7 buffer**<br>On BDD → 0.41 s^{-1}<br>On GC → 4.5 s^{-1}	14

	**Immobilised 2-amino-1,4-naphthoquinone (SAM) at pH 6.9**<br>Buffered → 200 s^{-1}<br>Unbuffered → 220 s^{-1}	15
	**Anthraquinone-2,7-disulphonic acid**:<br>pH 1.4 → 1400 s^{-1}, pH 4.2 → 340 s^{-1}	16
	**1,4-benzoquinone headgroup connected to an oligo(phenylene vinylene) (OPV) thiol** with a single, double, or triple bonded bridge (buffered)<br>Single bond: pH 1 – 12 → 0.003 – 6.1 s^{-1}<br>Double bond: pH 1 – 12 → 9 – 647 s^{-1}<br>Triple bond: pH 1 – 12 → 1.42 – 84.1 s^{-1}	17
	**1,4-benzoquinone headgroup with alkane or OPV bridges** (buffered)<br>Alkane bridge: pH 8 – 12.6 → 0.07 – 62 s^{-1}<br>OPV bridge: pH 8 – pH 12.6 → 83 – 256 s^{-1}	18
	**Aminoanthraquinone:**<br>pH 1 (0.1 M H_2SO_4) at 7.3°C, 16.1°C, 34.6°C → 25, 44, 49 s^{-1} respectively	19
	**Anthraquinone on L-shaped or straight norbornylogous bridges**:<br>pH 4.2 → < 1 s^{-1}, pH 11.4 → 3 – 35 s^{-1}	20
	**Self-assembled aminobenzoquinone monolayers:**<br>pH 1.5 – 8.5 (buffered) → 0.24 – 0.16 s^{-1}	21

SI-2: Electron Transfer Equations

All the electron transfer (ET) equations are given by ***Equations S7 – S15***.

$$\left(\frac{\partial \Gamma_{\mathrm{M}}}{\partial t}\right)_{\mathrm{ET}} = k_{b,e_3}\Gamma_{\mathrm{N}} - k_{f,e_3}\Gamma_{\mathrm{M}} \quad (S7)$$

$$\left(\frac{\partial \Gamma_{\mathrm{O}}}{\partial t}\right)_{\mathrm{ET}} = k_{b,e_2}\Gamma_{\mathrm{P}} - k_{f,e_2}\Gamma_{\mathrm{O}} \quad (S8)$$

$$\left(\frac{\partial \Gamma_{\mathrm{Q}}}{\partial t}\right)_{\mathrm{ET}} = k_{b,e_1}\Gamma_{\mathrm{R}} - k_{f,e_1}\Gamma_{\mathrm{Q}} \quad (S9)$$

$$\left(\frac{\partial \Gamma_{\mathrm{S}}}{\partial t}\right)_{\mathrm{ET}} = -\left(k_{b,e_6}\Gamma_{\mathrm{S}} - k_{f,e_6}\Gamma_{\mathrm{N}}\right) \quad (S10)$$

$$\left(\frac{\partial \Gamma_{\mathrm{T}}}{\partial t}\right)_{\mathrm{ET}} = -\left(k_{b,e_5}\Gamma_{\mathrm{T}} - k_{f,e_5}\Gamma_{\mathrm{P}}\right) \quad (S11)$$

$$\left(\frac{\partial \Gamma_{\mathrm{V}}}{\partial t}\right)_{\mathrm{ET}} = -\left(k_{b,e_4}\Gamma_{\mathrm{V}} - k_{f,e_4}\Gamma_{\mathrm{R}}\right) \quad (S12)$$

$$\left(\frac{\partial \Gamma_{\mathrm{N}}}{\partial t}\right)_{\mathrm{ET}} = -\left(\left(\frac{\partial \Gamma_{\mathrm{M}}}{\partial t}\right)_{\mathrm{ET}} + \left(\frac{\partial \Gamma_{\mathrm{S}}}{\partial t}\right)_{\mathrm{ET}}\right) \quad (S13)$$

$$\left(\frac{\partial \Gamma_{\mathrm{P}}}{\partial t}\right)_{\mathrm{ET}} = -\left(\left(\frac{\partial \Gamma_{\mathrm{O}}}{\partial t}\right)_{\mathrm{ET}} + \left(\frac{\partial \Gamma_{\mathrm{T}}}{\partial t}\right)_{\mathrm{ET}}\right) \quad (S14)$$

$$\left(\frac{\partial \Gamma_{\mathrm{R}}}{\partial t}\right)_{\mathrm{ET}} = -\left(\left(\frac{\partial \Gamma_{\mathrm{Q}}}{\partial t}\right)_{\mathrm{ET}} + \left(\frac{\partial \Gamma_{\mathrm{V}}}{\partial t}\right)_{\mathrm{ET}}\right) \quad (S15)$$

SI-3: Proton Transfer Equations

All the proton transfer (PT) equations are given by ***Equations S16 – S24***.

$$\left(\frac{\partial \Gamma_{\mathrm{M}}}{\partial t}\right)_{\mathrm{PT}} = k_{b,p_3}\Gamma_{\mathrm{O}} - k_{f,p_3}\Gamma_{\mathrm{M}} \qquad (S16)$$

$$\left(\frac{\partial \Gamma_{\mathrm{N}}}{\partial t}\right)_{\mathrm{PT}} = k_{b,p_4}\Gamma_{\mathrm{P}} - k_{f,p_4}\Gamma_{\mathrm{N}} \qquad (S17)$$

$$\left(\frac{\partial \Gamma_{\mathrm{S}}}{\partial t}\right)_{\mathrm{PT}} = k_{b,p_6}\Gamma_{\mathrm{T}} - k_{f,p_6}\Gamma_{\mathrm{S}} \qquad (S18)$$

$$\left(\frac{\partial \Gamma_{\mathrm{Q}}}{\partial t}\right)_{\mathrm{PT}} = -\left(k_{b,p_1}\Gamma_{\mathrm{Q}} - k_{f,p_1}\Gamma_{\mathrm{O}}\right) \qquad (S19)$$

$$\left(\frac{\partial \Gamma_{\mathrm{R}}}{\partial t}\right)_{\mathrm{PT}} = -\left(k_{b,p_2}\Gamma_{\mathrm{R}} - k_{f,p_2}\Gamma_{\mathrm{P}}\right) \qquad (S20)$$

$$\left(\frac{\partial \Gamma_{\mathrm{V}}}{\partial t}\right)_{\mathrm{PT}} = -\left(k_{b,p_5}\Gamma_{\mathrm{V}} - k_{f,p_5}\Gamma_{T}\right) \qquad (S21)$$

$$\left(\frac{\partial \Gamma_{\mathrm{O}}}{\partial t}\right)_{\mathrm{PT}} = -\left(\left(\frac{\partial \Gamma_{\mathrm{M}}}{\partial t}\right)_{\mathrm{PT}} + \left(\frac{\partial \Gamma_{\mathrm{Q}}}{\partial t}\right)_{\mathrm{PT}}\right) \qquad (S22)$$

$$\left(\frac{\partial \Gamma_{\mathrm{P}}}{\partial t}\right)_{\mathrm{PT}} = -\left(\left(\frac{\partial \Gamma_{\mathrm{N}}}{\partial t}\right)_{\mathrm{PT}} + \left(\frac{\partial \Gamma_{\mathrm{R}}}{\partial t}\right)_{\mathrm{PT}}\right) \qquad (S23)$$

$$\left(\frac{\partial \Gamma_{\mathrm{T}}}{\partial t}\right)_{\mathrm{PT}} = -\left(\left(\frac{\partial \Gamma_{\mathrm{S}}}{\partial t}\right)_{\mathrm{PT}} + \left(\frac{\partial \Gamma_{\mathrm{V}}}{\partial t}\right)_{\mathrm{PT}}\right) \qquad (S24)$$

SI-4: Buffered Model Comparison With Laviron

In ***Figure S4*** we can see a comparison of the slope obtained from our model against the slope from Laviron data in the $2e^{-}2H^{+}$ region (data points extracted from Fig. 7 in reference 5 using https://plotdigitizer.com/app). Both slopes and intercept are in very good agreement.

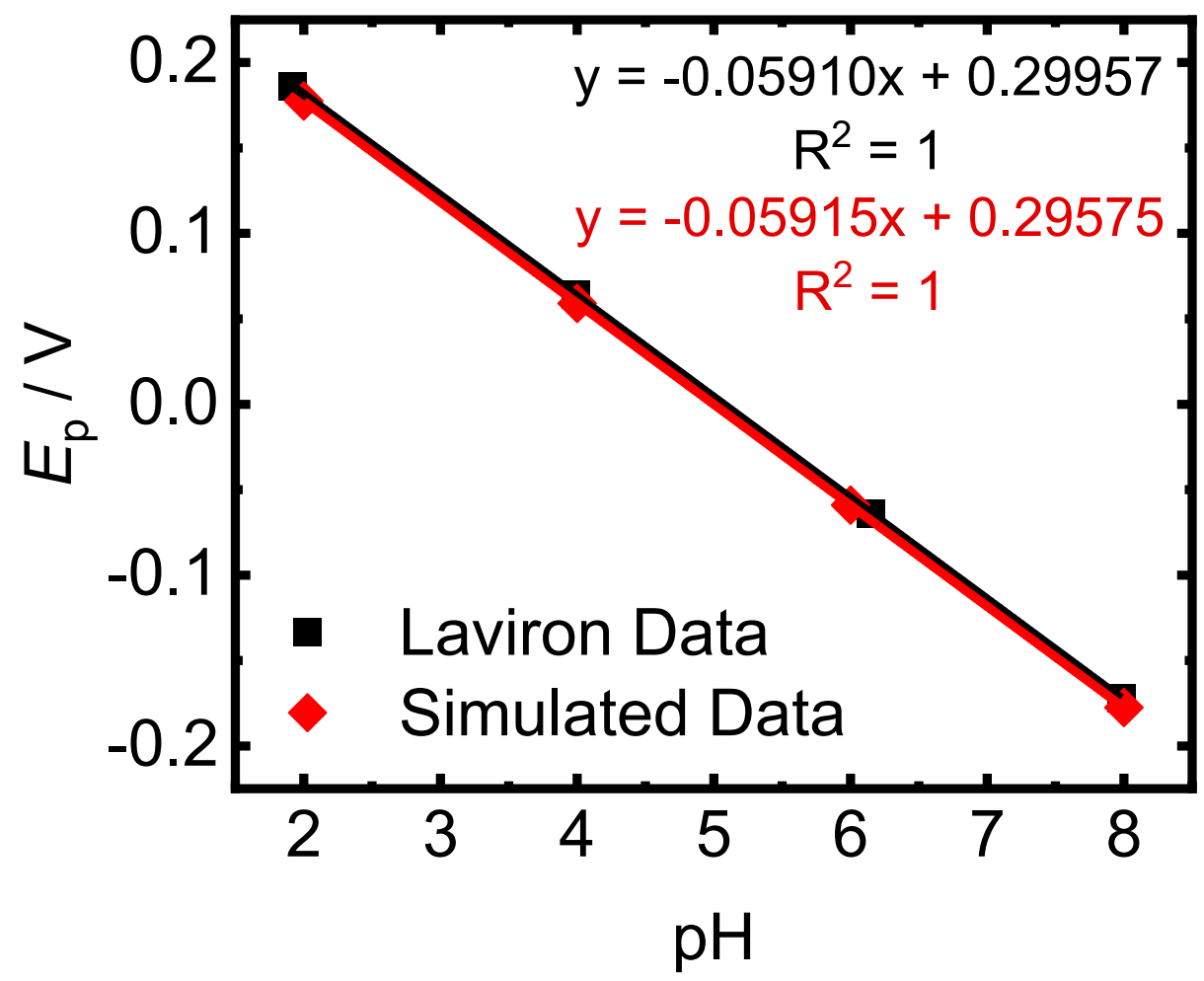

Figure S4. Linear plots of E_p *vs* pH for the pH range 2 – 8, where the $2e^{-}2H^{+}$ reaction occurs, for the data extracted from reference 5 and the simulated data in ***Figure 2a*** in the main text.

SI-5: E_p *vs* pH slopes in Buffered Solution

E_p values were calculated by averaging the potential at the current maximum for both the anodic ($E_{p,a}$) and cathodic ($E_{p,c}$) peak potential. For the buffered reaction no difference in $E_{p,a}$ and $E_{p,c}$ is observed. By plotting E_p against pH, the slopes for the $2e^-2H^+$, $2e^-1H^+$, and $2e^-$ reaction mechanisms can be obtained and compared against theory. To get an accurate slope, the data points at pH values equal to $pK_{a,1}$ (-8), $pK_{a,3}$ (0), $pK_{a,5}$ (10), and $pK_{a,6}$ (18) were not included in the slope calculations, as these values are the point at which the mechanism changes, and both $2e^-1H^+$ and $2e^-2H^+$ can occur. Between pH 2 – 8 ($pK_{a,3}$ – $pK_{a,5}$), where the $2e^-2H^+$ reaction occurs, the slope is -59.15 mV / pH unit with $R^2 = 1$ (***Figure S5a***). For the $2e^-1H^+$ reaction which occurs between pH -6 – 2 ($pK_{a,1}$ – $pK_{a,3}$) and pH 12 – 16 ($pK_{a,5}$ – $pK_{a,6}$), slopes of -29.50 mV / pH unit ($R^2 = 1$) are calculated (***Figure S5b*** and ***c***). For the $2e^-$ reaction, where pH < -8 (< $pK_{a,1}$) or pH >18 (> $pK_{a,6}$), slopes of 0 mv / pH unit are determined from unchanging E_p values (at E_p = +0.533 and E_p = -0.533 V respectively).

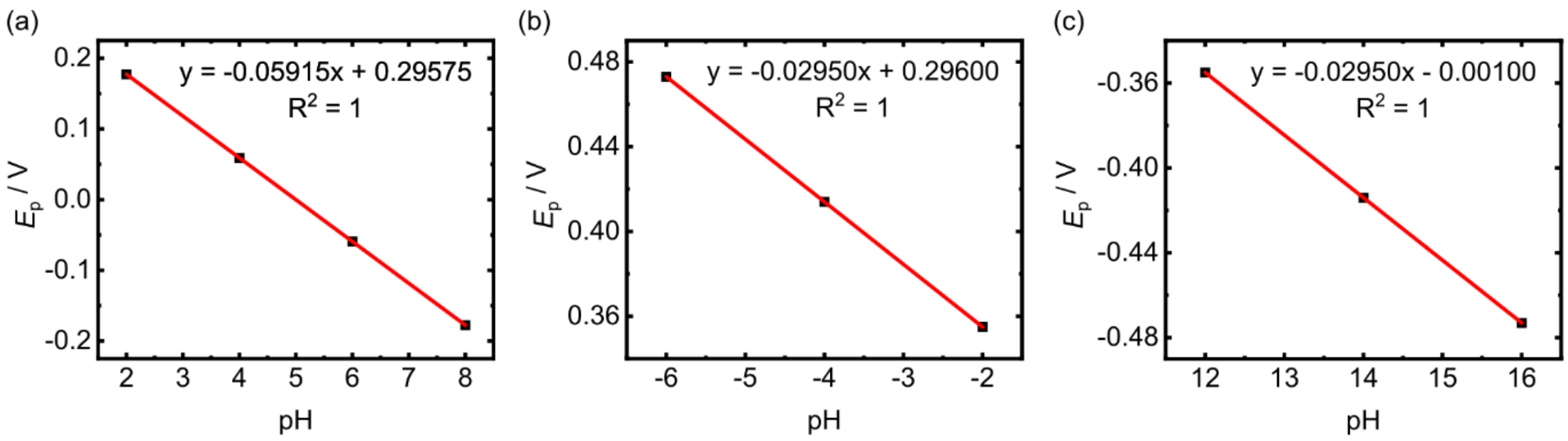

Figure S5. Linear plots of E_p *vs* pH for pH ranges (a) 2 to 8 where the $2e^-2H^+$ reaction occurs, (b) -6 to -2 where the $2e^-1H^+$ reaction occurs, and (c) 12 – 16 where the $2e^-1H^+$ reaction also occurs.

SI-6: Surface Concentrations During CV Scan

The surface coverages, Γ_i, for all quinone species (M to V) for different pH values are displayed in ***Figure S6*** (a) pH = $pK_{a,1}$ = -8, (b) pH = $pK_{a,3}$ = 0, (c) pH = $pK_{a,5}$ =10, and (d) pH = $pK_{a,6}$ = 18) and ***Figure S7*** (a) pH = 2, (b) pH = 4, (c) pH = 6, (d) pH = 8, (e) pH = 12, and (f) pH = 14). These figures give us insight into the reaction pathway of the quinone molecule during the course of the CV.

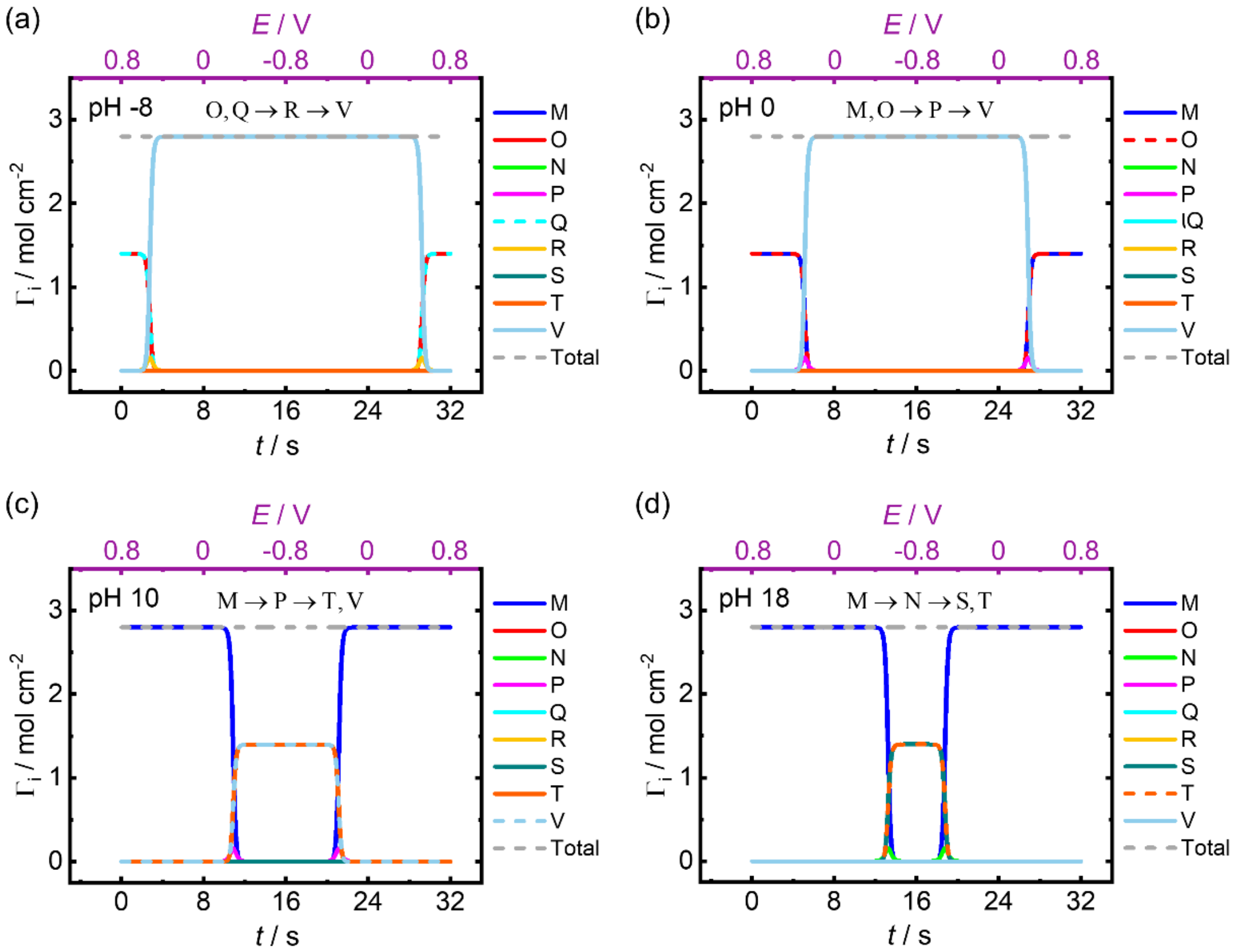

Figure S6. The change in Γ_i during the simulated CV in solution of (a) pH -8, (b) pH 0, (c) pH 10, and (d) pH 18 at 0.1 V s^{-1}.

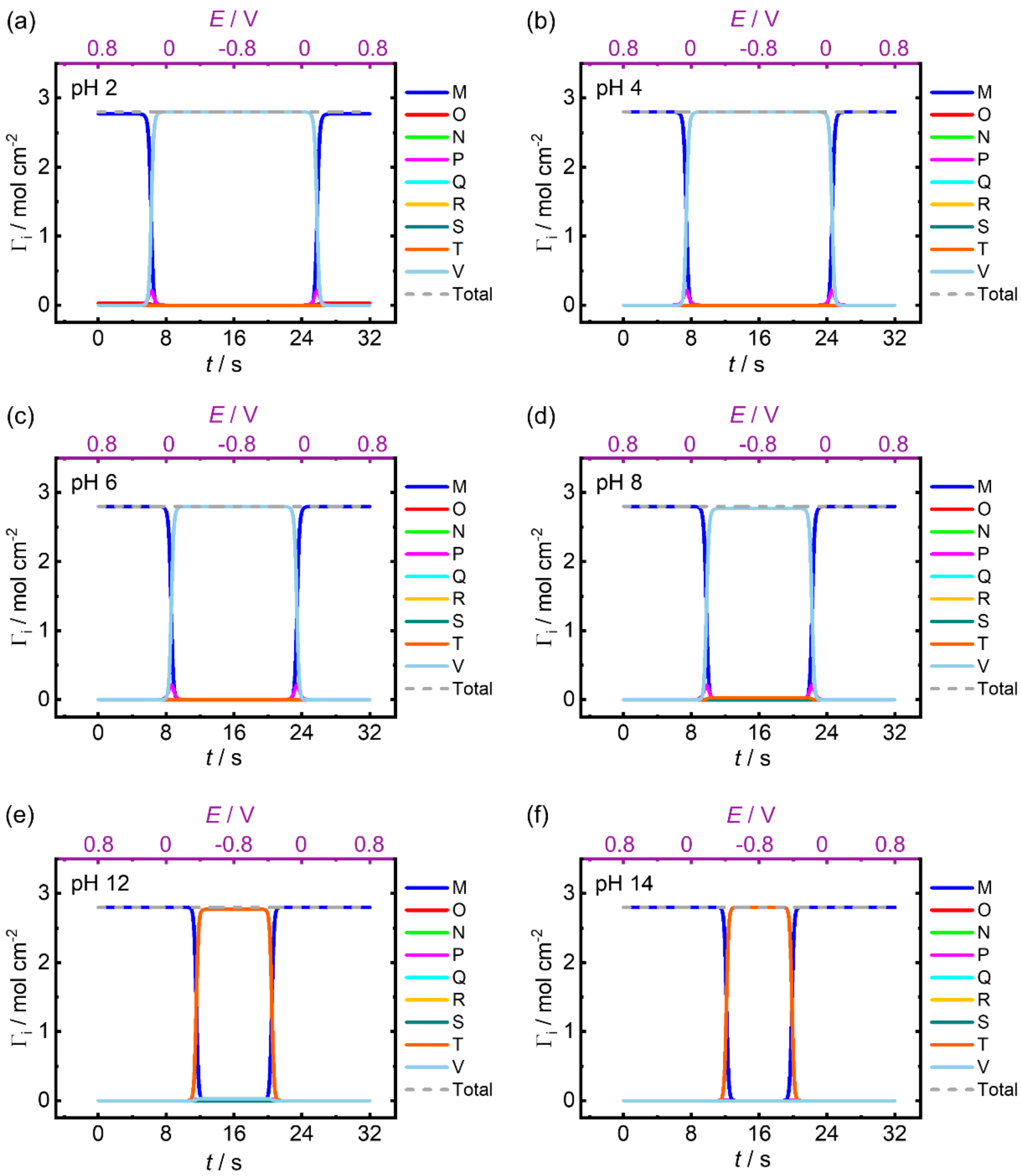

***Figure S7**.* The change in Γ_i during the simulated CV in solution of (a) pH 2, (b) pH 4, (c) pH 6, (d) pH 8, (e) pH 12, and (f) pH 14 at 0.1 V s^{-1}.

SI-7: $E_{r,1}$ and $E_{r,2}$ Equations

Equations S25 and ***S26*** determine how $E_{r,1}$ and $E_{r,2}$ can be calculated from E_2^0 and E_5^0 respectively. From this, a different calculation for ρ_s, the square root of the constant for semiquinone formation,[22] can be found (***Equation S27***). This calculation is mathematically similar to ***Equation 25*** in the main text. Much like the apparent ET rate constants $k^*_{e,n}$, $E_{r,1}$ and $E_{r,2}$ are the redox potential values of the two separate steps seen in the simplified scheme of squares mechanism, and can be considered as a condensed average of $E_1^0 - E_3^0$ (for $E_{r,1}$) and $E_4^0 - E_6^0$ (for $E_{r,2}$).

$$\exp\left[\left(\frac{F}{RT}\right)\left(E_{r,1} - E_2^0\right)\right] = \left(K_2 + 1 + \frac{1}{K_4}\right)\left(K_1 + 1 + \frac{1}{K_3}\right)^{-1} \quad (S25)$$

$$\exp\left[\left(\frac{F}{RT}\right)\left(E_{r,2} - E_5^0\right)\right] = \left(K_5 + 1 + \frac{1}{K_6}\right)\left(K_2 + 1 + \frac{1}{K_4}\right)^{-1} \quad (S26)$$

$$\therefore \rho_s = \exp\left[\left(\frac{F}{2RT}\right)\left(E_{r,1} - E_{r,2}\right)\right] \quad (S27)$$

(Page 30 from Laviron, reference 5)

SI-8: $k^*_{e,n}$ Plots for Various $k_{e,n}^0$ Values

Figure S8, shows plots of the analytically derived $k^*_{e,n}$ values versus pH, for $k_{e,n}^0$ values of (a) 500, (b) 1000, and (c) 1×10^6 s^{-1} are presented. Each $k^*_{e,n}$ versus pH plot has the same shape, and the values scale linearly depending on the intrinsic $k_{e,n}^0$ values used.

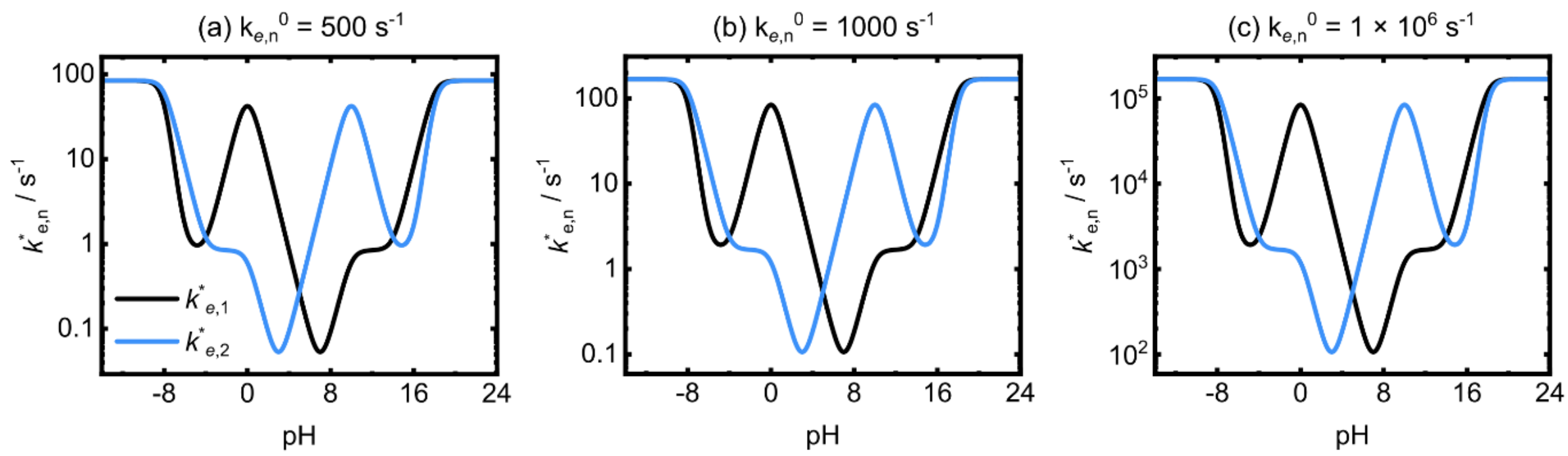

Figure S8. The $k^*_{e,n}$ plots for (a) $k_{e,n}^0 = 500$ s^{-1}, (a) $k_{e,n}^0 = 1000$ s^{-1}, (c) $k_{e,n}^0 = 1 \times 10^6$ s^{-1}.

SI-9: $1e^{-}1H^{+}$ Model

A $1e^{-}1H^{+}$ PCET reaction was modelled to allow comparison to the $2e^{-}2H^{+}$ model. For this model, values of $pK_{a1} = 4$, $pK_{a2} = 10$, $E_1^0 = 0.18$ V, $E_2^0 = -E_1^0$, and $k_{e,n}^0 = 2 \times 10^4$ s^{-1} were used. Only one analytically derived apparent ET rate constant, k^*_e, found using ***Equation S28***, is determined for the $1e^{-}1H^{+}$ PCET reaction.

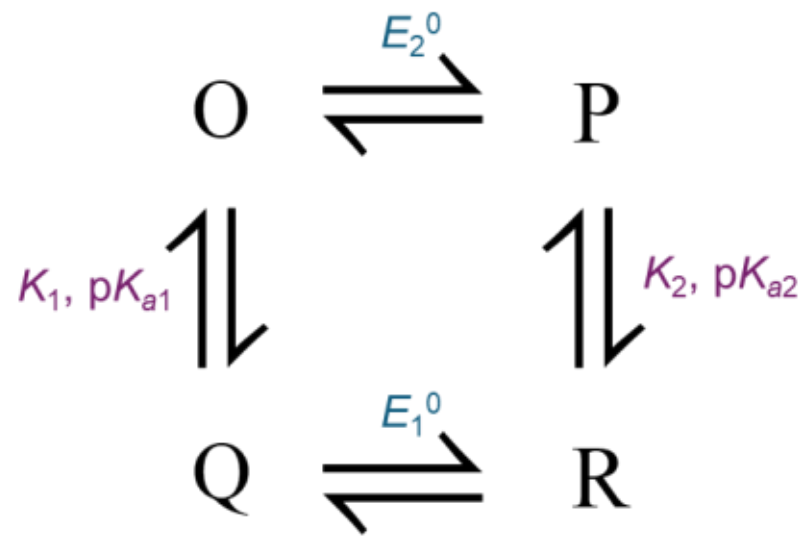

Figure S9. The scheme of squares model for a $1e^{-}1H^{+}$ PCET reaction.

$$k^*_e = k_{e,2}^0\left[1+\left(\frac{k_{e,1}^0}{k_{e,2}^0}\right)\left(K_{a1}K_{a2}\right)^{-\frac{1}{2}}\left[H^+\right]\right]\left[1+\frac{\left[H^+\right]}{K_{a1}}\right]^{-\frac{1}{2}}\left[1+\frac{\left[H^+\right]}{K_{a2}}\right]^{-\frac{1}{2}} \quad (\textbf{\textit{S28}})$$

It is important to note that, for the $1e^{-}1H^{+}$ system there is only a single k^*_e value as the four membered "scheme of squares" model is condensed into a single ET step.

SI-10: 1e^-1H^+ Laviron Plot Data

Figure S10 shows simulated (a) CV scans for the 1e^-1H^+ mechanism for buffered pH solutions of (i) 4, (ii) 7 and (iii) 10, over the scan rate range 0.1 to 1 × 10^6 V s^{-1}. From these the Laviron plots in (b) are obtained. A single peak is observed for both anodic and cathodic scans with only minimal peak broadening occurring at fast scan rates (v > 1 × 10^4 V s^{-1}).

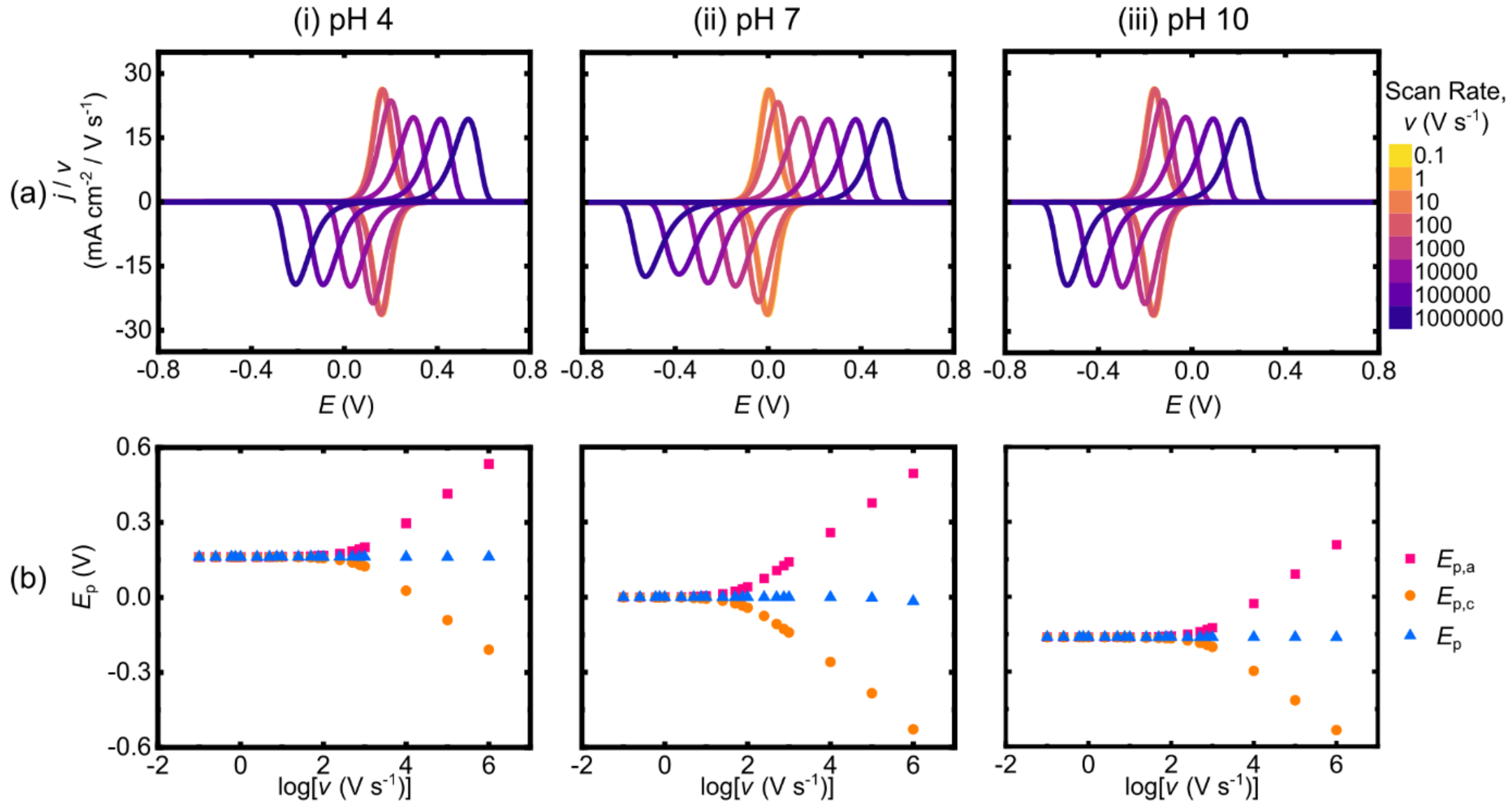

Figure S10. (a) Simulated CV scans with j normalised by v, and (b) Laviron plots at varying v (0.1 – 1 × 10^6 V s^{-1}) for buffered (i) pH 4, (ii) pH 7, and (iii) pH 10. $k_{e,n}{}^0 = 2 \times 10^4$ s^{-1}, k_{b,p_n} = 1 × 10^9 s^{-1}, and $\Gamma_{tot} = 2.8 \times 10^{-10}$ mol cm^{-2} (monolayer coverage)

SI-11: $1e^{-}1H^{+}$ Laviron Plot Data for Slow Proton Transfer Rates

Figure S11 shows simulated (a) CV scans for the $1e^{-}1H^{+}$ mechanism for buffered pH solutions of (i) 4, (ii) 7 and (iii) 10, over the scan rate range 0.1 to 1 × 10^6 V s^{-1} for slow deprotonation rate constants (k_{b,p_n} = 1 × 10^5 s^{-1}). From these the Laviron plots in (b) are obtained. The slower k_{b,p_n} now causes multiple peaks in a single scan direction to be observed leading to difficulties when peak picking to find E_p values.

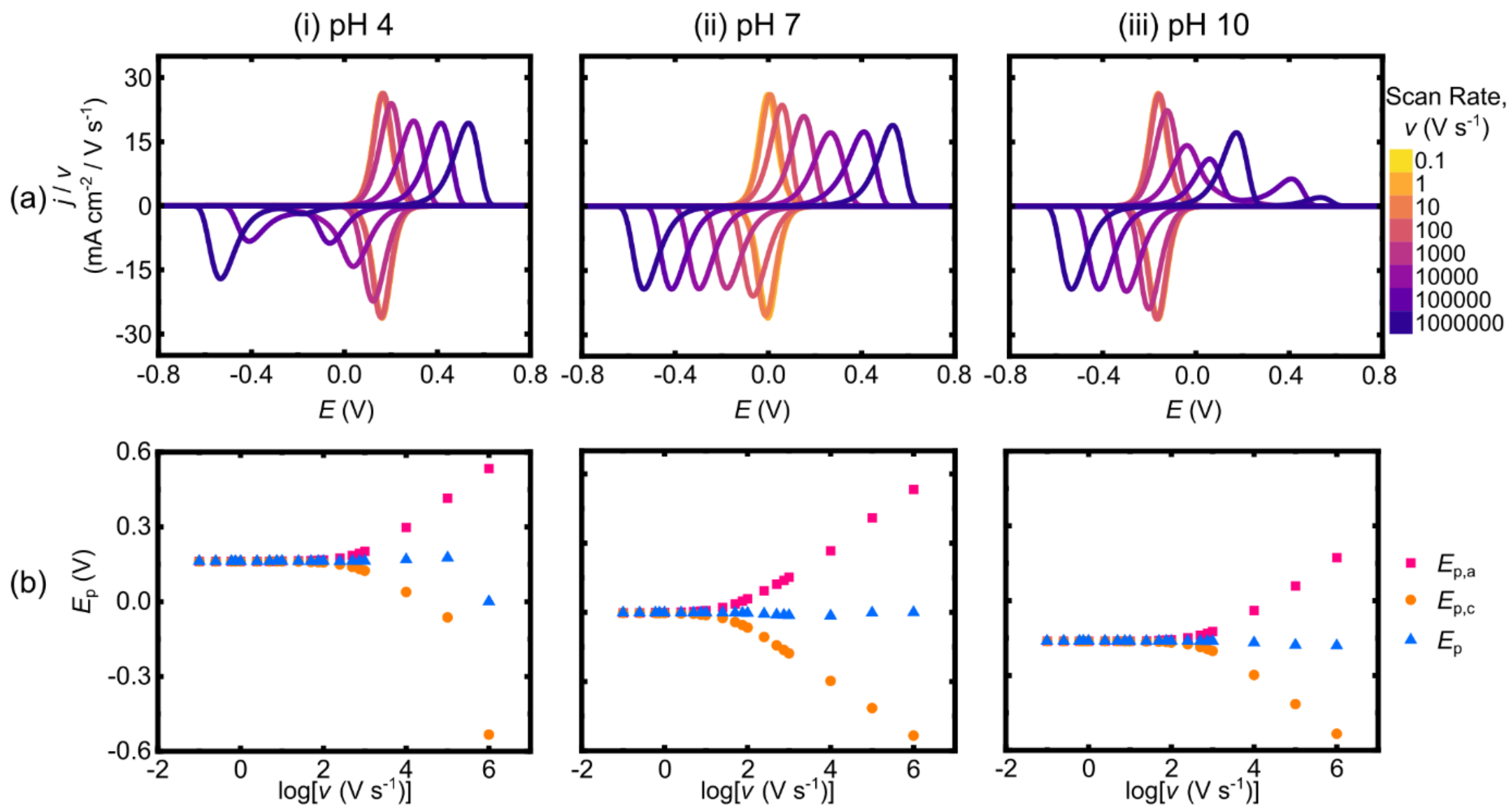

Figure S11. (a) Simulated CV scans with j normalised by v, and (b) Laviron plots at varying v (0.1 – 1 × 10^6 V s^{-1}) for buffered (i) pH 4, (ii) pH 7, and (iii) pH 10. $k_{e,n}{}^0$ = 2 × 10^4 s^{-1}, k_{b,p_n} = 1 × 10^5 s^{-1}, and Γ_{tot} = 2.8 × 10^{-10} mol cm^{-2} (monolayer coverage)

When comparing k_{expt} to $k^*{}_e$, as shown in ***Figure S12***, it is clear that k_{expt} does not follow $k^*{}_e$ when k_{b,p_n} = 1 × 10^5 s^{-1}, unlike when k_{b,p_n} = 1 × 10^9 s^{-1} (see ***Figure 7*** in main text). This determines that deviations of k_{expt} found using the Laviron plot method are directly related to slow PT rates.

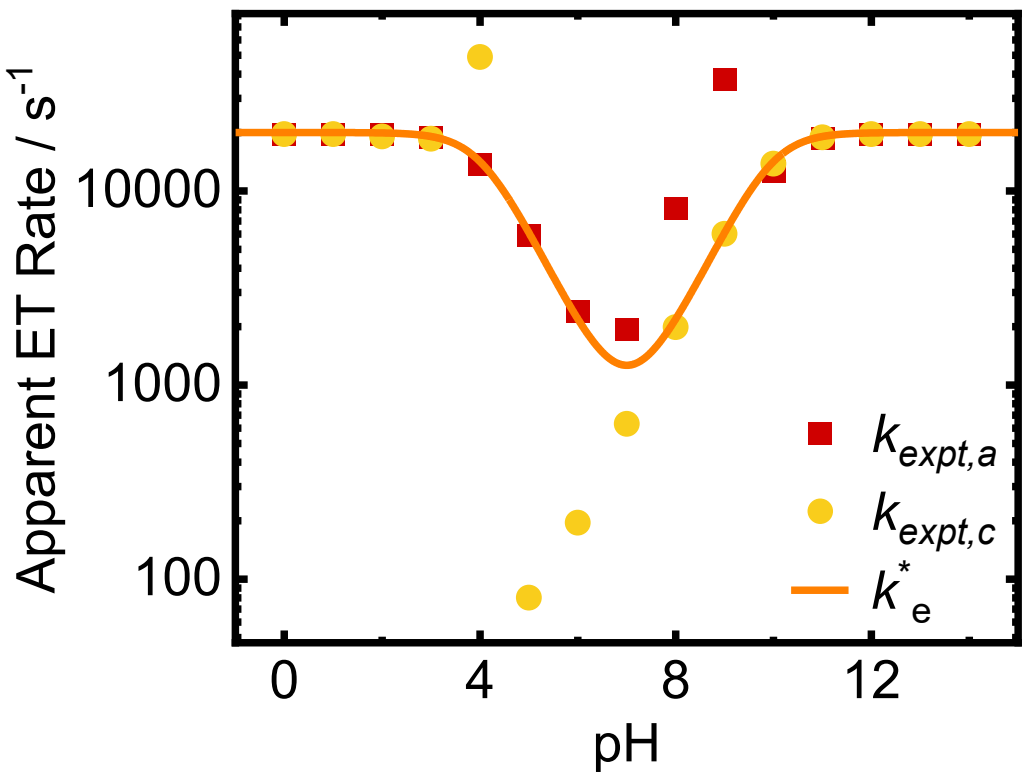

Figure S12. Comparison of experimentally determined k_{expt} (a = anodic, c = cathodic) to the analytically determined k*e for a 1e^-1H^+ system over the pH range 0 – 14. $k_{e,n}{}^0 = 2 \times 10^4$ s^{-1}, k_{b,p_n} = 1×10^5 s^{-1}, and $\Gamma_{tot} = 2.8 \times 10^{-10}$ mol cm^{-2} (monolayer coverage)

SI-12: Surface Concentrations During Different Timescales

Figure S13 shows how the surface concentration of species (M to V, Laviron notation, Figure 1 main text) changes during CV scans over different timescales (scan rates), (a) scan rate of 0.1 V s^{-1} (total scan time 48 s); (b) scan rate of 1×10^6 V s^{-1} (total scan time 4.8 ns). In (a) species P, formed from M (and to a lesser extent O), appears to transition immediately to species V. However, at very short timescales, it can be seen that the transition is not immediate and the coverage of P builds up until almost all surface species are in the P state. As the surface coverage of P then decreases, V increases. This results in two peaks being observed in the reductive part of the CV scan, ***Figure S13c***.

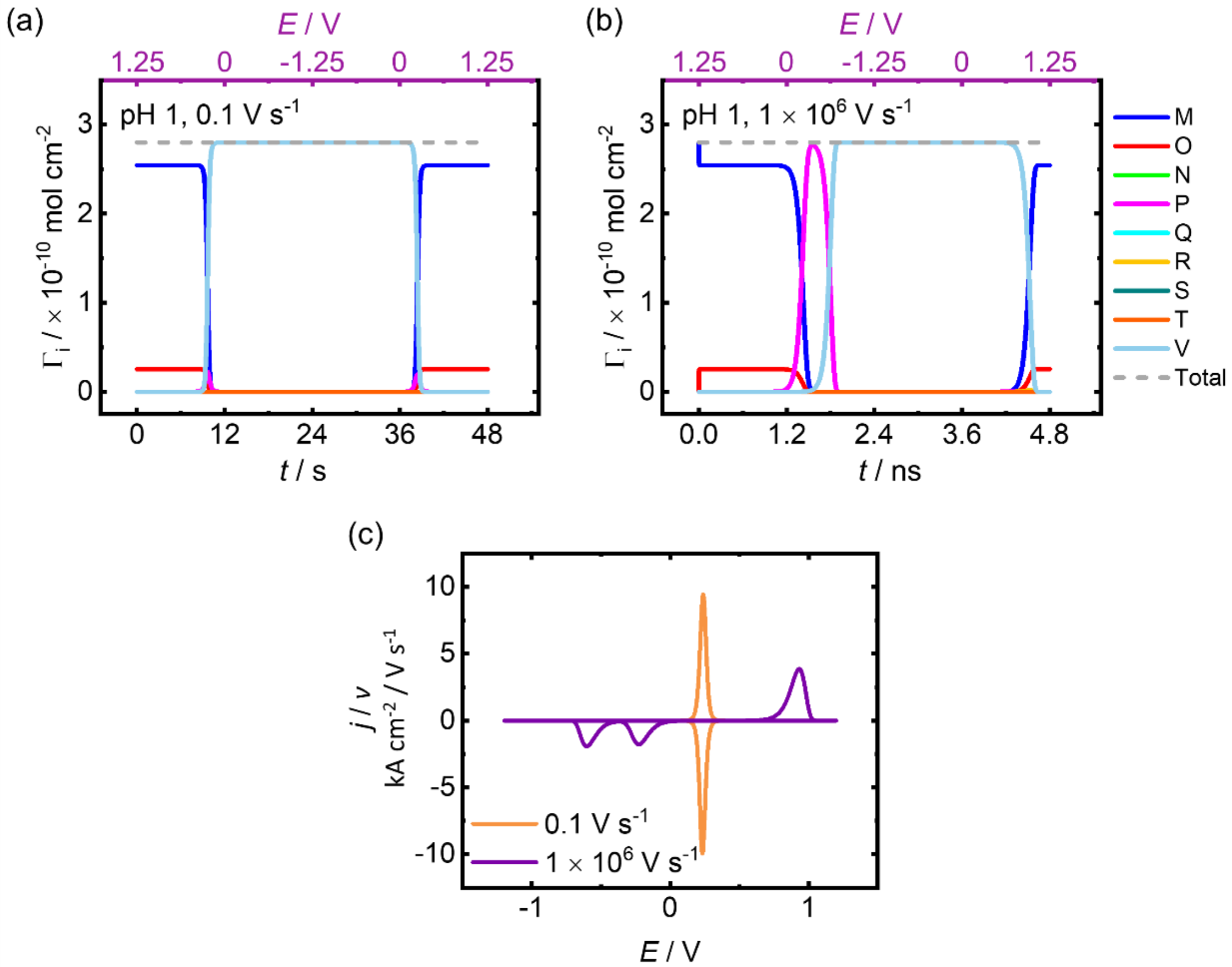

Figure S13. Surface species concentration plots for buffered pH 1 solutions with scan rates of (a) 0.1 V s^{-1} and (b) 1×10^6 V s^{-1}. (c) The CV scans with v normalised current density, j, for scan rates of 0.1 (yellow) and 1×10^6 (purple) V s^{-1}. $\Gamma_{tot} = 2.8 \times 10^{-10}$ mol cm^{-2} (monolayer coverage)

SI-13: Laviron Plot Data Tables

Table S4 shows the m, α, v, and k_{expt} data obtained from the simulated Laviron plots for a 1e^- 1H^+ PCET system where fast protonation/deprotonation rates (k_{b,p_n} = 1 × 10^9 s^{-1}) are used.[6] α values of predominantly 0.5 are obtained and $k_{expt,c}$ values reflect k^*_e values (see ***Figure 7*** in main text).

Table S4. 1e^-1H^+ data obtained from Laviron plots between pH 0 to 14, $k_{e,n}{}^0$ = 2 × 10^4 s^{-1}, k_{b,p_n} = 1 × 10^9 s^{-1}. All values are given to 3.s.f.

	Anodic				Cathodic			
pH	m_a / s	α_a	v_a / V s^{-1}	$k_{expt,a}$ / s^{-1}	m_c / s	α_c	v_c / V s^{-1}	$k_{expt,c}$ / s^{-1}
0	0.118	0.501	1010	19600	-0.118	0.501	1010	19600
1	0.118	0.501	1010	19600	-0.118	0.501	1010	19600
2	0.118	0.501	1010	19600	-0.118	0.503	974	19100
3	0.118	0.501	968	18800	-0.118	0.503	937	18300
4	0.119	0.499	728	14200	-0.118	0.501	718	14000
5	0.119	0.499	314	6120	-0.118	0.501	310	6140
6	0.118	0.501	110	2140	-0.124	0.477	142	2640
7	0.118	0.501	63.8	1240	-0.135	0.499	125	2140
8	0.118	0.501	110	2140	-0.121	0.490	121	2320
9	0.118	0.501	310	6030	-0.119	0.497	319	6170
10	0.118	0.501	718	13900	-0.119	0.499	728	14100
11	0.118	0.503	937	18100	-0.118	0.501	968	18900
12	0.118	0.503	974	18900	-0.118	0.501	1010	19600
13	0.118	0.501	1010	19600	-0.118	0.501	1010	19600
14	0.118	0.501	1010	19600	-0.118	0.501	1010	19600

Table S5 shows the m, α, v, and k_{expt} data obtained from the simulated Laviron plots for a 2e^- 2H^+ PCET system. At extreme pH values (pH -10 and +20), α values of 0.25 are calculated. In these pH regions, there are no PT reactions occurring, only two step ET reactions ($Q \rightarrow Q^{\cdot -} \rightarrow Q^{2-}$, and $QH_2{}^{2+} \rightarrow QH_2{}^{\cdot +} \rightarrow QH_2$ respectively). This determines that the 2nd ET step is rate limiting for α_a, and the 1st ET step is rate limiting for α_c.[23] For α values in the pH range 0 – 14, the two ET steps are now convoluted significantly with PT steps, which is observed as the deviation from ideal α values (0.25 or 0.75) for a two-step 2e^- transfer reaction.[23]

Table S5. $2e^{-}2H^{+}$ data obtained from Laviron plots between pH -10 to +20, $k_{e,n}{}^{0} = 2 \times 10^{4}$ s^{-1}, $k_{b,p_n} = 1 \times 10^{9}$ s^{-1}. All values are given to 3.s.f.

	Anodic				Cathodic			
pH	m_a / s	α_a	v_a / V s^{-1}	$k_{expt,a}$ / s^{-1}	m_c / s	α_c	v_c / V s^{-1}	$k_{expt,c}$ / s^{-1}
-10	0.118	0.250	400	23300	-0.118	0.250	408	7940
0	0.120	0.246	3.76	221	-0.0461	0.641	4.48	223
1	0.116	0.254	1.19	69.0	-0.0797	0.371	29.9	863
2	0.120	0.247	0.502	29.0	-0.106	0.278	26.6	575
3	0.116	0.254	0.232	13.5	-0.116	0.255	10.3	204
4	0.119	0.248	0.479	28.1	-0.122	0.242	2.47	46.7
5	0.120	0.247	1.41	82.5	-0.106	0.278	0.740	16.0
6	0.120	0.247	2.41	141	-0.140	0.212	0.215	3.55
7	0.113	0.261	7.22	415	-0.123	0.240	0.185	3.47
8	0.110	0.270	25.8	1470	-0.120	0.247	0.452	8.69
9	0.0824	0.358	31.4	1570	-0.120	0.247	1.39	27.0
10	0.0340	0.869	0.622	6.35	-0.118	0.247	3.49	68.0
11	0.0697	0.424	15.8	710	-0.120	0.247	7.74	149
12	0.0762	0.388	26.0	1240	-0.120	0.247	13.9	268
13	0.0995	0.297	43.6	2390	-0.120	0.247	24.6	472
14	0.111	0.266	35.4	2020	-0.111	0.266	34.7	719
20	0.118	0.250	408	23800	-0.118	0.250	400	7790

SI-14: PCET Mechanism With Limited H^+

In unbuffered solutions, as the solution pH increases, there are less protons available to take part in the reaction and in unbuffered solutions $[H^+]_{surf}$ depletes over the course of the PCET reaction. ***Figure S14*** provides the surface coverage plots for the quinone species M to V in unbuffered solutions of pH (a) 0, (b) 5, (c) 10, and (d) 14. At pH 0, the plot looks similar to the equivalent one in buffered solution (***Figure S6b***). However, by pH 5 the unbuffered and buffered plots differ significantly, informing that a change of mechanism forced by the lack of protons available at the electrode surface has occurred. Instead of M → P → V as seen in buffered solutions, the mechanism becomes convoluted, and at pH 5 actually releases protons back to the solution, i.e. M → P → V → T/S. As the pH increases, there are almost no protons available, and the mechanism shifts to M → N → S (i.e. $2e^-$ only with no protonation).

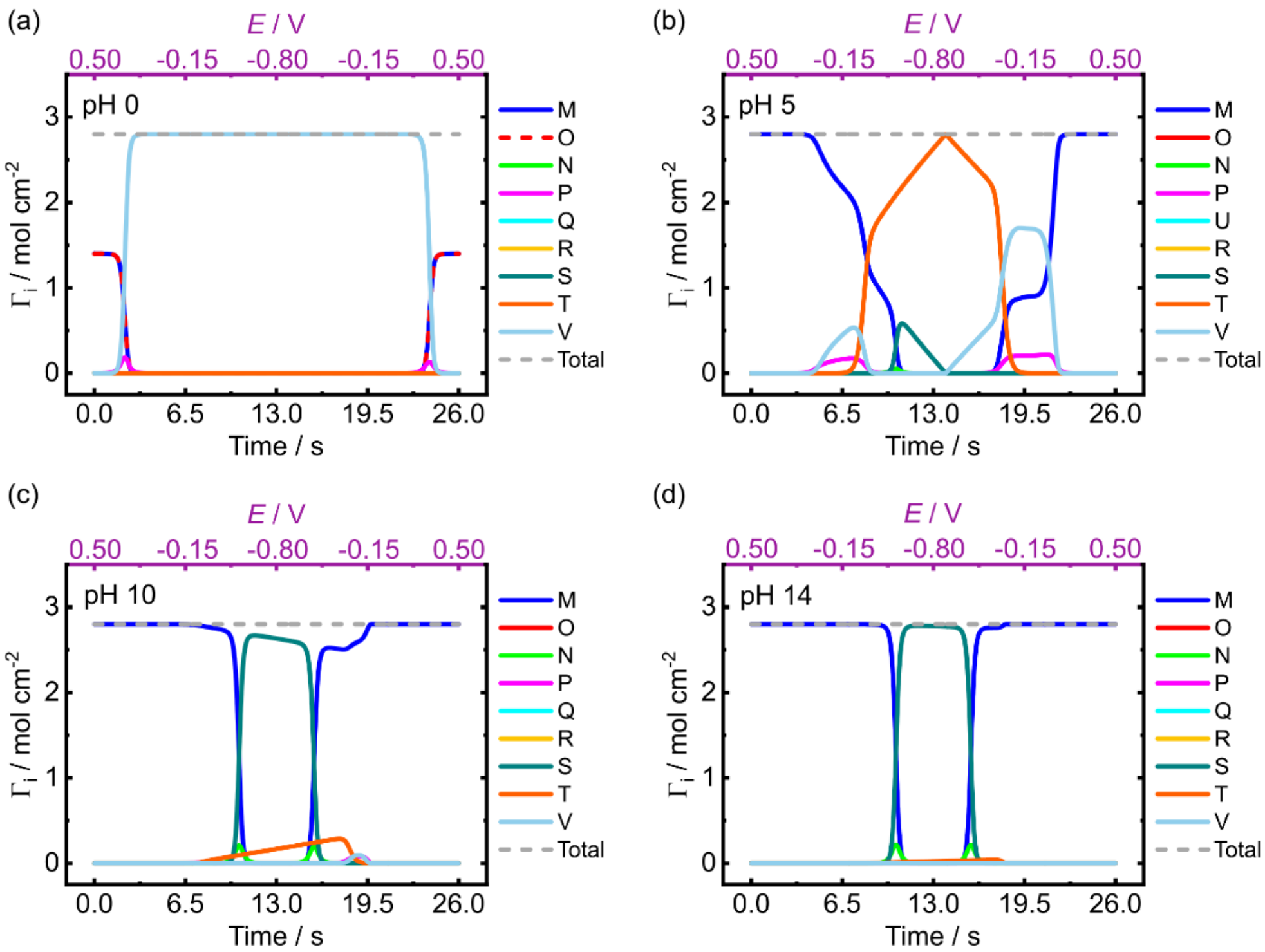

Figure S14. The surface coverage of quinone species ($\Gamma_{tot} = 2.8 \times 10^{-10}$ mol cm^{-2}) over the course of a CV scan in unbuffered solutions of pH (a) 0, (b) 5, (c) 10, and (d) 14.

SI-15: Self-Ionisation of Water Reaction

The self-ionisation of water reaction (***Equation 18*** in main text) becomes most important in unbuffered solutions. Without its presence in the model, different results are observed. This is expected as, at higher pH in unbuffered solutions, where H^+ is much more scarce, without consideration of self-ionisation, H^+ "run out" during PCET reduction. By including the self-ionisation of water reaction, a method to replenish H^+ is provided to the system. See ***Figure S15*** for a comparison of the simulated CV results obtained when the self-ionisation of water reaction is (solid line) and isn't (dashed line) included in the model.

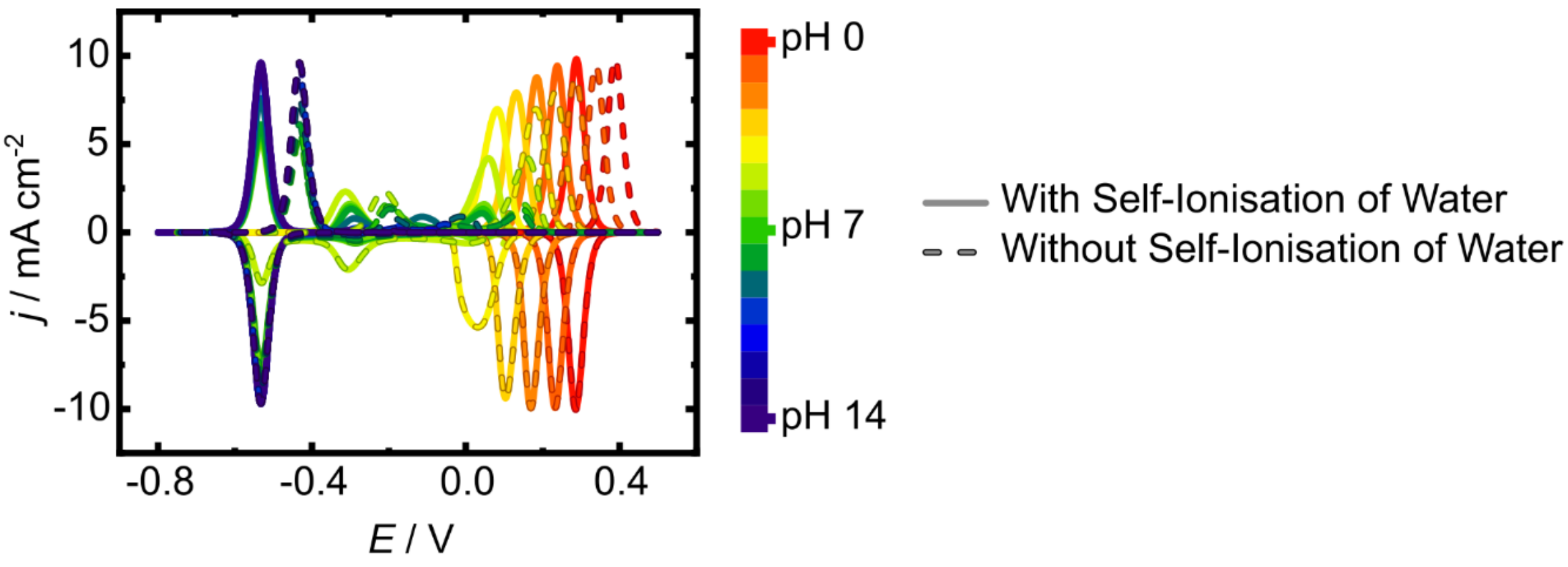

Figure S15. Comparison of the unbuffered CV (0.1 V s^{-1} scan rate) when the self-ionisation of water reaction is (solid line) and isn't (dashed line) included.

SI-16: $[H^+]$ at the Electrode Surface Over Time at Varying Γ_{tot} and pH Values

Figure S16 shows how $[H^+]_{surf}$ varies throughout a CV scan as a function of Γ_{tot} (Γ_{tot} values in the range $1 \times 10^{-14} - 2.8 \times 10^{-10}$ mol cm^{-2} considered) for pH values of (a) 0 (b) 4 (c) 7 (d) 10 and (e) 14. As Γ_{tot} is lowered, the perturbation of $[H^+]_{surf}$ becomes less significant for all pH values and $[H^+]_{surf}$ better reflects $[H^+]_{bulk}$. Note that the largest deviation in $[H^+]$ is seen at pH 7 (up to 15 mmol m^{-3} difference).

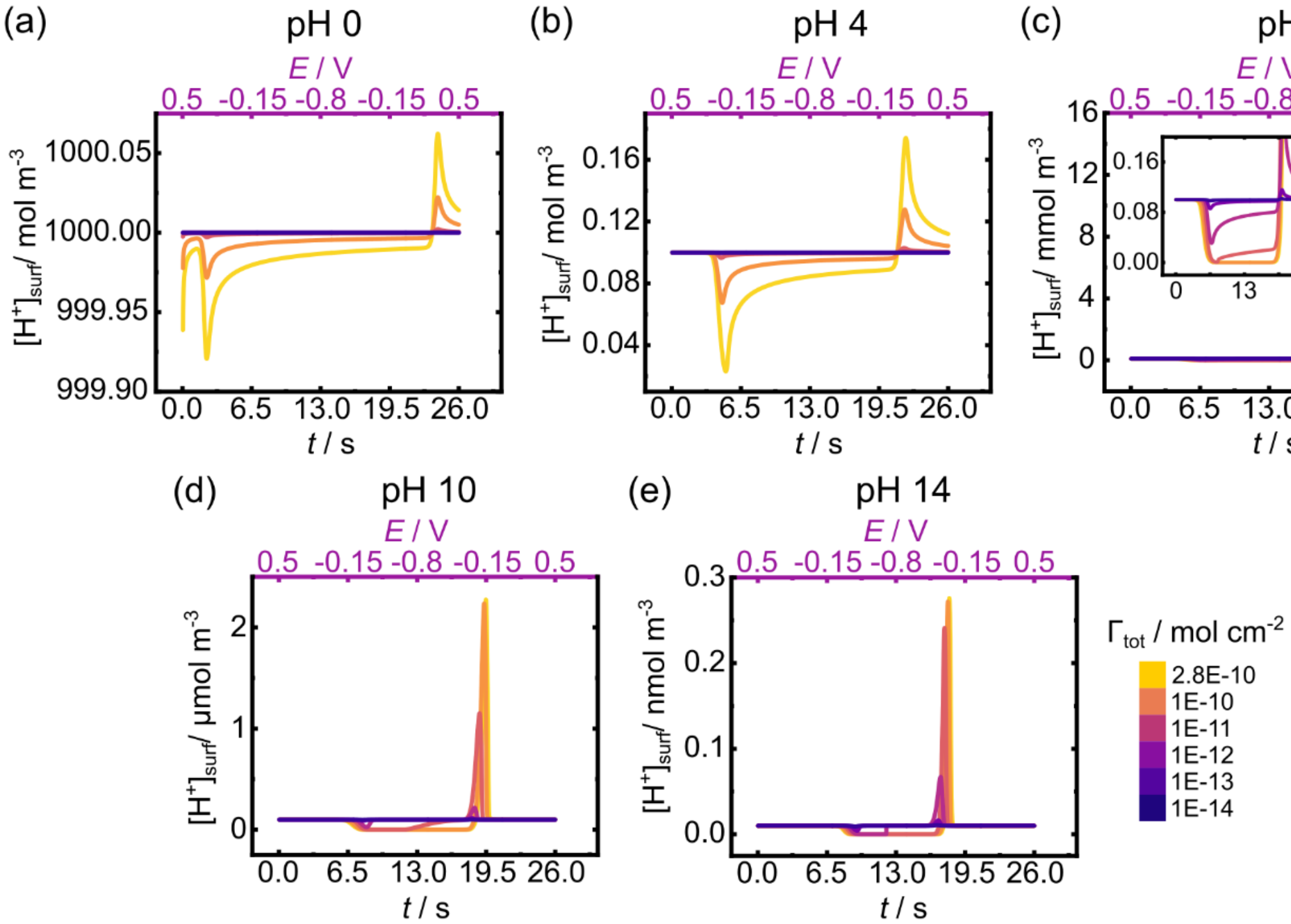

Figure S16. $[H^+]_{surf}$ as a function of electrode potential (and time) for Γ_{tot} values in the $1 \times 10^{-14} - 2.8 \times 10^{-10}$ mol cm^{-2} for (a) pH 0, (b) pH 4, (c) pH 7, (d) pH 10, and (e) pH 14.

SI-17: E_p *vs* pH for a Range of Γ_{tot}

Figure S17 shows that by decreasing Γ_{tot}, E_p *vs* pH follows the expected $2e^-2H^+$ linear response over the pH range 0-10. This is achieved for Γ_{tot} values ≤ 1 × 10^{-13} mol cm^{-2}.

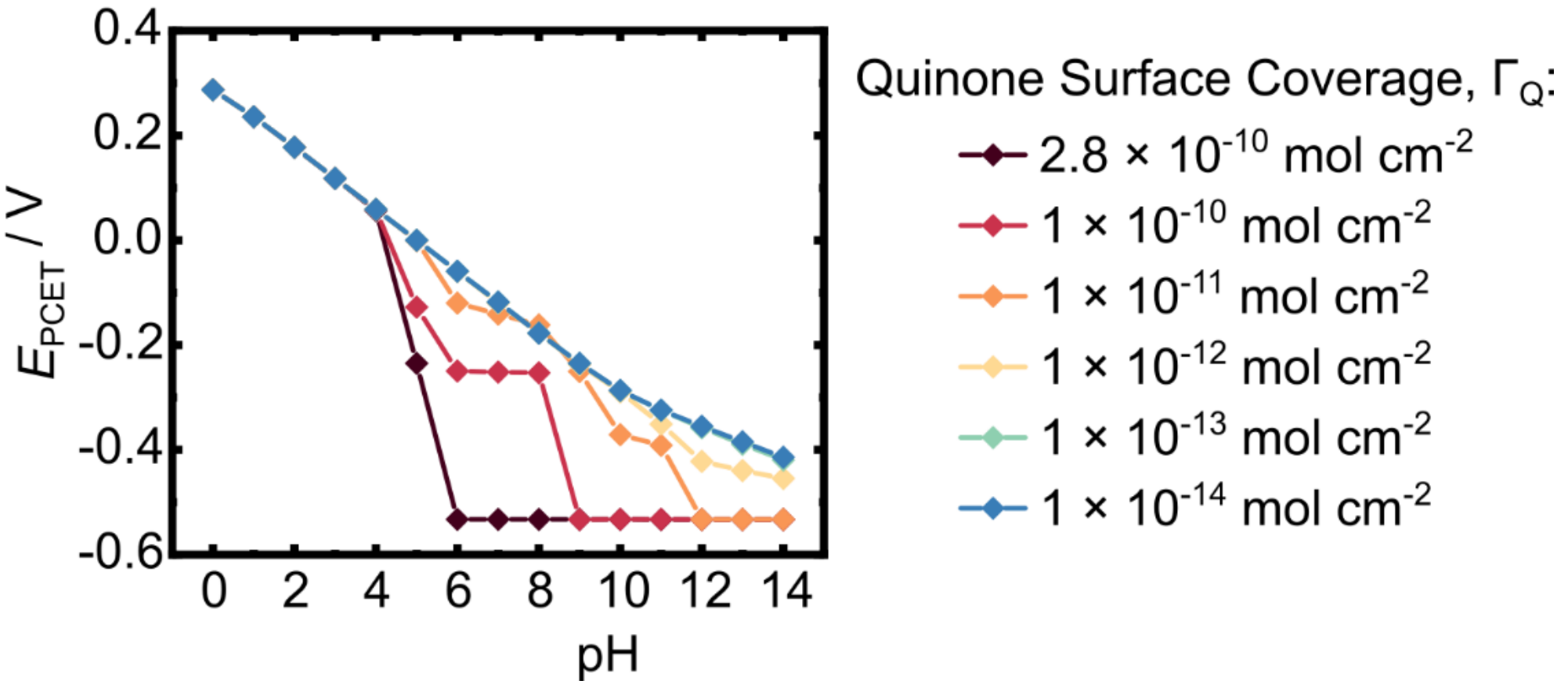

Figure S17. E_p *vs* pH for various Γ_{tot} in the range 2.8 × 10^{-10} to 1 × 10^{-14} mol cm^{-2}. Lines are for visualisation purposes only and not lines of best fit.

SI-18: Effect of Scan Rate on the Unbuffered PCET Reaction

The effect of changing scan rate, v, on the CV in unbuffered solution is demonstrated in ***Figure S18***. Γ_{tot} is fixed at monolayer coverage (2.8×10^{-10} mol cm^{-2}). ***Figure S18a*** shows that increasing v to 100 V s^{-1} increases ΔE_p and quinone peaks are deconvoluted. Decreasing v to 1×10^{-6} V s^{-1} results in symmetrical single peaks, ***Figure S18b***, where a slope of -62 mV / pH unit and an R^2 value of 0.995 is observed between pH 0 – 10.

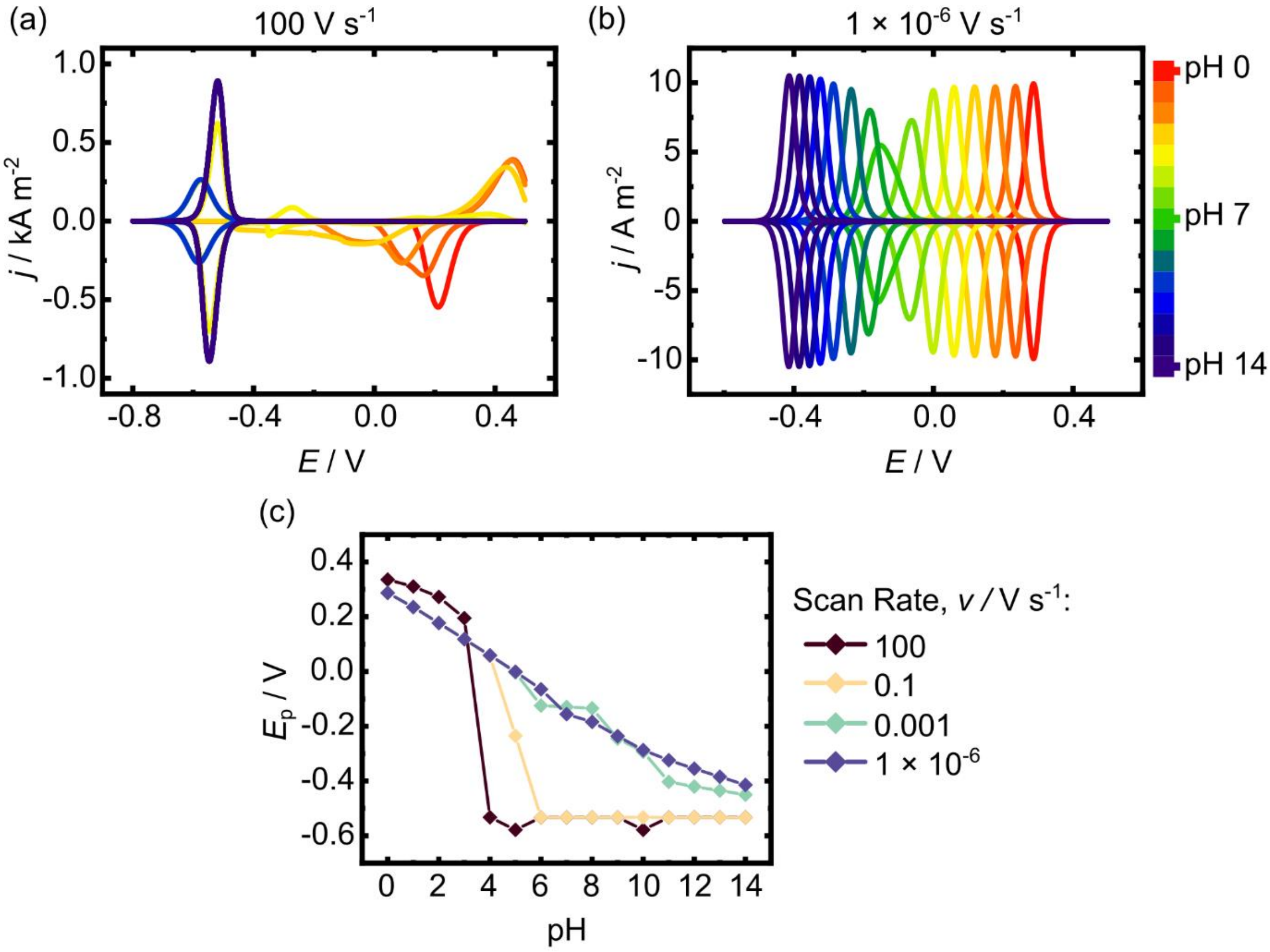

Figure S18. Simulated CVs in unbuffered solution from pH 0 – 14 when Γ_{tot} is 2.8×10^{-10} mol cm^{-2} when v is set to (a) 100 V s^{-1} and (b) 1×10^{-6} V s^{-1}. (c) E_p of the simulated CVs at different v values. Lines in (c) are for visualisation purposes only and not best fit curves.

References

1 P. R. Unwin and J. V. Macpherson, Determination of the diffusion coefficient of hydrogen in aqueous solution using single and double potential step chronoamperometry at a disk ultramicroelectrode, *Anal. Chem.*, 1997, **69**, 2063–2069.

2 R. N. Goldberg, N. Kishore and R. M. Lennen, *CRC Handbook of Chemistry and Physics*, CRC Press, 95th edn., 2014.

3 Y. Sato, M. Fujita, F. Mizutani and K. Uosaki, Electrochemical properties of the 2-mercaptohydroquinone monolayer on a gold electrode. Effect of solution pH, adsorption time and concentration of the modifying solution, *Journal of Electroanalytical Chemistry*, 1996, **409**, 145–154.

4 N. Elgrishi, K. J. Rountree, B. D. McCarthy, E. S. Rountree, T. T. Eisenhart and J. L. Dempsey, A Practical Beginner's Guide to Cyclic Voltammetry, *J. Chem. Educ.*, 2018, **95**, 197–206.

5 E. Laviron, Electrochemical reactions with protonations at equilibrium: Part VIII. The $2e^-$, $2H^+$ Reaction (Nine-Member Square Scheme) For a Surface or For a Heterogeneous Reaction in the Absence of Disproportionation and Dimerization Reactions, *J. Electroanal. Chem. Interfacial Electrochem.*, 1983, **146**, 15–36.

6 M. Eigen, Proton Transfer, Acid-Base Catalysis, and Enzymatic Hydrolysis. Part I: ELEMENTARY PROCESSES, *Angewandte Chemie International Edition in English*, 1964, **3**, 1–19.

7 S. J. Cobb, Z. J. Ayres, M. E. Newton and J. V. Macpherson, Deconvoluting Surface-Bound Quinone Proton Coupled Electron Transfer in Unbuffered Solutions: Toward a Universal Voltammetric pH Electrode, *J. Am. Chem. Soc.*, 2019, **141**, 1035–1044.

8 M. Eigen and L. De Maeyer, Untersuchungen über die Kinetik der Neutralisation. I, *Zeitschrift für Elektrochemie, Berichte der Bunsengesellschaft für physikalische Chemie*, 1955, **59**, 986–993.

9 R. P. Bell, *The Proton in Chemistry*, Springer US, Boston, MA, 1st edn., 1973.

10 A. Hashemi, R. Khakpour, A. Mahdian, M. Busch, P. Peljo and K. Laasonen, Density functional theory and machine learning for electrochemical square-scheme prediction: an application to quinone-type molecules relevant to redox flow batteries, *Digital Discovery*, 2023, **2**, 1565–1576.

11 E. Katz and I. Willner, Kinetic separation of amperometric responses of composite redox-active monolayers assembled onto Au electrodes: Implications to the monolayers' structure and composition, *Langmuir*, 1997, **13**, 3364–3373.

12 G. March, S. Reisberg, B. Piro, M. C. Pham, M. Delamar, V. Noel, K. Odenthal, D. B. Hibbert and J. J. Gooding, Electrochemical kinetic analysis of a 1,4-hydroxynaphthoquinone self-assembled monolayer, *Journal of Electroanalytical Chemistry*, 2008, **622**, 37–43.

13 T. Mebrahtu, G. M. Berry, B. G. Bravo, S. L. Michelhaugh and M. P. Soriaga, Substrate-Mediated Adsorbate-adsorbate interactions: Effect of submonolayer coverage

and coadsorbed iodine on the reversible redox of 2,5-dihydroxythiophenol chemisorbed at Au and Pt, *Langmuir*, 1988, **4**, 1147–1151.

14 S. P. O. Neill, A. Martínez-Aviñó, C. Keane, S. Hassan, C. Houston, S. Denuga, E. B. Farrell, G. Gil-Ramírez and R. P. Johnson, Comparative Proton Coupled Electron Transfer at Glassy Carbon and Boron-Doped Diamond Electrodes, *ChemElectroChem*, DOI:10.1002/celc.202300470.

15 L. Bouffier, K. E. Lister, S. J. Higgins, R. J. Nichols and T. Doneux, Electrochemical investigations of dissolved and surface immobilised 2-amino-1,4-naphthoquinones in aqueous solutions, *Journal of Electroanalytical Chemistry*, 2012, **664**, 80–87.

16 R. J. Forster and J. P. O'Kelly, Protonation reactions of anthraquinone-2,7-disulphonic acid in solution and within monolayers, *Journal of Electroanalytical Chemistry*, 2001, **498**, 127–135.

17 S. A. Trammell and N. Lebedev, Proton-coupled electron transfer in self-assembled monolayers containing quinone compounds with different bridging groups of varying electronic conjugation, *Journal of Electroanalytical Chemistry*, 2009, **632**, 127–132.

18 S. A. Trammell, D. A. Lowy, D. S. Seferos, M. Moore, G. C. Bazan and N. Lebedev, Heterogeneous electron transfer of quinone-hydroquinone in alkaline solutions at gold electrode surfaces: Comparison of saturated and unsaturated bridges, *Journal of Electroanalytical Chemistry*, 2007, **606**, 33–38.

19 A. D. Abhayawardhana and T. C. Sutherland, Heterogeneous proton-coupled electron transfer of an aminoanthraquinone self-assembled monolayer, *Journal of Physical Chemistry C*, 2009, **113**, 4915–4924.

20 N. Darwish, P. K. Eggers, S. Ciampi, Y. Tong, S. Ye, M. N. Paddon-Row and J. J. Gooding, Probing the Effect of the Solution Environment around Redox-Active Moieties Using Rigid Anthraquinone Terminated Molecular Rulers, *J. Am. Chem. Soc.*, 2012, **134**, 18401–18409.

21 W. Zhang, S. M. Rosendahl and I. J. Burgess, Coupled Electron/Proton Transfer Studies of Benzoquinone-Modified Monolayers, *The Journal of Physical Chemistry C*, 2010, **114**, 2738–2745.

22 E. Laviron, Electrochemical reactions with protonations at equilibrium: Part VII. The $2e^-$, $1H^+$ Reaction (Six-Member Fence Scheme) For a Surface or For a Heterogeneous Reaction in the Absence of Disproportionation and Dimerization, *J. Electroanal. Chem. Interfacial Electrochem.*, 1983, **146**, 1–13.

23 M. A. V. Devanathan, Kinetics of multiple-electron-transfer reactions-I. The transfer coefficient, *Electrochim. Acta*, 1972, **17**, 1683–1697.
