# Supplementary material for: Finite element modelling of surface-bound quinone proton coupled electron transfer voltammetry
Source: Phys Chem Chem Phys. 2026 Aug 3;28(34):20742–54. doi: 10.1039/d6cp01907b (PMC13473934; doi:10.1039/d6cp01907b)
Supplement: Supplementary file 2 [file CP-028-D6CP01907B-s002.pdf]

Supplementary Information (SI) for Physical Chemistry Chemical Physics.
This journal is © the Owner Societies 2026

COMSOL MULTIPHYSICS®

241018 2e2H Model Final to export

Report date	Oct 9, 2025, 4:57:13 PM

Contents

1 Global Definitions 3

1.1 Parameters 3

1.2 Shared Properties 5

2 Component 1 6

2.1 Definitions 6

2.2 Geometry 1 12

2.3 Transport of Diluted Species 13

2.4 Surface Reactions 27

2.5 Mesh 1 35

3 Study 1 39

3.1 Parametric Sweep 1 39

3.2 Stationary 39

3.3 Time Dependent 40

3.4 Solver Configurations 40

4 Results 50

4.1 Datasets 50

4.2 Derived Values 53

4.3 Tables 53

4.4 Plot Groups 53

1 Global Definitions

Date	Sep 1, 2025, 4:15:11 PM

GLOBAL SETTINGS

Name	241018 2e2H Model Final to export.mph
Path	F:\Nafiz\Quinone Modelling\Final\241018_2e2H Model_Final_to export.mph
Version	COMSOL Multiphysics 6.2 (Build: 415)

USED PRODUCTS

Chemical Reaction Engineering Module
COMSOL Multiphysics

COMPUTER INFORMATION

CPU	Intel64 Family 6 Model 158 Stepping 9, 4 cores, 63.88 GB RAM
Operating system	Windows 10

1.1 PARAMETERS

1.1.1 Bulk pH

BULK PH

Name	Expression	Value	Description
pH	7	7	Bulk pH
pOH	14 - pH	7	Bulk pOH

1.1.2 kb Values

KB VALUES

Name	Expression	Value	Description
k1_b	kb	1E9 1/s	Deprotonation rate (Q -> O)
k2_b	kb	1E9 1/s	Deprotonation rate (R -> P)
k3_b	kb	1E9 1/s	Deprotonation rate (O -> M)
k4_b	kb	1E9 1/s	Deprotonation rate (P -> N)
k5_b	kb	1E9 1/s	Deprotonation rate (V -> T)
k6_b	kb	1E9 1/s	Deprotonation rate (T -> S)
kb	1E9[1/s]	1E9 1/s	Standard deprotonation rate

1.1.3 Electron Transfer Rates

ELECTRON TRANSFER RATES

Name	Expression	Value	Description
k_e	2E4[1/s]	20000 1/s	Standard e- transfer rate
k_e3	k_e	20000 1/s	e- transfer rate (M <-> N)
k_e4	k_e	20000 1/s	e- transfer rate (R <-> V)
k_e1	k_e	20000 1/s	e- transfer rate (Q <-> R)
k_e2	k_e	20000 1/s	e- transfer rate (O <-> P)
k_e5	k_e	20000 1/s	e- transfer rate (P <-> T)
k_e6	k_e	20000 1/s	e- transfer rate (N <-> S)

1.1.4 Alpha Values

ALPHA VALUES

Name	Expression	Value	Description
alpha_3	alpha	0.5	Alpha coefficient (M to N)
alpha_4	alpha	0.5	Alpha coefficient (R to V)
alpha_1	alpha	0.5	Alpha coefficient (Q to R)
alpha_2	alpha	0.5	Alpha coefficient (O to P)
alpha_5	alpha	0.5	Alpha coefficient (P to T)
alpha_6	alpha	0.5	Alpha coefficient (N to S)
alpha	0.5	0.5	Alpha coefficient

1.1.5 System Parameters

SYSTEM PARAMETERS

Name	Expression	Value	Description
D_Hsol	5E-5[cm^2/s]	5E−9 m^2/s	Diffusion coefficient of H+ in solution
D_OHsol	5.27E-9 [m^2/s]	5.27E−9 m^2/s	Diffusion coefficient of OH in water
f	F_const/(R_const*Temp)	38.922 1/V	Constant
H_bulk	10^ - pH[M]	1E−4 mol/m^3	Bulk proton concentration
kH	10E9[M/s]	1E13 $mol/(m^3 \cdot s)$	Proton rate constant
mesh_1	1E-8[m]	1E−8 m	Mesh size at electrode surface
n	1	1	No. of electrons transferred
OH_bulk	10^ - pOH[M]	1E−4 mol/m^3	Bulk OH- concentration
SiteDensity	2.8E-10[mol/cm^2]	2.8E−6 mol/m^2	Total site density
Temp	298.15 [K]	298.15 K	Temperature
Tol	1E-3	0.001	Relative tolerance value

1.1.6 CV Parameters

CV PARAMETERS

Name	Expression	Value	Description
ScanRate	0.1 [V/s]	0.1 V/s	Scan rate
Ein	+0.5 [V]	0.5 V	Initial potential
Es	-0.8 [V]	−0.8 V	Switch potential
ScanRange	abs(Ein - Es)	1.3 V	Scan potential range
SegmentPeriod	ScanRange/ScanRate	13 s	Length of each segment
incr	0.001[V]	0.001 V	Scan increment
t_step	incr/ScanRate	0.01 s	Time step between each data point

1.1.7 Water Reaction Rates

WATER REACTION RATES

Name	Expression	Value	Description
Kw_water	1E-14[mol^2*dm^ - 6]	1E−8 mol^2/m^6	Equilibrium rate constant for water
k_d	2.6E-5[s^ - 1]	2.6E−5 1/s	Forward reaction for water self-ionisation
k_r	1.4E11[dm^3/(mol*s)]	1.4E8 $m^3/(s{\cdot}mol)$	Backward reaction for water self-ionisation
C_w	55.5[mol/dm^3]	55500 mol/m^3	Concentration of water

1.2 SHARED PROPERTIES

1.2.1 Default Model Inputs

Tag	cminpt

2 Component 1

SETTINGS

Description	Value
Unit system	Same as global system (SI)

2.1 DEFINITIONS

2.1.1 Variables

pKa Values - Laviron

SELECTION

Geometric entity level	Boundary
Selection	Geometry geom1: Dimension 0: Boundary 1

Name	Expression	Unit	Description
pKa3	0		pKa for M ->O
pKa4	14		pKa for N -> P
pKa1	-8		pKa for O -> Q
pKa2	-4		pKa for P -> R
pKa5	10		pKa for T -> V
pKa6	18		pKa for S -> T

Ka Values

SELECTION

Geometric entity level	Boundary
Selection	Geometry geom1: Dimension 0: Boundary 1

Name	Expression	Unit	Description
Ka3	10^ - pKa3[M]	mol/m^3	Ka for M ->O
Ka4	10^ - pKa4[M]	mol/m^3	Ka for N -> P
Ka1	10^ - pKa1[M]	mol/m^3	Ka for O -> Q
Ka2	10^ - pKa2[M]	mol/m^3	Ka for P -> R
Ka5	10^ - pKa5[M]	mol/m^3	Ka for T -> V
Ka6	10^ - pKa6[M]	mol/m^3	Ka for S -> T

Potential Values

SELECTION

Geometric entity level	Boundary

Selection	Geometry geom1: Dimension 0: Boundary 1

Name	Expression	Unit	Description
E5	E2 - 0.5	V	Overpotential of P ->T
E2	0.25[V]	V	Overpotential of O ->P
E1	E2 + (2.303/f)*(pKa2 - pKa1)	V	Overpotential of Q ->R
E3	E2 - (2.303/f)*(pKa4 - pKa3)	V	Overpotential of M ->N
E4	E5 - (2.303/f)*(pKa2 - pKa5)	V	Overpotential of R ->V
E6	E5 + (2.303/f)*(pKa4 - pKa6)	V	Overpotential of N ->S

Proton Reaction Rates

SELECTION

Geometric entity level	Boundary
Selection	Geometry geom1: Dimension 0: Boundary 1

Name	Expression	Unit	Description
H_elec	max(eps^2, H)	mol/m^3	Proton concentration at electrode surface
k3_f	k3_b*K3	1/s	Protonation rate constant (M -> O)
k4_f	k4_b*K4	1/s	Protonation rate constant (N -> P)
k1_f	k1_b*K1	1/s	Protonation rate constant (O -> Q)
k2_f	k2_b*K2	1/s	Protonation rate constant (P -> R)
k5_f	k5_b*K5	1/s	Protonation rate constant (T -> V)
k6_f	k6_b*K6	1/s	Protonation rate constant (S -> T)
K1	(H_elec/Ka1)		Equilibrium Constant (O -> Q)
K2	(H_elec/Ka2)		Equilibrium Constant (P -> R)
K3	(H_elec/Ka3)		Equilibrium Constant (M -> O)
K4	(H_elec/Ka4)		Equilibrium Constant (N -> P)
K5	(H_elec/Ka5)		Equilibrium Constant (T -> V)
K6	(H_elec/Ka6)		Equilibrium Constant (S -> T)

Electron Reaction Rates

SELECTION

Geometric entity level	Boundary
Selection	Geometry geom1: Dimension 0: Boundary 1

Name	Expression	Unit	Description
k_e3_b	k_e3*exp(-alpha_3*f*(Potential(t) - E3))	1/s	e- rate constant for M to N

Name	Expression	Unit	Description
k_e3_f	k_e3*exp((1 - alpha_3)*f*(Potential(t) - E3))	1/s	e- rate constant for N to M
k_e2_b	k_e2*exp(-alpha_2*f*(Potential(t) - E2))	1/s	e- rate constant for O to P
k_e2_f	k_e2*exp((1 - alpha_2)*f*(Potential(t) - E2))	1/s	e- rate constant for P to O
k_e1_b	k_e1*exp(-alpha_1*f*(Potential(t) - E1))	1/s	e- rate constant for Q to R
k_e1_f	k_e1*exp((1 - alpha_1)*f*(Potential(t) - E1))	1/s	e- rate constant for R to Q
k_e4_b	k_e4*exp(-alpha_4*f*(Potential(t) - E4))	1/s	e- rate constant for R to V
k_e4_f	k_e4*exp((1 - alpha_4)*f*(Potential(t) - E4))	1/s	e- rate constant for V to R
k_e5_b	k_e5*exp(-alpha_5*f*(Potential(t) - E5))	1/s	e- rate constant for P to T
k_e5_f	k_e5*exp((1 - alpha_5)*f*(Potential(t) - E5))	1/s	e- rate constant for T to P
k_e6_b	k_e6*exp(-alpha_6*f*(Potential(t) - E6))	1/s	e- rate constant for N to S
k_e6_f	k_e6*exp((1 - alpha_6)*f*(Potential(t) - E6))	1/s	e- rate constant for S to N

Proton Reactions

SELECTION

Geometric entity level	Boundary
Selection	Geometry geom1: Dimension 0: Boundary 1

Name	Expression	Unit	Description
j_M_Proton	k3_b*O - k3_f*M	mol/(m²·s)	Rate of change of M to O (proton)
j_O_Proton	-(j_M_Proton + j_Q_Proton)	mol/(m²·s)	Rate of change of O (proton)
j_N_Proton	k4_b*P - k4_f*N	mol/(m²·s)	Rate of change of N to P (proton)
j_P_Proton	-(j_N_Proton + j_R_Proton)	mol/(m²·s)	Rate of change of P (proton)
j_Q_Proton	-(k1_b*Q - k1_f*O)	mol/(m²·s)	Rate of change of Q to O (proton)
j_R_Proton	-(k2_b*R - k2_f*P)	mol/(m²·s)	Rate of change of R to P (proton)
j_S_Proton	k6_b*T - k6_f*S	mol/(m²·s)	Rate of change of S to T (proton)
j_V_Proton	-(k5_b*V - k5_f*T)	mol/(m²·s)	Rate of change of V to T (proton)
j_T_Proton	-(j_S_Proton + j_V_Proton)	mol/(m²·s)	Rate of change of T (proton)

Electron Reactions

SELECTION

Geometric entity level	Boundary
Selection	Geometry geom1: Dimension 0: Boundary 1

Name	Expression	Unit	Description
j_O_Electron	k_e2_f*P - k_e2_b*O	mol/(m²·s)	Rate of change of O to P (electron)
j_P_Electron	-(j_O_Electron + j_T_Electron)	mol/(m²·s)	Rate of change of P (electron)
j_M_Electron	k_e3_f*N - k_e3_b*M	mol/(m²·s)	Rate of change of M to N (electron)
j_N_Electron	-(j_M_Electron + j_S_Electron)	mol/(m²·s)	Rate of change of N (electron)
j_Q_Electron	k_e1_f*R - k_e1_b*Q	mol/(m²·s)	Rate of change of Q to R (electron)
j_S_Electron	-(k_e6_f*S - k_e6_b*N)	mol/(m²·s)	Rate of change of S to N (electron)
j_T_Electron	-(k_e5_f*T - k_e5_b*P)	mol/(m²·s)	Rate of change of T to P (electron)
j_V_Electron	-(k_e4_f*V - k_e4_b*R)	mol/(m²·s)	Rate of change of V to R (electron)
j_R_Electron	-(j_Q_Electron + j_V_Electron)	mol/(m²·s)	Rate of change of R (electron)

Combined Reactions

SELECTION

Geometric entity level	Boundary
Selection	Geometry geom1: Dimension 0: Boundary 1

Name	Expression	Unit	Description
j_M	j_M_Proton + j_M_Electron	mol/(m²·s)	Rate of change of M
j_O	j_O_Proton + j_O_Electron	mol/(m²·s)	Rate of change of O
j_N	j_N_Proton + j_N_Electron	mol/(m²·s)	Rate of change of N
j_P	j_P_Proton + j_P_Electron	mol/(m²·s)	Rate of change of P
j_Q	j_Q_Proton + j_Q_Electron	mol/(m²·s)	Rate of change of Q
j_R	j_R_Proton + j_R_Electron	mol/(m²·s)	Rate of change of R
j_S	j_S_Proton + j_S_Electron	mol/(m²·s)	Rate of change of S
j_T	j_T_Proton + j_T_Electron	mol/(m²·s)	Rate of change of T
j_V	j_V_Proton + j_V_Electron	mol/(m²·s)	Rate of change of V

Self-Ionisation of Water Variables

SELECTION

Geometric entity level	Domain
Selection	Geometry geom1: Dimension 1: Domain 1

Name	Expression	Unit	Description
R_cOH	k_d*C_w - (k_r*H*OH)	mol/(m³·s)	Rate of formation of H and OH due to self-ionisation of water
IonicStrength	0.5*(H - OH)	mol/m³	Ionic strength

2.1.2 Functions

CV Voltage

Function name	Potential
Function type	Piecewise

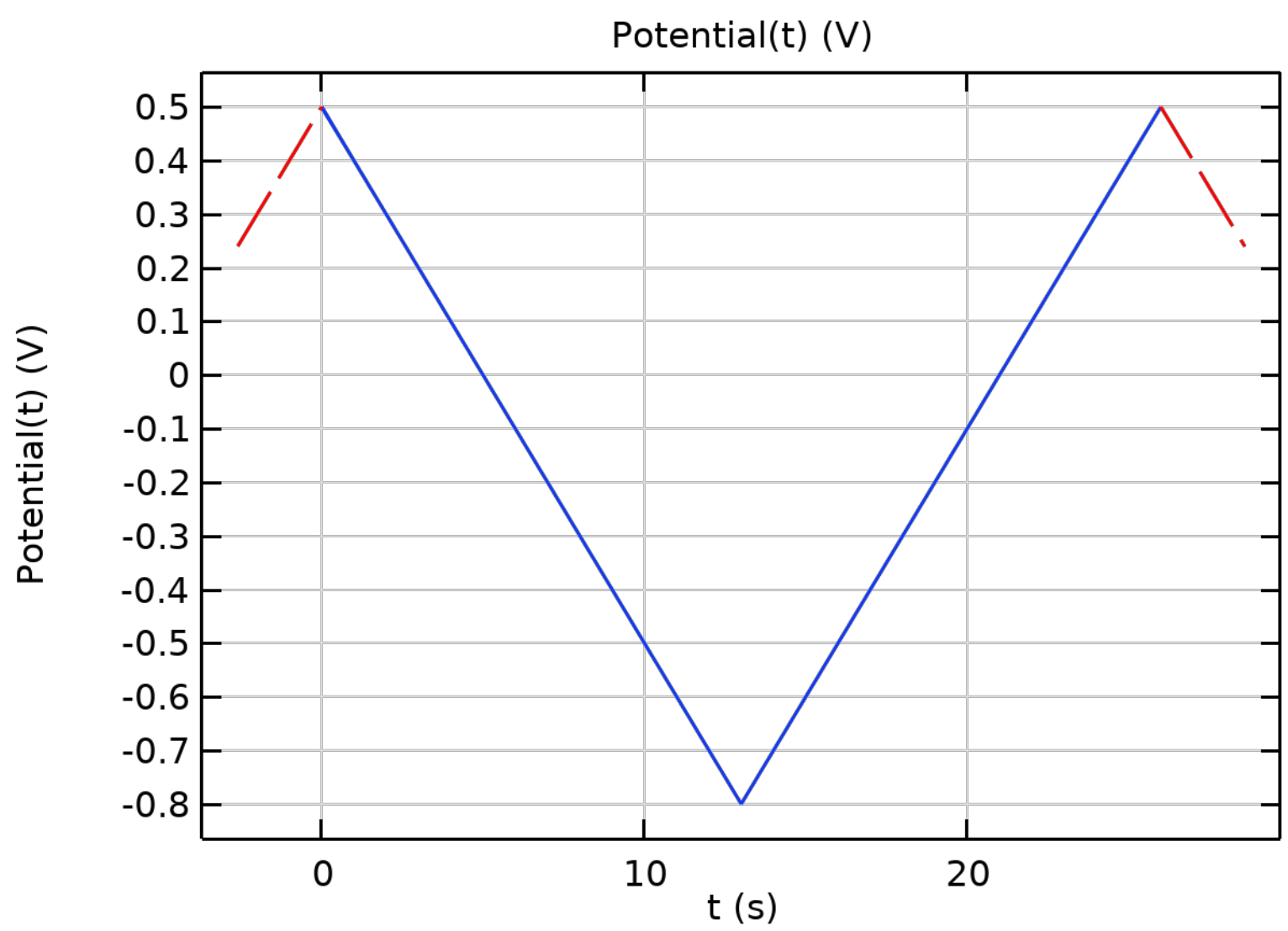

CV Voltage

DEFINITION

Description	Value
Argument	t
Extrapolation	Periodic
Smoothing	No smoothing

DEFINITION

Start	End	Function
0	SegmentPeriod	Ein-ScanRate*t
SegmentPeriod	2*SegmentPeriod	Es+ScanRate*(t-SegmentPeriod)

UNITS

Description	Value
Arguments	s
Function	V

Analytic 1

Function name	an1
Function type	Analytic

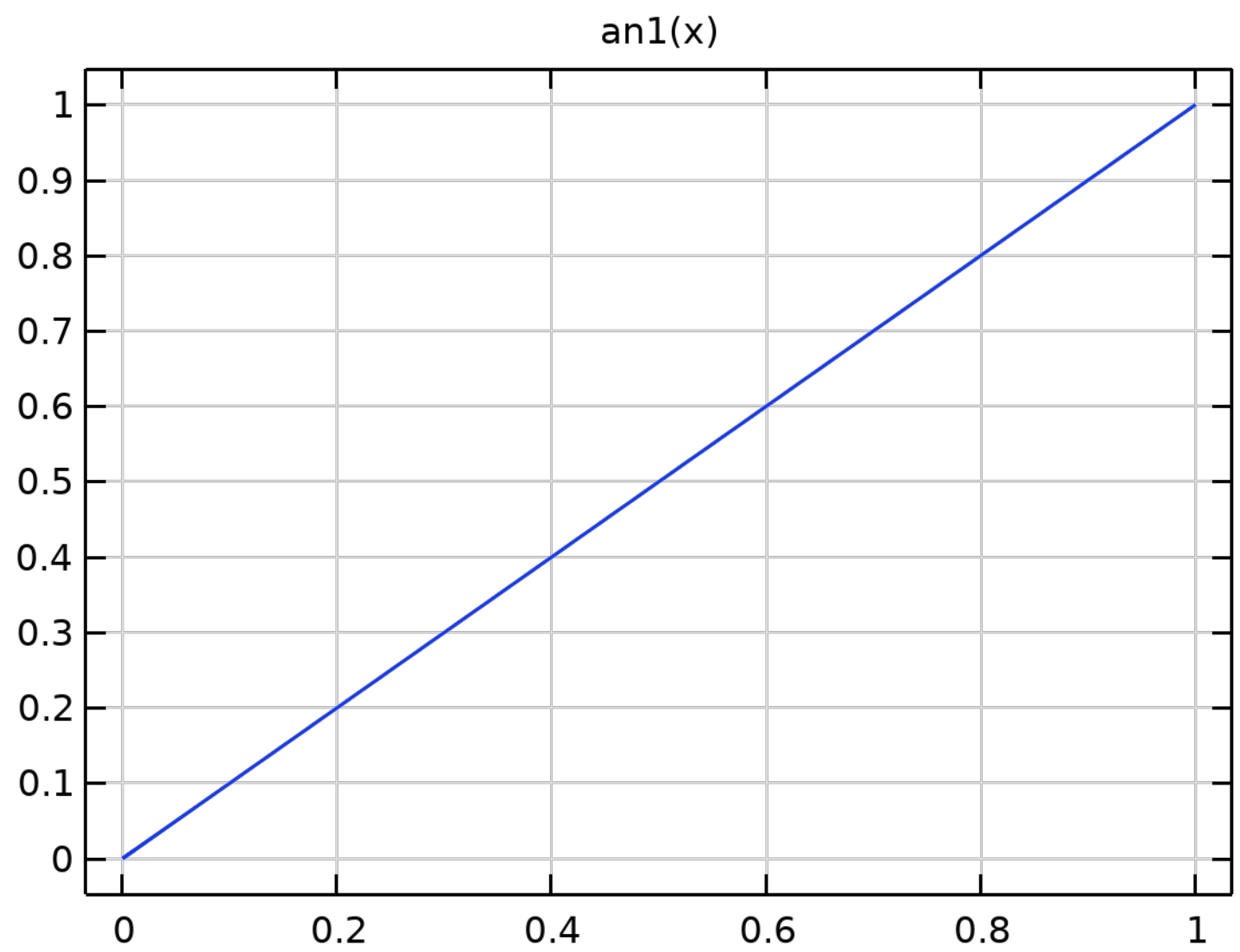

Analytic 1

DEFINITION

Description	Value
Expression	x
Arguments	x

UNITS

Argument	Unit
x	

2.2 GEOMETRY 1

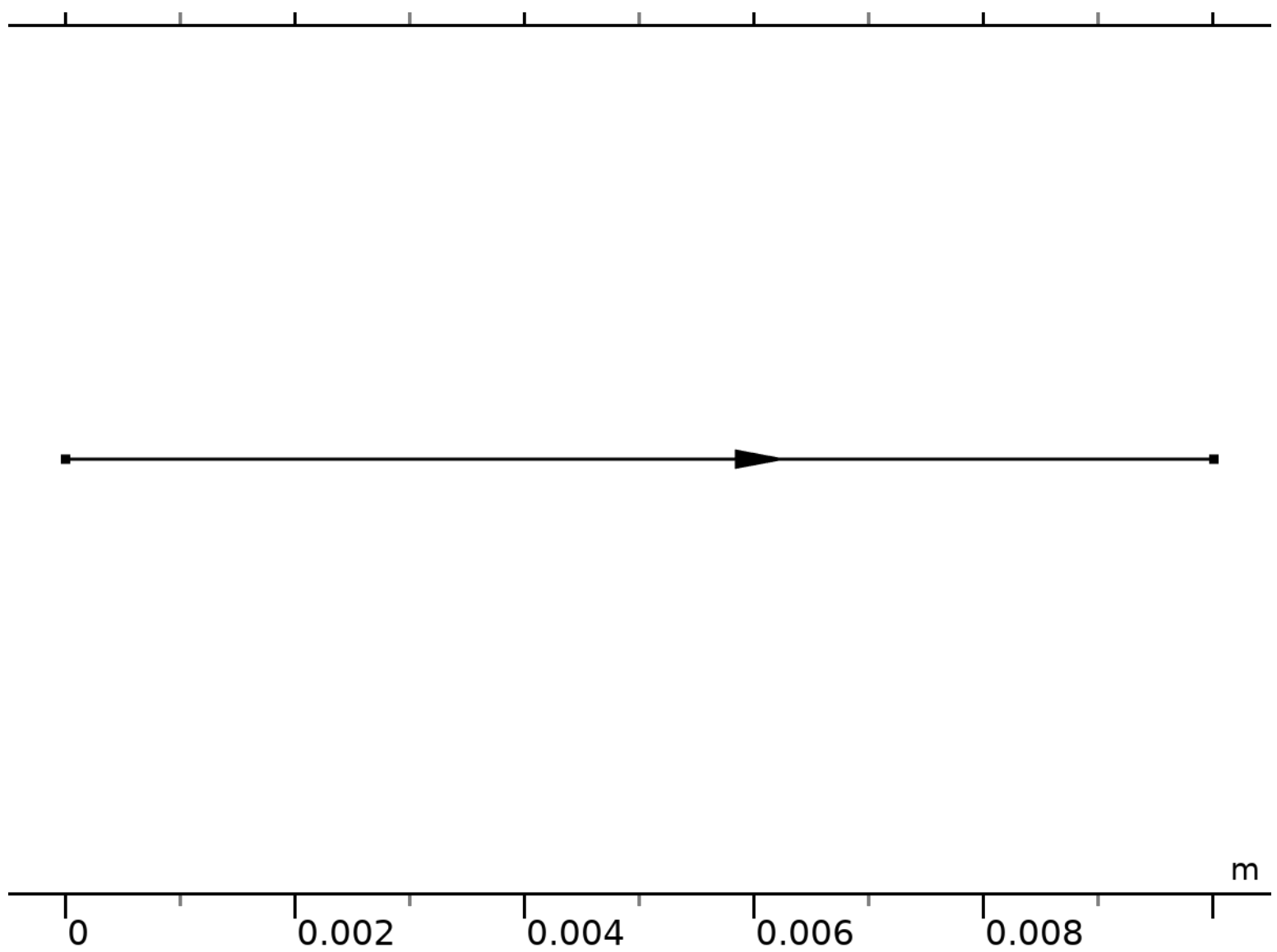

Geometry 1

UNITS

Length unit	m
Angular unit	deg

GEOMETRY STATISTICS

Description	Value
Space dimension	1
Number of domains	1
Number of boundaries	2

2.2.1 Interval 1 (i1)

INTERVAL

Coordinates (m)
0
1[cm]

INFORMATION

Description	Value
Last build time	Unknown

2.2.2 Form Union (fin)

INFORMATION

Description	Value
Details	{Formed union of 1 solid object., Union has 1 domain and 2 boundaries.}
Last build time	< 1 second
Built with	COMSOL 6.2.0.339 (win64), Oct 18, 2024, 12:43:28 PM

2.3 TRANSPORT OF DILUTED SPECIES

USED PRODUCTS

Chemical Reaction Engineering Module
COMSOL Multiphysics

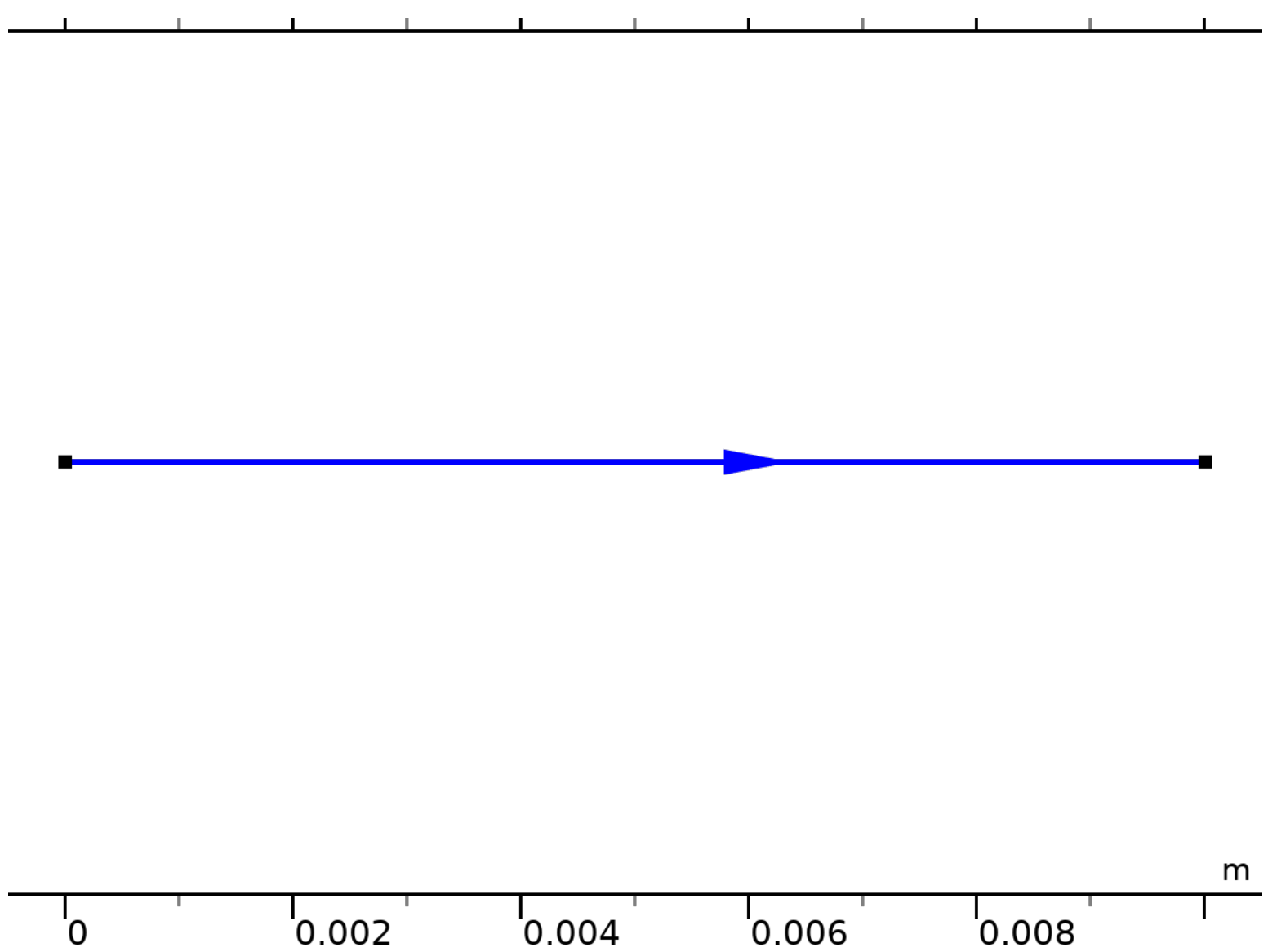

Transport of Diluted Species

SELECTION

Geometric entity level	Domain
Selection	Geometry geom1: Dimension 1: All domains

EQUATIONS

$$\frac{\partial c_i}{\partial t} + \nabla \cdot \mathbf{J}_i = R_i$$

$$\mathbf{J}_i = -D_i \nabla c_i$$

2.3.1 Interface Settings

Discretization

SETTINGS

Description	Value
Concentration	Linear
Compute boundary fluxes	On
Apply smoothing to boundary fluxes	On
Value type when using splitting of complex variables	Real

SETTINGS

Description	Value
Equation form	Study controlled

Cross-Sectional Area

SETTINGS

Description	Value	Unit
Cross-sectional area	1	m^2

Species Activity

SETTINGS

Description	Value
Species activity	Ideal

Advanced Settings

SETTINGS

Description	Value
Convective term	Nonconservative form

Transport Mechanisms

SETTINGS

Description	Value
Convection	Off
Migration in electric field	Off
Mass transfer in porous media	Off

2.3.2 Variables

Name	Expression	Unit	Description	Selection	Details
tds.Ac	1[m^2]	m^2	Cross-sectional area	Global	
tds.d	tds.Ac	m^2	Out-of-plane geometry extension	Global	
tds.f_H	1	1	Activity coefficient	Domain 1	

Name	Expression	Unit	Description	Selection	Details
tds.f_OH	1	1	Activity coefficient	Domain 1	
tds.nx	unx	1	Normal vector, x-component	Boundary 1	
tds.ny	0	1	Normal vector, y-component	Boundary 1	
tds.nz	0	1	Normal vector, z-component	Boundary 1	
tds.nx	dnx	1	Normal vector, x-component	Boundary 2	
tds.ny	0	1	Normal vector, y-component	Boundary 2	
tds.nz	0	1	Normal vector, z-component	Boundary 2	
tds.nX	unX	1	Normal vector, X-component	Boundary 1	
tds.nY	0	1	Normal vector, Y-component	Boundary 1	
tds.nZ	0	1	Normal vector, Z-component	Boundary 1	
tds.nX	dnX	1	Normal vector, X-component	Boundary 2	
tds.nY	0	1	Normal vector, Y-component	Boundary 2	
tds.nZ	0	1	Normal vector, Z-component	Boundary 2	
tds.nXg	unXg	1	Normal vector, Xg-component	Boundary 1	
tds.nYg	0	1	Normal vector, Yg-component	Boundary 1	
tds.nZg	0	1	Normal vector, Zg-component	Boundary 1	
tds.nXg	dnXg	1	Normal vector, Xg-component	Boundary 2	
tds.nYg	0	1	Normal vector, Yg-component	Boundary 2	
tds.nZg	0	1	Normal vector, Zg-component	Boundary 2	
tds.nxmesh	unxmesh	1	Normal vector (mesh), x-component	Boundary 1	

Name	Expression	Unit	Description	Selection	Details
tds.nymesh	0	1	Normal vector (mesh), y-component	Boundary 1	
tds.nzmesh	0	1	Normal vector (mesh), z-component	Boundary 1	
tds.nxmesh	dnxmesh	1	Normal vector (mesh), x-component	Boundary 2	
tds.nymesh	0	1	Normal vector (mesh), y-component	Boundary 2	
tds.nzmesh	0	1	Normal vector (mesh), z-component	Boundary 2	
tds.nxc	-nxc/tds.ncLen	1	Normal vector, x-component	Boundary 1	
tds.nyc	0	1	Normal vector, y-component	Boundary 1	
tds.nzc	0	1	Normal vector, z-component	Boundary 1	
tds.nxc	nxc/tds.ncLen	1	Normal vector, x-component	Boundary 2	
tds.nyc	0	1	Normal vector, y-component	Boundary 2	
tds.nzc	0	1	Normal vector, z-component	Boundary 2	
tds.R_H	0	mol/(m³·s)	Total rate expression	Domain 1	+ operation
tds.cP_H	0	mol/kg	Concentration species adsorbed to the solid	Domain 1	+ operation
tds.cP_H	0	mol/kg	Concentration species adsorbed to the solid	Boundaries 1–2	+ operation
tds.KP_H	0	m³/kg	Adsorption isotherm, first concentration derivative	Domain 1	+ operation
tds.KP_H	0	m³/kg	Adsorption isotherm, first concentration derivative	Boundaries 1–2	+ operation
tds.Rads_H	0	mol/(m³·s)	Total adsorption rate	Domain 1	+ operation
tds.DiT_H	0	m²/s	Turbulent diffusivity	Domain 1	
tds.cVar_H	H	mol/m³	Species	Boundaries 1–2	
tds.R_OH	0	mol/(m³·s)	Total rate expression	Domain 1	+ operation

Name	Expression	Unit	Description	Selection	Details
tds.cP_OH	0	mol/kg	Concentration species adsorbed to the solid	Domain 1	+ operation
tds.cP_OH	0	mol/kg	Concentration species adsorbed to the solid	Boundaries 1–2	+ operation
tds.KP_OH	0	m^3/kg	Adsorption isotherm, first concentration derivative	Domain 1	+ operation
tds.KP_OH	0	m^3/kg	Adsorption isotherm, first concentration derivative	Boundaries 1–2	+ operation
tds.Rads_OH	0	mol/(m^3·s)	Total adsorption rate	Domain 1	+ operation
tds.DiT_OH	0	m^2/s	Turbulent diffusivity	Domain 1	
tds.cVar_OH	OH	mol/m^3	Species	Boundaries 1–2	
tds.poro	1	1	Porosity	Domain 1	
tds.theta_g	0	1	Gas volume fraction	Domain 1	
tds.theta_l	1	1	Liquid volume fraction	Domain 1	
tds.theta	tds.poro	1	Mobile fluid volume fraction	Domain 1	

2.3.3 Transport Properties 1

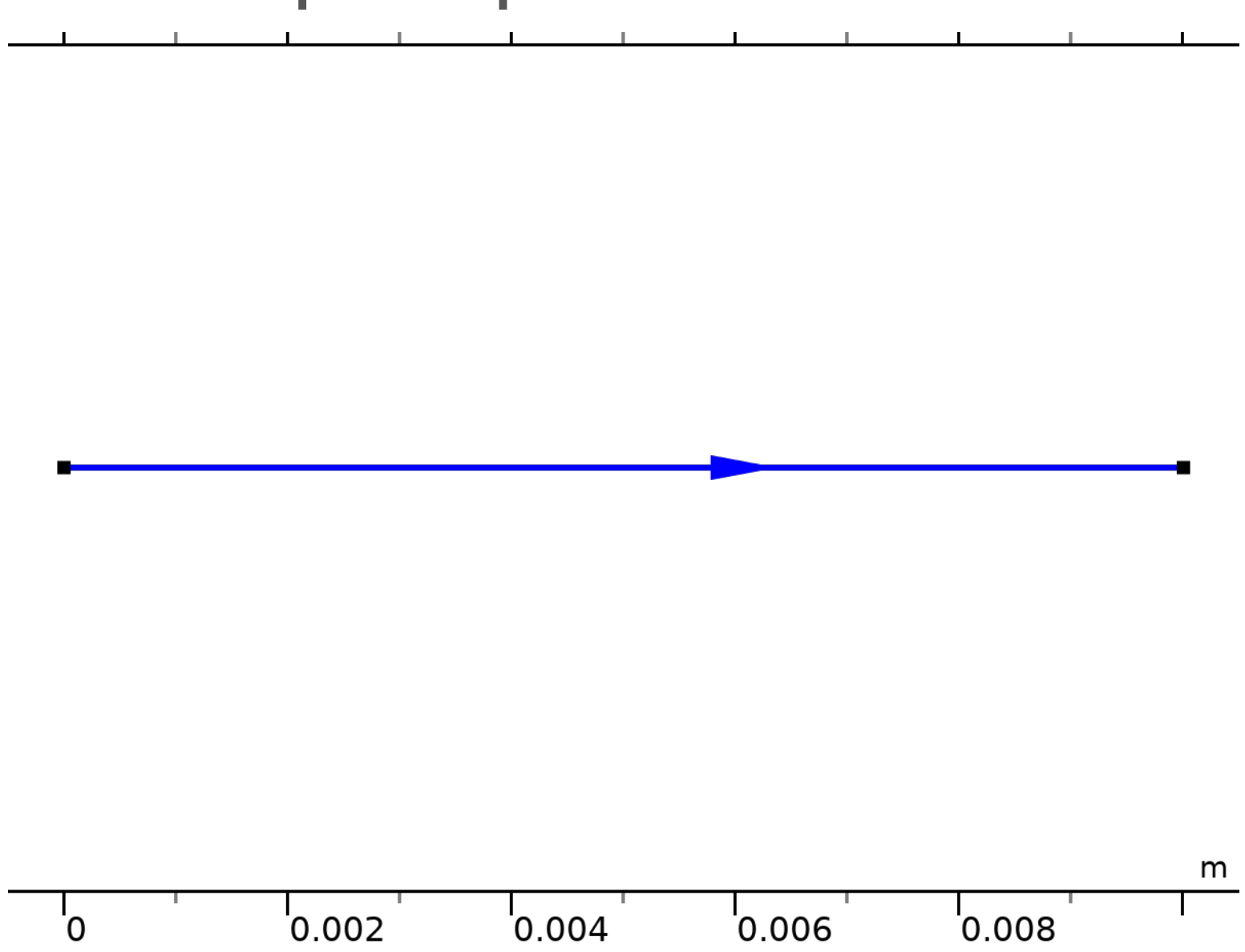

Transport Properties 1

SELECTION

Geometric entity level	Domain

Selection	Geometry geom1: Dimension 1: All domains

EQUATIONS

$$\nabla \cdot \mathbf{J}_i = R_i$$
$$\mathbf{J}_i = -D_i \nabla c_i$$

Diffusion

SETTINGS

Description	Value	Unit
Source	Material	
Material	None	
Diffusion coefficient	User defined	
Diffusion coefficient	D_Hsol	m^2/s
Diffusion coefficient	User defined	
Diffusion coefficient	D_OHsol	m^2/s

Coordinate System Selection

SETTINGS

Description	Value
Coordinate system	Global coordinate system

Model Input

SETTINGS

Description	Value	Unit
Temperature	User defined	
Temperature	Temp	K

Variables

Name	Expression	Unit	Description	Selection	Details
domflux.Hx	tds.dflux_Hx*tds.d	mol/s	Domain flux, x-component	Domain 1	
domflux.OHx	tds.dflux_OHx*tds.d	mol/s	Domain flux, x-component	Domain 1	
tds.ndflux_H	tds.bndFlux_H	$mol/(m^2 \cdot s)$	Normal diffusive flux	Boundaries 1–2	
tds.ntflux_H	tds.bndFlux_H	$mol/(m^2 \cdot s)$	Normal total flux	Boundaries 1–2	
tds.ndflux_OH	tds.bndFlux_OH	$mol/(m^2 \cdot s)$	Normal diffusive flux	Boundaries 1–2	

Name	Expression	Unit	Description	Selection	Details
tds.ntflux_OH	tds.bndFlux_OH	mol/(m²·s)	Normal total flux	Boundaries 1–2	
tds.DF_Hxx	D_Hsol	m²/s	Fluid diffusion coefficient, xx-component	Domain 1	
tds.DF_Hyx	0	m²/s	Fluid diffusion coefficient, yx-component	Domain 1	
tds.DF_Hzx	0	m²/s	Fluid diffusion coefficient, zx-component	Domain 1	
tds.DF_Hxy	0	m²/s	Fluid diffusion coefficient, xy-component	Domain 1	
tds.DF_Hyy	D_Hsol	m²/s	Fluid diffusion coefficient, yy-component	Domain 1	
tds.DF_Hzy	0	m²/s	Fluid diffusion coefficient, zy-component	Domain 1	
tds.DF_Hxz	0	m²/s	Fluid diffusion coefficient, xz-component	Domain 1	
tds.DF_Hyz	0	m²/s	Fluid diffusion coefficient, yz-component	Domain 1	
tds.DF_Hzz	D_Hsol	m²/s	Fluid diffusion coefficient, zz-component	Domain 1	
tds.D_Hxx	tds.DF_Hxx+tds.DiT_H	m²/s	Diffusion coefficient, xx-component	Domain 1	+ operation
tds.D_Hyx	tds.DF_Hyx	m²/s	Diffusion coefficient, yx-component	Domain 1	+ operation
tds.D_Hzx	tds.DF_Hzx	m²/s	Diffusion coefficient, zx-component	Domain 1	+ operation
tds.D_Hxy	tds.DF_Hxy	m²/s	Diffusion coefficient, xy-component	Domain 1	+ operation

Name	Expression	Unit	Description	Selection	Details
tds.D_Hyy	tds.DF_Hyy+tds.DiT_H	m²/s	Diffusion coefficient, yy-component	Domain 1	+ operation
tds.D_Hzy	tds.DF_Hzy	m²/s	Diffusion coefficient, zy-component	Domain 1	+ operation
tds.D_Hxz	tds.DF_Hxz	m²/s	Diffusion coefficient, xz-component	Domain 1	+ operation
tds.D_Hyz	tds.DF_Hyz	m²/s	Diffusion coefficient, yz-component	Domain 1	+ operation
tds.D_Hzz	tds.DF_Hzz+tds.DiT_H	m²/s	Diffusion coefficient, zz-component	Domain 1	+ operation
tds.DF_OHxx	D_OHsol	m²/s	Fluid diffusion coefficient, xx-component	Domain 1	
tds.DF_OHyx	0	m²/s	Fluid diffusion coefficient, yx-component	Domain 1	
tds.DF_OHzx	0	m²/s	Fluid diffusion coefficient, zx-component	Domain 1	
tds.DF_OHxy	0	m²/s	Fluid diffusion coefficient, xy-component	Domain 1	
tds.DF_OHyy	D_OHsol	m²/s	Fluid diffusion coefficient, yy-component	Domain 1	
tds.DF_OHzy	0	m²/s	Fluid diffusion coefficient, zy-component	Domain 1	
tds.DF_OHxz	0	m²/s	Fluid diffusion coefficient, xz-component	Domain 1	
tds.DF_OHyz	0	m²/s	Fluid diffusion coefficient, yz-component	Domain 1	
tds.DF_OHzz	D_OHsol	m²/s	Fluid diffusion coefficient, zz-component	Domain 1	

Name	Expression	Unit	Description	Selection	Details
tds.D_OHxx	tds.DF_OHxx+tds.DiT_OH	m^2/s	Diffusion coefficient, xx-component	Domain 1	+ operation
tds.D_OHyx	tds.DF_OHyx	m^2/s	Diffusion coefficient, yx-component	Domain 1	+ operation
tds.D_OHzx	tds.DF_OHzx	m^2/s	Diffusion coefficient, zx-component	Domain 1	+ operation
tds.D_OHxy	tds.DF_OHxy	m^2/s	Diffusion coefficient, xy-component	Domain 1	+ operation
tds.D_OHyy	tds.DF_OHyy+tds.DiT_OH	m^2/s	Diffusion coefficient, yy-component	Domain 1	+ operation
tds.D_OHzy	tds.DF_OHzy	m^2/s	Diffusion coefficient, zy-component	Domain 1	+ operation
tds.D_OHxz	tds.DF_OHxz	m^2/s	Diffusion coefficient, xz-component	Domain 1	+ operation
tds.D_OHyz	tds.DF_OHyz	m^2/s	Diffusion coefficient, yz-component	Domain 1	+ operation
tds.D_OHzz	tds.DF_OHzz+tds.DiT_OH	m^2/s	Diffusion coefficient, zz-component	Domain 1	+ operation
tds.Dav_H	tds.D_Hxx	m^2/s	Average diffusion coefficient	Domain 1	
tds.Dav_OH	tds.D_OHxx	m^2/s	Average diffusion coefficient	Domain 1	
tds.tflux_Hx	tds.dflux_Hx	$mol/(m^2 \cdot s)$	Total flux, x-component	Domain 1	+ operation
tds.tflux_Hy	tds.dflux_Hy	$mol/(m^2 \cdot s)$	Total flux, y-component	Domain 1	+ operation
tds.tflux_Hz	tds.dflux_Hz	$mol/(m^2 \cdot s)$	Total flux, z-component	Domain 1	+ operation
tds.dfluxMag_H	sqrt(tds.dflux_Hx^2+tds.dflux_Hy^2+tds.dflux_Hz^2)	$mol/(m^2 \cdot s)$	Diffusive flux magnitude	Domain 1	

Name	Expression	Unit	Description	Selection	Details
tds.tfluxMag_H	sqrt(tds.tflux_Hx^2+tds.tflux_Hy^2+tds.tflux_Hz^2)	mol/(m²·s)	Total flux magnitude	Domain 1	
tds.dpflux_Hx	0	mol/(m²·s)	Dispersive flux, x-component	Domain 1	
tds.dpflux_Hy	0	mol/(m²·s)	Dispersive flux, y-component	Domain 1	
tds.dpflux_Hz	0	mol/(m²·s)	Dispersive flux, z-component	Domain 1	
tds.tflux_OHx	tds.dflux_OHx	mol/(m²·s)	Total flux, x-component	Domain 1	+ operation
tds.tflux_OHy	tds.dflux_OHy	mol/(m²·s)	Total flux, y-component	Domain 1	+ operation
tds.tflux_OHz	tds.dflux_OHz	mol/(m²·s)	Total flux, z-component	Domain 1	+ operation
tds.dfluxMag_OH	sqrt(tds.dflux_OHx^2+tds.dflux_OHy^2+tds.dflux_OHz^2)	mol/(m²·s)	Diffusive flux magnitude	Domain 1	
tds.tfluxMag_OH	sqrt(tds.tflux_OHx^2+tds.tflux_OHy^2+tds.tflux_OHz^2)	mol/(m²·s)	Total flux magnitude	Domain 1	
tds.dpflux_OHx	0	mol/(m²·s)	Dispersive flux, x-component	Domain 1	
tds.dpflux_OHy	0	mol/(m²·s)	Dispersive flux, y-component	Domain 1	
tds.dpflux_OHz	0	mol/(m²·s)	Dispersive flux, z-component	Domain 1	
tds.H_material	H*spatial.detF	mol/m³	Concentration	Domain 1	
tds.dflux_Hx	-tds.D_Hxx*Hx	mol/(m²·s)	Diffusive flux, x-component	Domain 1	+ operation
tds.dflux_Hy	-tds.D_Hyx*Hx	mol/(m²·s)	Diffusive flux, y-component	Domain 1	+ operation
tds.dflux_Hz	-tds.D_Hzx*Hx	mol/(m²·s)	Diffusive flux, z-component	Domain 1	+ operation
tds.grad_Hx	Hx	mol/m⁴	Concentration gradient, x-component	Domain 1	

Name	Expression	Unit	Description	Selection	Details
tds.grad_Hy	0	mol/m⁴	Concentration gradient, y-component	Domain 1	
tds.grad_Hz	0	mol/m⁴	Concentration gradient, z-component	Domain 1	
tds.OH_material	OH*spatial.detF	mol/m³	Concentration	Domain 1	
tds.dflux_OHx	-tds.D_OHxx*OHx	mol/(m²·s)	Diffusive flux, x-component	Domain 1	+ operation
tds.dflux_OHy	-tds.D_OHyx*OHx	mol/(m²·s)	Diffusive flux, y-component	Domain 1	+ operation
tds.dflux_OHz	-tds.D_OHzx*OHx	mol/(m²·s)	Diffusive flux, z-component	Domain 1	+ operation
tds.grad_OHx	OHx	mol/m⁴	Concentration gradient, x-component	Domain 1	
tds.grad_OHy	0	mol/m⁴	Concentration gradient, y-component	Domain 1	
tds.grad_OHz	0	mol/m⁴	Concentration gradient, z-component	Domain 1	
tds.bndFlux_H	-uflux_spatial(H)/tds.d	mol/(m²·s)	Boundary flux	Boundary 1	Meta
tds.bndFlux_H	-dflux_spatial(H)/tds.d	mol/(m²·s)	Boundary flux	Boundary 2	Meta
tds.bndFlux_OH	-uflux_spatial(OH)/tds.d	mol/(m²·s)	Boundary flux	Boundary 1	Meta
tds.bndFlux_OH	-dflux_spatial(OH)/tds.d	mol/(m²·s)	Boundary flux	Boundary 2	Meta
tds.Rlin_H	0	1/s	Linear source term coefficient	Domain 1	+ operation
tds.Res_H	-H*tds.Rlin_H-tds.R_H	mol/(m³·s)	Equation residual	Domain 1	
tds.Rlin_OH	0	1/s	Linear source term coefficient	Domain 1	+ operation
tds.Res_OH	-OH*tds.Rlin_OH-tds.R_OH	mol/(m³·s)	Equation residual	Domain 1	

Shape functions

Name	Shape function	Unit	Description	Shape frame	Selection
H	Lagrange (Linear)	mol/m^3	Concentration	Spatial	Domain 1
OH	Lagrange (Linear)	mol/m^3	Concentration	Spatial	Domain 1

Weak Expressions

Weak expression	Integration order	Integration frame	Selection
tds.dflux_Hx*test(Hx)*tds.d	2	Spatial	Domain 1
tds.dflux_OHx*test(OHx)*tds.d	2	Spatial	Domain 1
tds.streamline*(isScalingSystemDomain==0)*tds.d	2	Spatial	Domain 1
tds.crosswind*(isScalingSystemDomain==0)*tds.d	4	Spatial	Domain 1

2.3.4 No Flux 1

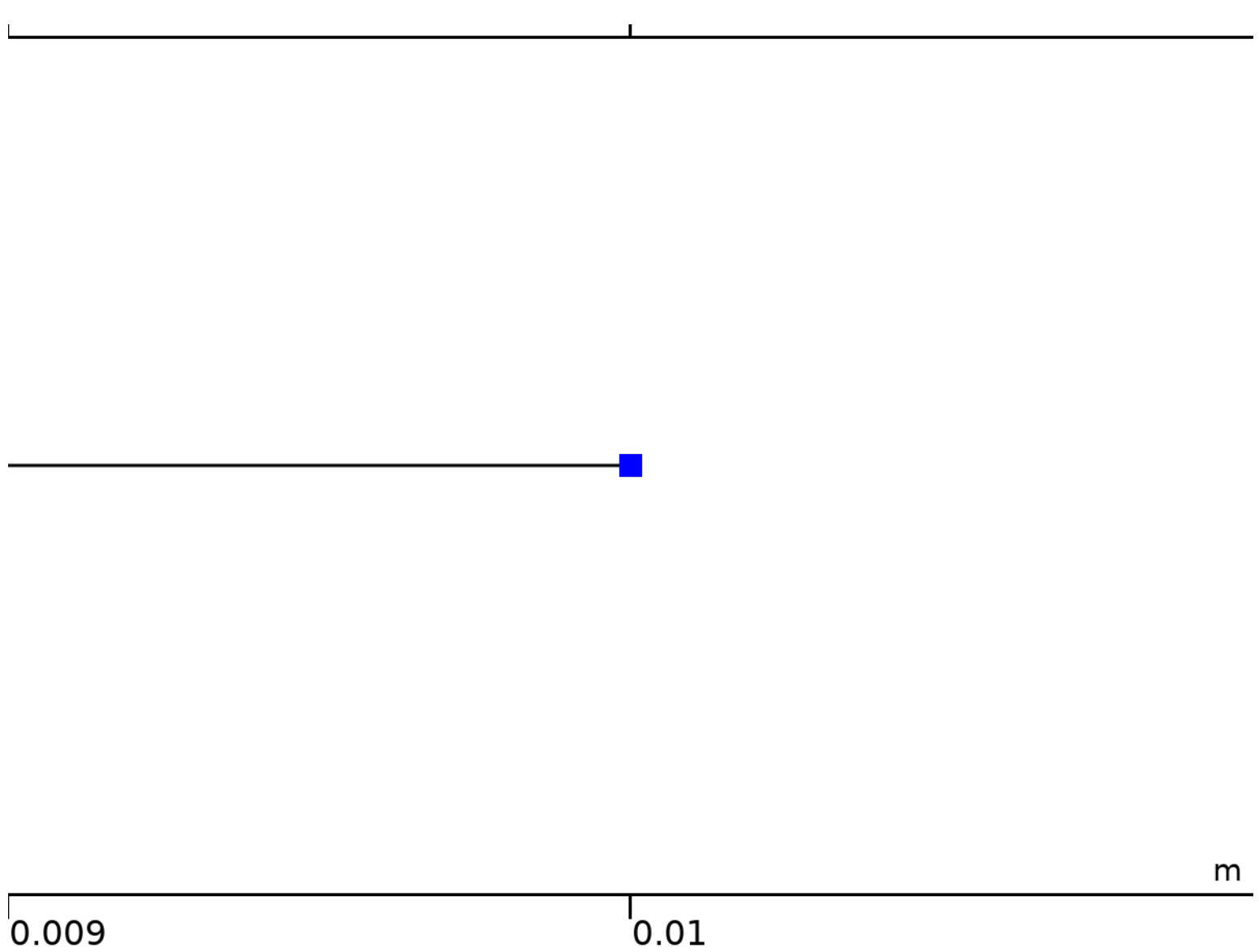

No Flux 1

SELECTION

Geometric entity level	Boundary
Selection	Geometry geom1: Dimension 0: All boundaries

EQUATIONS

$$-\mathbf{n}\cdot\mathbf{J}_i=0$$

2.3.5 Initial Values 1

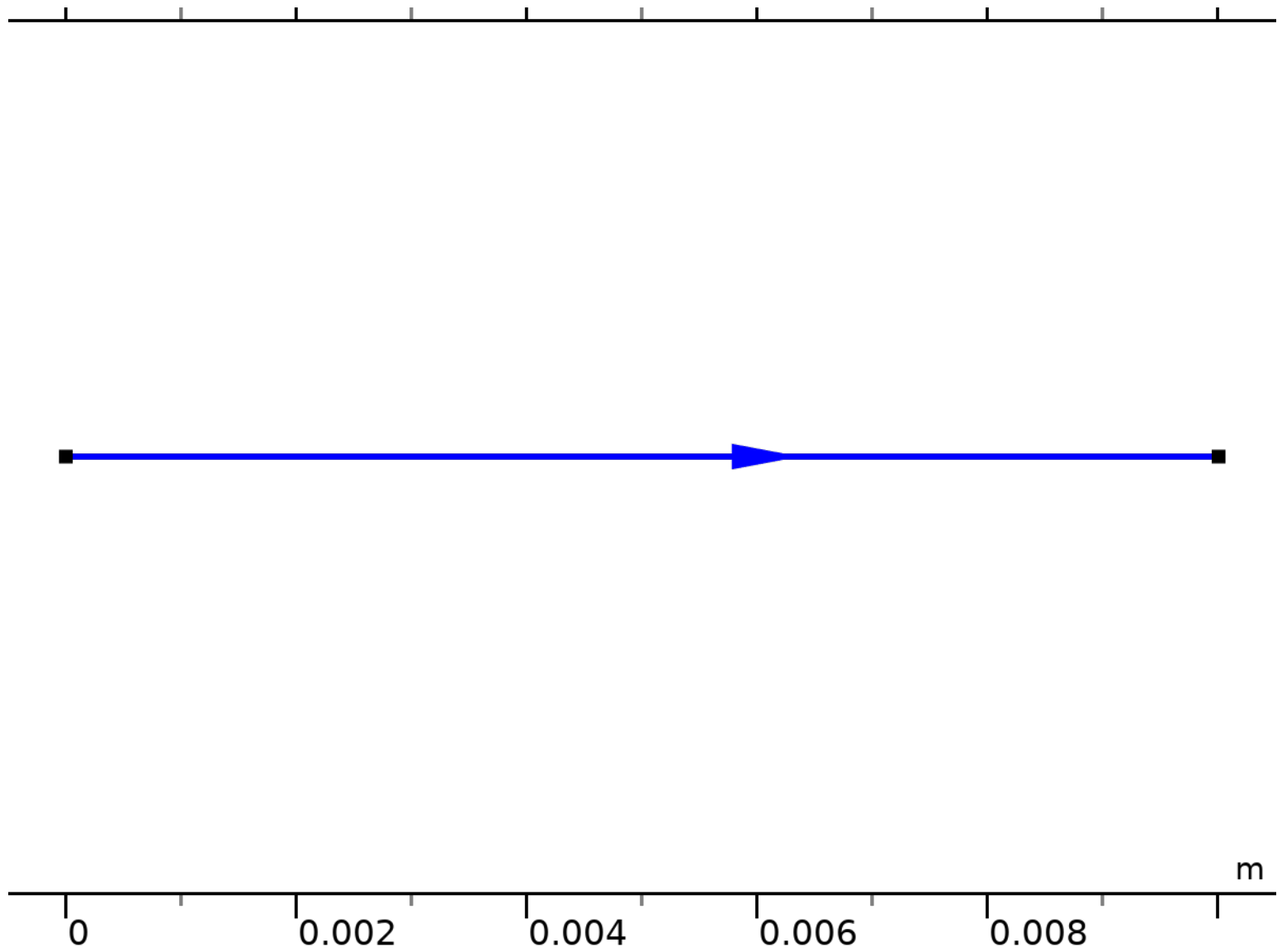

Initial Values 1

SELECTION

Geometric entity level	Domain
Selection	Geometry geom1: Dimension 1: All domains

Initial Values

SETTINGS

Description	Value	Unit
Concentration	{H_bulk, OH_bulk}	mol/m³

Variables

Name	Expression	Unit	Description	Selection	Details
tds.c0_H	H_bulk	mol/m³	Concentration	Domain 1	+ operation
tds.c0_OH	OH_bulk	mol/m³	Concentration	Domain 1	+ operation

2.3.6 Flux 1

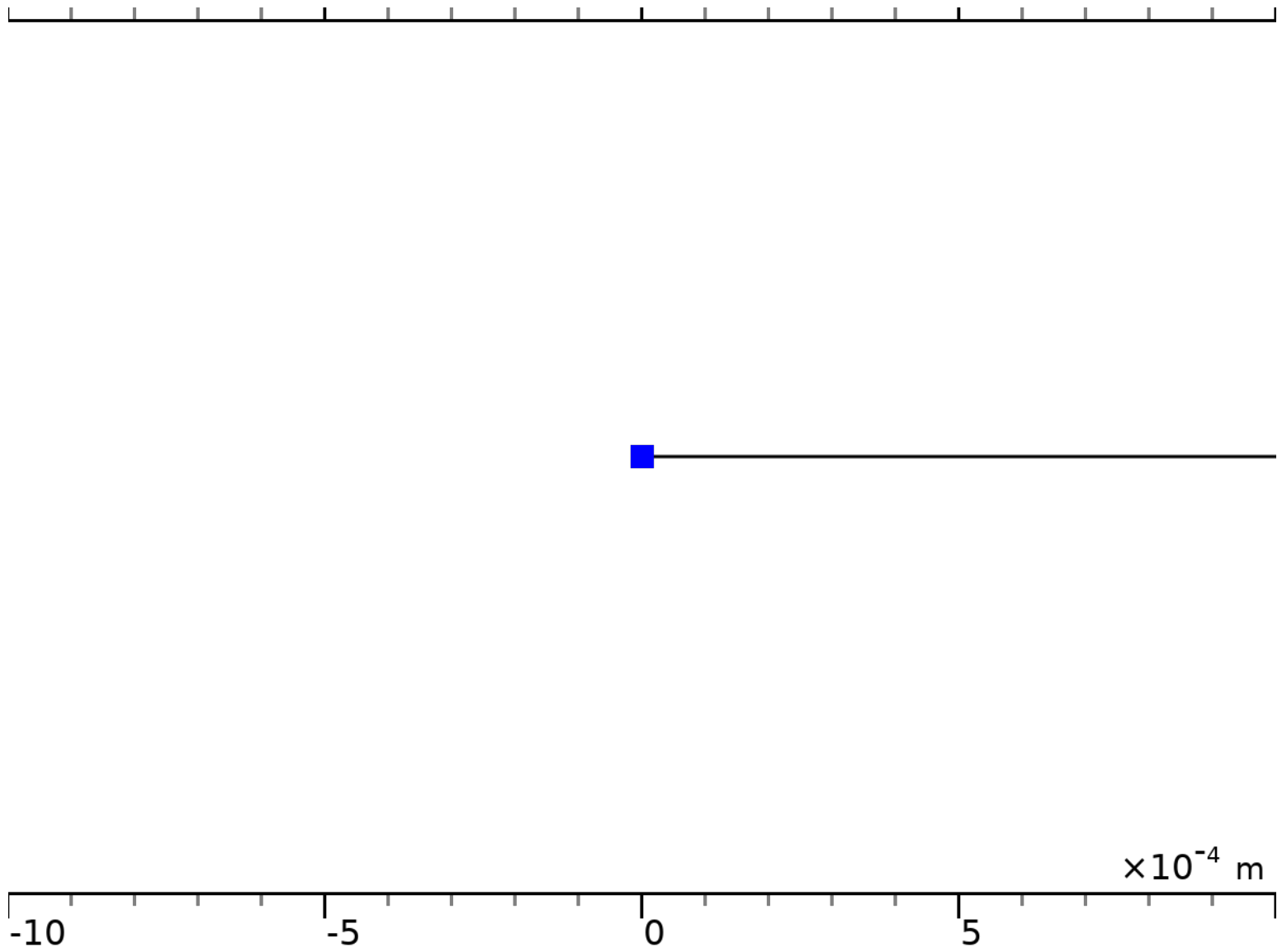

Flux 1

SELECTION

Geometric entity level	Boundary
Selection	Geometry geom1: Dimension 0: Boundary 1

EQUATIONS

$$-\mathbf{n}\cdot\mathbf{J}_i = J_{0,i}$$

Inward Flux

SETTINGS

Description	Value	Unit
Flux type	General inward flux	
Species H	On	
Species OH	Off	
	{-j_O_Proton - j_P_Proton - j_T_Proton - 2*(j_Q_Proton + j_R_Proton + j_V_Proton), 0}	mol/(m²·s)

Variables

Name	Expression	Unit	Description	Selection
tds.fl1.nmflow_H	tds.fl1.int(tds.ntflux_H)*tds.d	mol/s	Normal molar flow rate	Global
tds.fl1.nmflow_OH	tds.fl1.int(tds.ntflux_OH)*tds.d	mol/s	Normal molar flow rate	Global

Weak Expressions

Weak expression	Integration order	Integration frame	Selection
-(j_O_Proton+j_P_Proton+j_T_Proton+2*(j_Q_Proton+j_R_Proton+j_V_Proton))*test(H)*tds.d	2	Spatial	Boundary 1
0	2	Spatial	Boundary 1

2.4 SURFACE REACTIONS

USED PRODUCTS

Chemical Reaction Engineering Module
COMSOL Multiphysics

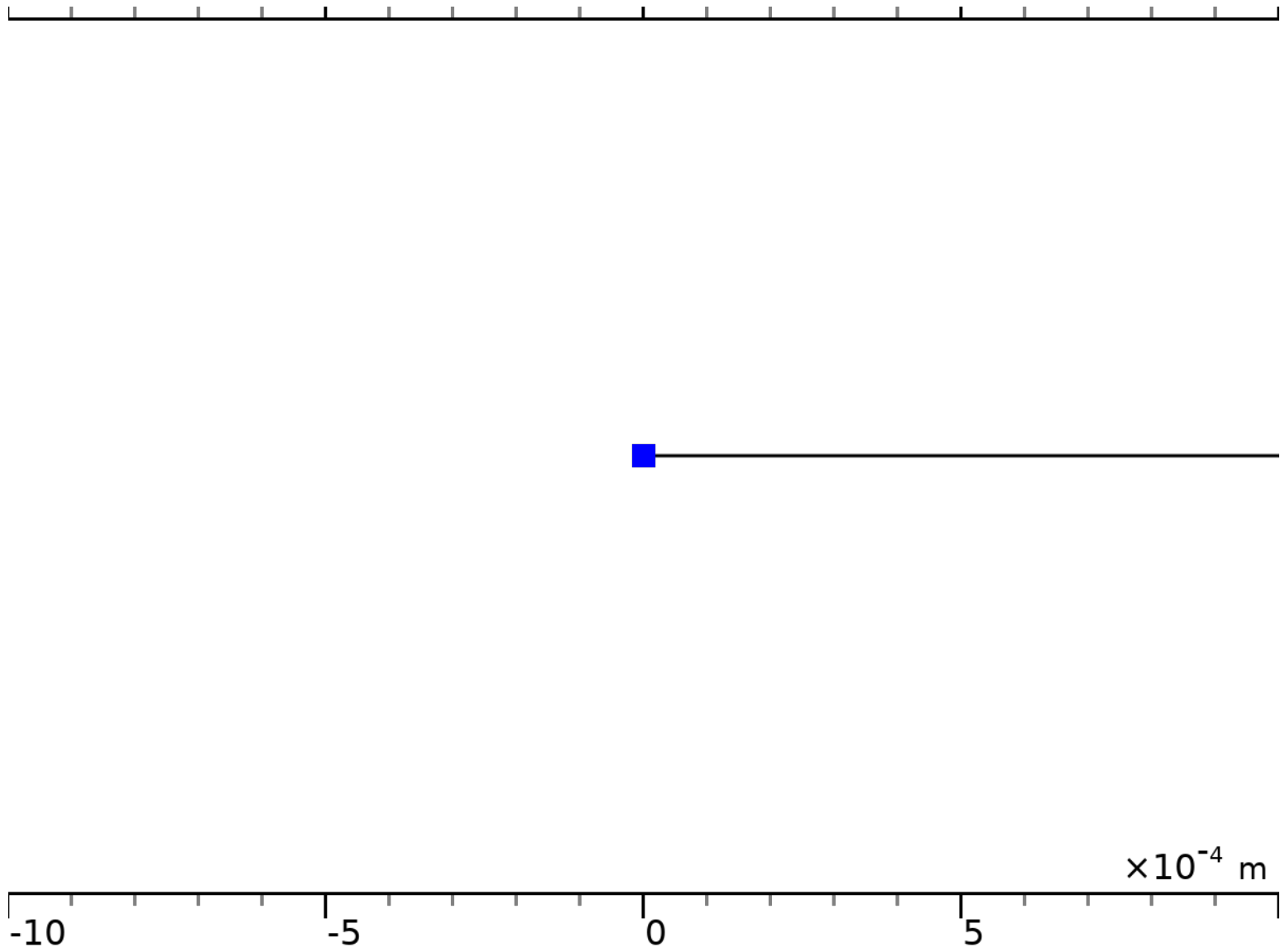

Surface Reactions

SELECTION

Geometric entity level	Boundary
Selection	Geometry geom1: Dimension 0: Boundary 1

EQUATIONS

$$\frac{\partial c_{s,i}}{\partial t} = R_{s,i}$$

$$\theta_i = \frac{c_{s,i}\sigma_i}{\Gamma_s}$$

$$\frac{\partial c_{b,i}}{\partial t} = R_{b,i}$$

2.4.1 Interface Settings

Discretization

SETTINGS

Description	Value
Surface concentration	Linear
Bulk concentration	Linear

SETTINGS

Description	Value
Equation form	Study controlled

2.4.2 Surface Properties 1

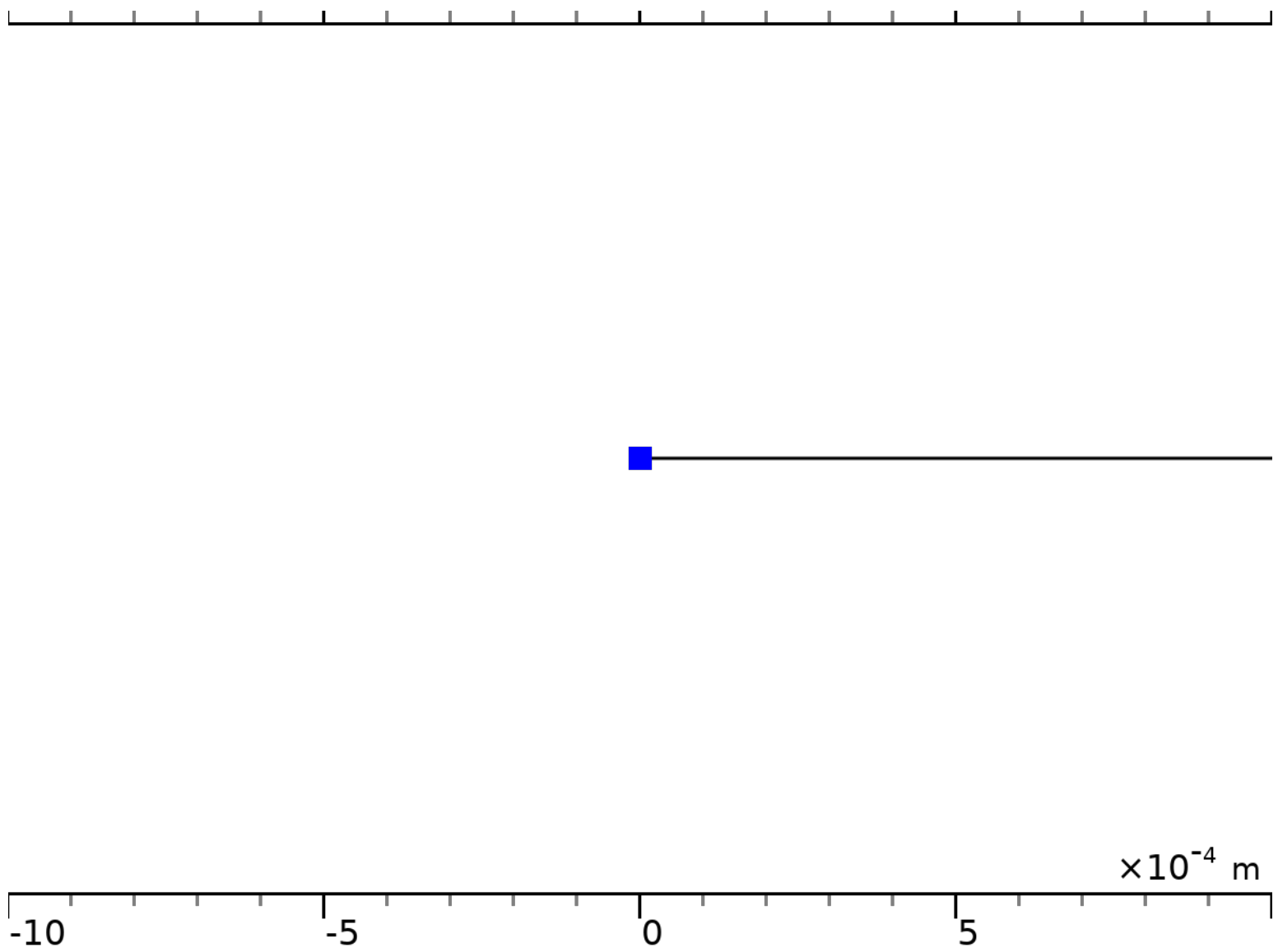

Surface Properties 1

SELECTION

Geometric entity level	Boundary
Selection	Geometry geom1: Dimension 0: All boundaries

EQUATIONS

$$\frac{\partial c_{s,j}}{\partial t} = R_{s,j}$$

$$\theta_i = \frac{c_{s,j}\sigma_i}{\Gamma_s}$$

Sites

SETTINGS

Description	Value	Unit
Density of sites	SiteDensity	mol/m^2
Site occupancy number	{1, 1, 1, 1, 1, 1, 1, 1, 1}	1

Variables

Name	Expression	Unit	Description	Selection	Details
sr.Rs_M	0	$mol/(m^2 \cdot s)$	Reaction rate for surface species	Boundary 1	+ operation
sr.Rs_O	0	$mol/(m^2 \cdot s)$	Reaction rate for surface species	Boundary 1	+ operation
sr.Rs_N	0	$mol/(m^2 \cdot s)$	Reaction rate for surface species	Boundary 1	+ operation
sr.Rs_P	0	$mol/(m^2 \cdot s)$	Reaction rate for surface species	Boundary 1	+ operation
sr.Rs_Q	0	$mol/(m^2 \cdot s)$	Reaction rate for surface species	Boundary 1	+ operation
sr.Rs_R	0	$mol/(m^2 \cdot s)$	Reaction rate for surface species	Boundary 1	+ operation
sr.Rs_S	0	$mol/(m^2 \cdot s)$	Reaction rate for surface species	Boundary 1	+ operation
sr.Rs_T	0	$mol/(m^2 \cdot s)$	Reaction rate for surface species	Boundary 1	+ operation
sr.Rs_V	0	$mol/(m^2 \cdot s)$	Reaction rate for surface species	Boundary 1	+ operation
sr.Gamma	SiteDensity	mol/m^2	Density of sites	Boundary 1	
sr.Sigma_M	1	1	Site occupancy number	Boundary 1	
sr.Sigma_O	1	1	Site occupancy number	Boundary 1	
sr.Sigma_N	1	1	Site occupancy number	Boundary 1	
sr.Sigma_P	1	1	Site occupancy number	Boundary 1	

Name	Expression	Unit	Description	Selection	Details
sr.Sigma_Q	1	1	Site occupancy number	Boundary 1	
sr.Sigma_R	1	1	Site occupancy number	Boundary 1	
sr.Sigma_S	1	1	Site occupancy number	Boundary 1	
sr.Sigma_T	1	1	Site occupancy number	Boundary 1	
sr.Sigma_V	1	1	Site occupancy number	Boundary 1	
sr.theta_i_M	max(eps^2,sr.Sigma_M*M/sr.Gamma)	1	Surface coverage	Boundary 1	
sr.theta_i0	sr.theta_i_M+sr.theta_i_O+sr.theta_i_N+sr.theta_i_P+sr.theta_i_Q+sr.theta_i_R+sr.theta_i_S+sr.theta_i_T+sr.theta_i_V	1	Surface coverage	Boundary 1	+ operation
sr.theta_i_O	max(eps^2,sr.Sigma_O*O/sr.Gamma)	1	Surface coverage	Boundary 1	
sr.theta_i_N	max(eps^2,sr.Sigma_N*N/sr.Gamma)	1	Surface coverage	Boundary 1	
sr.theta_i_P	max(eps^2,sr.Sigma_P*P/sr.Gamma)	1	Surface coverage	Boundary 1	
sr.theta_i_Q	max(eps^2,sr.Sigma_Q*Q/sr.Gamma)	1	Surface coverage	Boundary 1	
sr.theta_i_R	max(eps^2,sr.Sigma_R*R/sr.Gamma)	1	Surface coverage	Boundary 1	
sr.theta_i_S	max(eps^2,sr.Sigma_S*S/sr.Gamma)	1	Surface coverage	Boundary 1	
sr.theta_i_T	max(eps^2,sr.Sigma_T*T/sr.Gamma)	1	Surface coverage	Boundary 1	
sr.theta_i_V	max(eps^2,sr.Sigma_V*V/sr.Gamma)	1	Surface coverage	Boundary 1	
sr.theta_free	max(eps^2,min(1,1-sr.theta_i0))	1	Fraction of free sites	Boundary 1	

Shape functions

Name	Shape function	Unit	Description	Shape frame	Selection
M	Lagrange (Linear)	mol/m²	Surface concentration	Spatial	Boundary 1

Name	Shape function	Unit	Description	Shape frame	Selection
O	Lagrange (Linear)	mol/m²	Surface concentration	Spatial	Boundary 1
N	Lagrange (Linear)	mol/m²	Surface concentration	Spatial	Boundary 1
P	Lagrange (Linear)	mol/m²	Surface concentration	Spatial	Boundary 1
Q	Lagrange (Linear)	mol/m²	Surface concentration	Spatial	Boundary 1
R	Lagrange (Linear)	mol/m²	Surface concentration	Spatial	Boundary 1
S	Lagrange (Linear)	mol/m²	Surface concentration	Spatial	Boundary 1
T	Lagrange (Linear)	mol/m²	Surface concentration	Spatial	Boundary 1
V	Lagrange (Linear)	mol/m²	Surface concentration	Spatial	Boundary 1

Weak Expressions

Weak expression	Integration order	Integration frame	Selection
-d(M,t)*test(M)	2	Spatial	Boundary 1
-d(O,t)*test(O)	2	Spatial	Boundary 1
-d(N,t)*test(N)	2	Spatial	Boundary 1
-d(P,t)*test(P)	2	Spatial	Boundary 1
-d(Q,t)*test(Q)	2	Spatial	Boundary 1
-d(R,t)*test(R)	2	Spatial	Boundary 1
-d(S,t)*test(S)	2	Spatial	Boundary 1
-d(T,t)*test(T)	2	Spatial	Boundary 1
-d(V,t)*test(V)	2	Spatial	Boundary 1

2.4.3 Initial Values

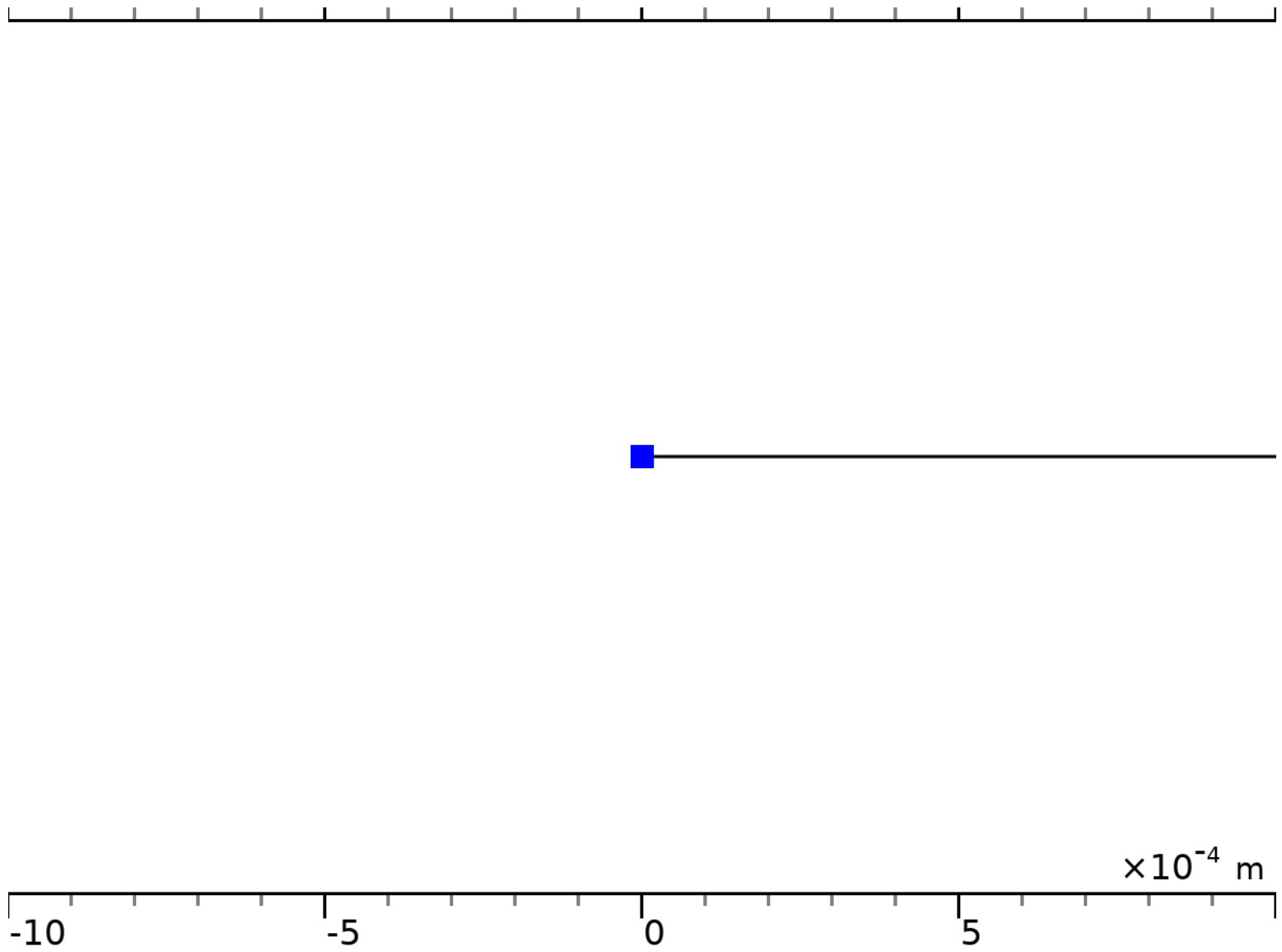

Initial Values

SELECTION

Geometric entity level	Boundary
Selection	Geometry geom1: Dimension 0: All boundaries

Initial Values

SETTINGS

Description	Value	Unit
Surface concentration	{SiteDensity, root.eps, root.eps, root.eps, root.eps, root.eps, root.eps, root.eps, root.eps}	mol/m²

2.4.4 Reactions 1

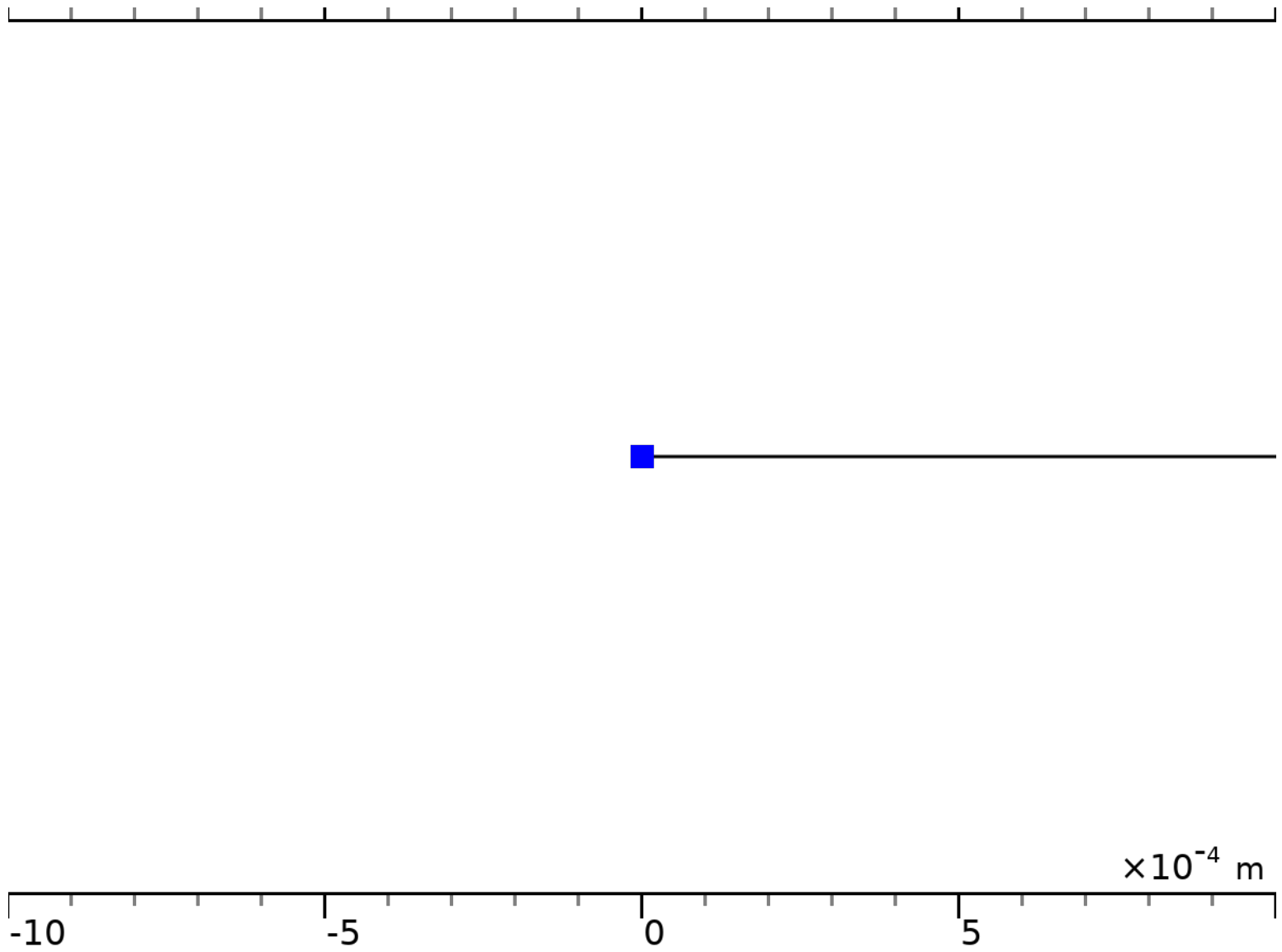

Reactions 1

SELECTION

Geometric entity level	Boundary
Selection	Geometry geom1: Dimension 0: Boundary 1

Reaction Rate for Surface Species

SETTINGS

**Description**	**Value**	**Unit**
Reaction rate for surface species	User defined	
Reaction rate for surface species	j_M	mol/(m²·s)
Reaction rate for surface species	User defined	
Reaction rate for surface species	j_O	mol/(m²·s)
Reaction rate for surface species	User defined	
Reaction rate for surface species	j_N	mol/(m²·s)
Reaction rate for surface species	User defined	
Reaction rate for surface species	j_P	mol/(m²·s)
Reaction rate for surface species	User defined	
Reaction rate for surface species	j_Q	mol/(m²·s)
Reaction rate for surface species	User defined	
Reaction rate for surface species	j_R	mol/(m²·s)
Reaction rate for surface species	User defined	
Reaction rate for surface species	j_S	mol/(m²·s)

Description	Value	Unit
Reaction rate for surface species	User defined	
Reaction rate for surface species	j_T	mol/(m²·s)
Reaction rate for surface species	User defined	
Reaction rate for surface species	j_V	mol/(m²·s)

Variables

Name	Expression	Unit	Description	Selection	Details
sr.Rs_M	model.input.Rs_M	mol/(m²·s)	Reaction rate for surface species	Boundary 1	Meta, + operation
sr.Rs_O	model.input.Rs_O	mol/(m²·s)	Reaction rate for surface species	Boundary 1	Meta, + operation
sr.Rs_N	model.input.Rs_N	mol/(m²·s)	Reaction rate for surface species	Boundary 1	Meta, + operation
sr.Rs_P	model.input.Rs_P	mol/(m²·s)	Reaction rate for surface species	Boundary 1	Meta, + operation
sr.Rs_Q	model.input.Rs_Q	mol/(m²·s)	Reaction rate for surface species	Boundary 1	Meta, + operation
sr.Rs_R	model.input.Rs_R	mol/(m²·s)	Reaction rate for surface species	Boundary 1	Meta, + operation
sr.Rs_S	model.input.Rs_S	mol/(m²·s)	Reaction rate for surface species	Boundary 1	Meta, + operation
sr.Rs_T	model.input.Rs_T	mol/(m²·s)	Reaction rate for surface species	Boundary 1	Meta, + operation
sr.Rs_V	model.input.Rs_V	mol/(m²·s)	Reaction rate for surface species	Boundary 1	Meta, + operation

Weak Expressions

Weak expression	Integration order	Integration frame	Selection
model.input.Rs_M*test(M)	2	Spatial	Boundary 1
model.input.Rs_O*test(O)	2	Spatial	Boundary 1
model.input.Rs_N*test(N)	2	Spatial	Boundary 1
model.input.Rs_P*test(P)	2	Spatial	Boundary 1

Weak expression	Integration order	Integration frame	Selection
model.input.Rs_Q*test(Q)	2	Spatial	Boundary 1
model.input.Rs_R*test(R)	2	Spatial	Boundary 1
model.input.Rs_S*test(S)	2	Spatial	Boundary 1
model.input.Rs_T*test(T)	2	Spatial	Boundary 1
model.input.Rs_V*test(V)	2	Spatial	Boundary 1

2.5 MESH 1

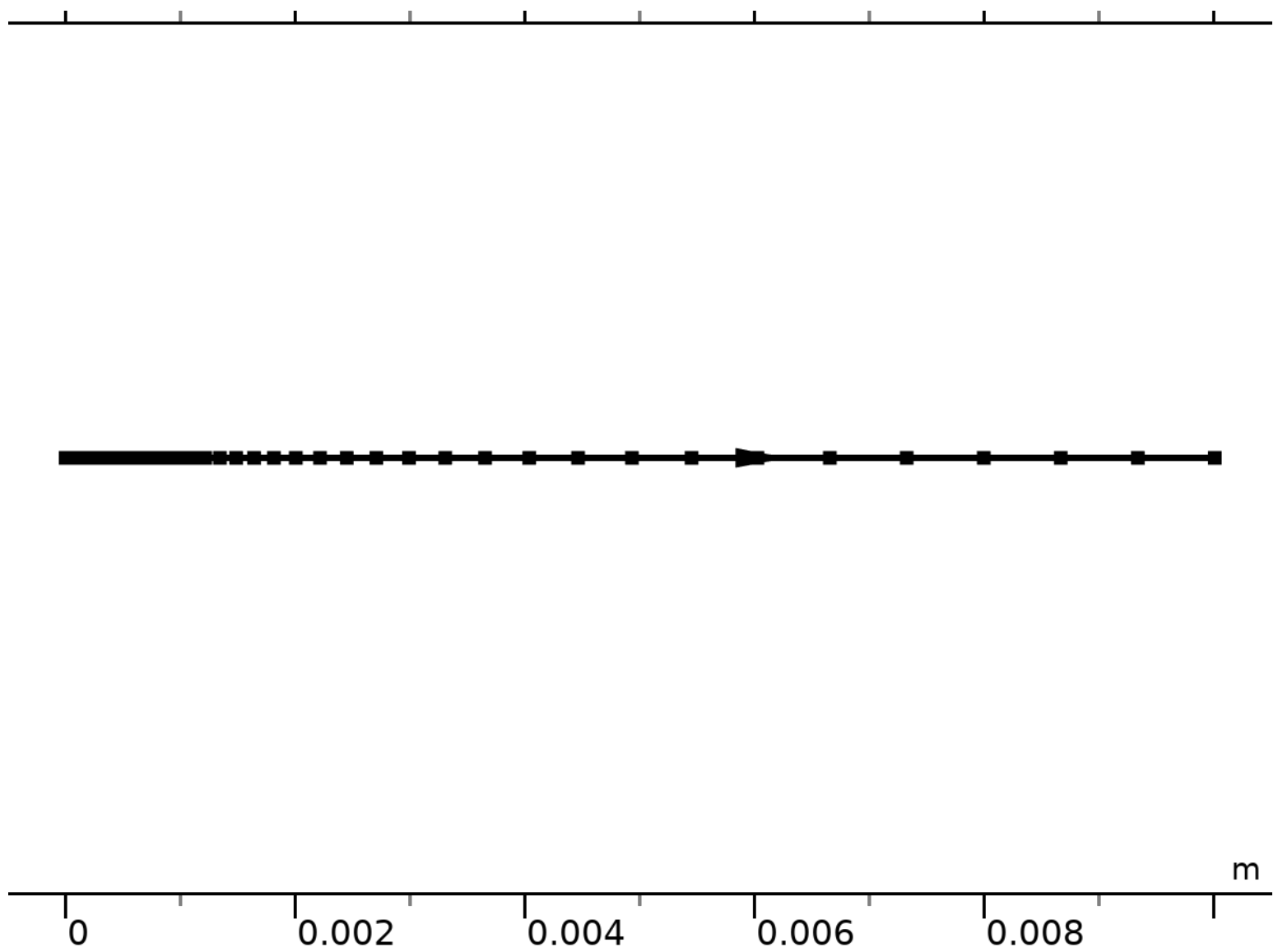

Mesh 1

2.5.1 Size (size)

SETTINGS

Description	Value
Maximum element size	6.7E-4
Minimum element size	3E-6
Curvature factor	0.3
Maximum element growth rate	1.3

2.5.2 Edge 1 (edg1)

SELECTION

Geometric entity level	Domain
Selection	Geometry geom1: Dimension 1: Domain 1

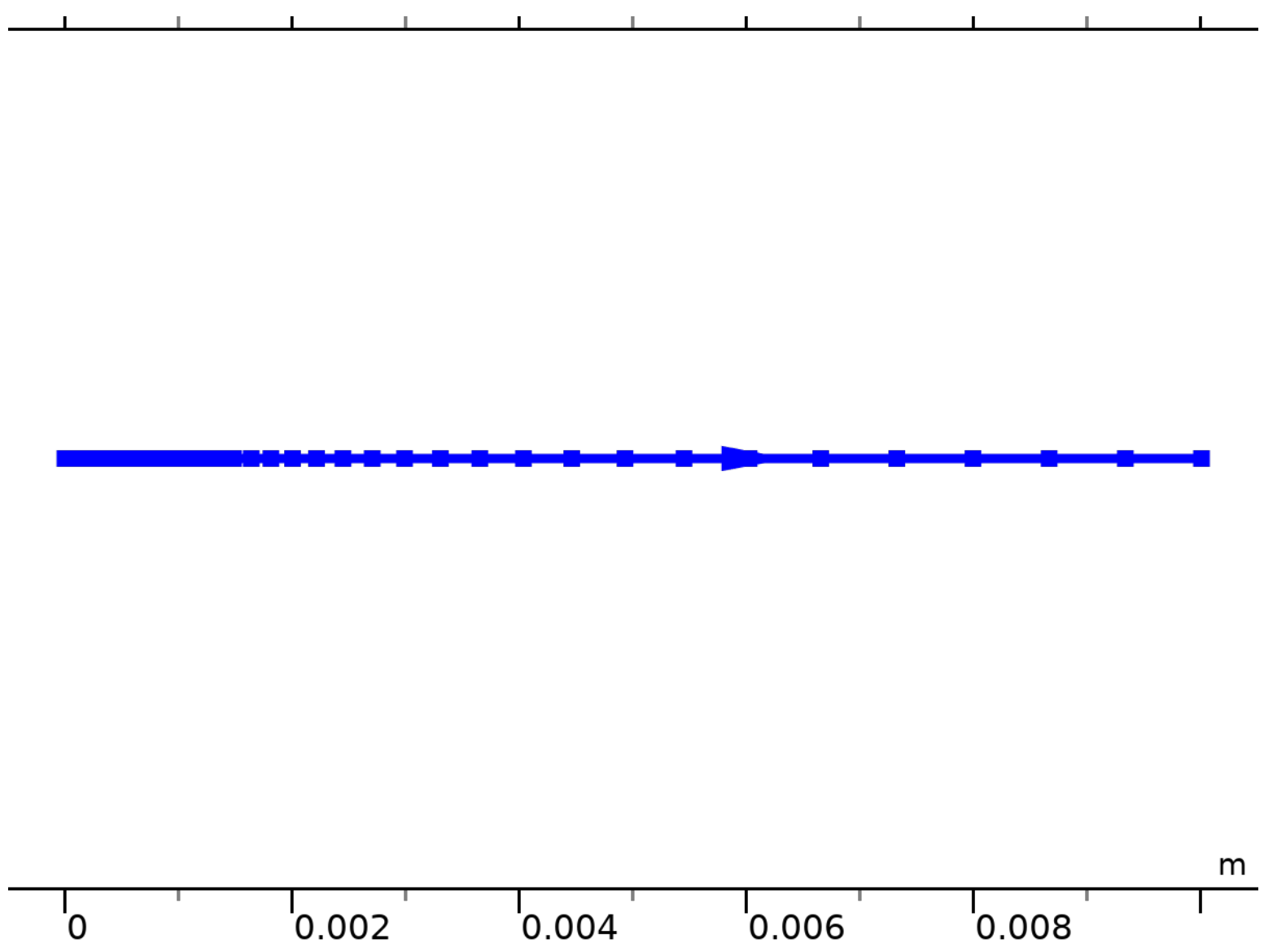

Edge 1

INFORMATION

Description	Value
Last build time	< 1 second
Built with	COMSOL 6.2.0.415 (win64), Oct 6, 2025, 3:11:46 PM

Size 1 (size1)

SELECTION

Geometric entity level	Domain
Selection	Geometry geom1: Dimension 1: Domain 1

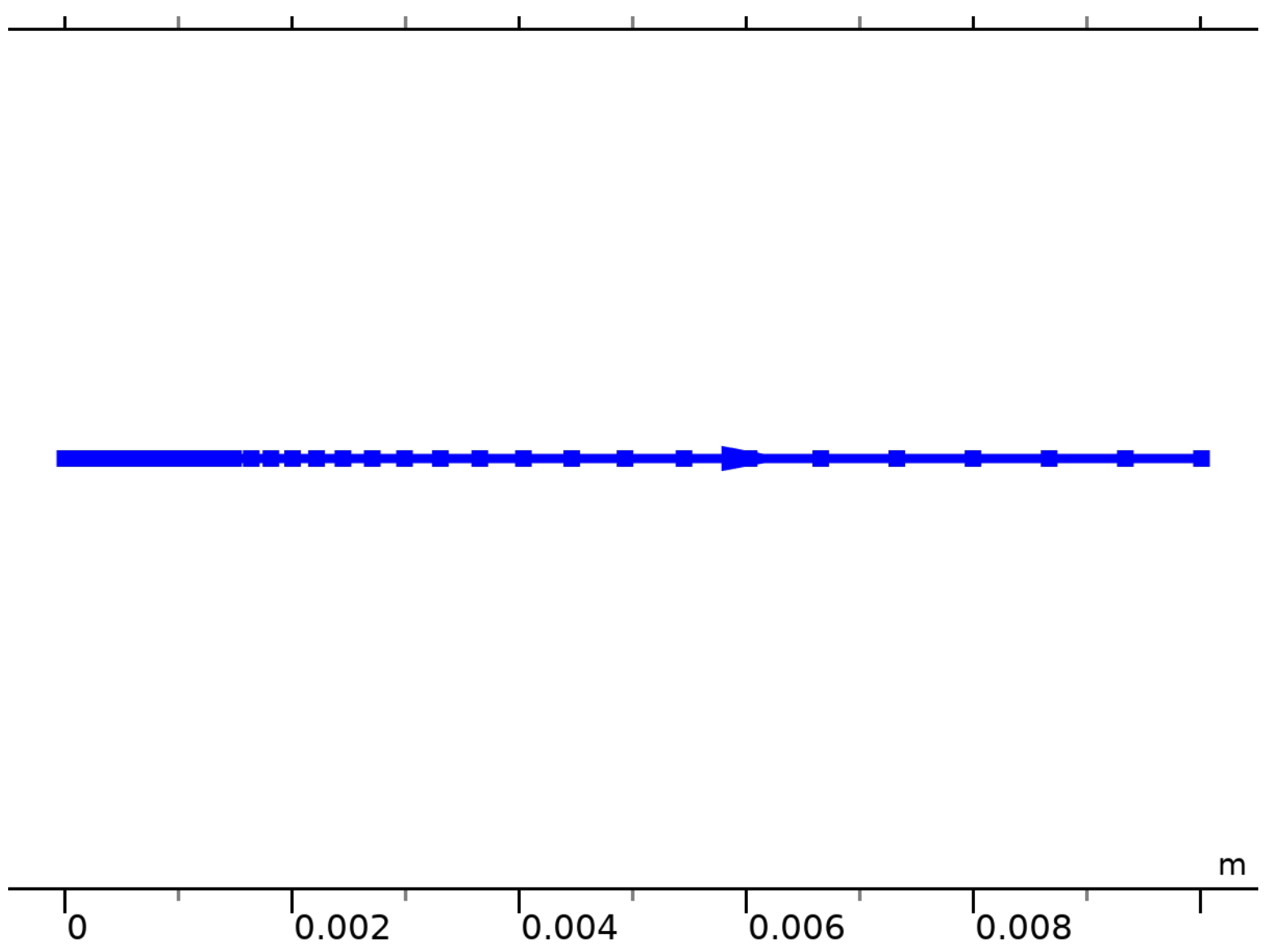

Size 1

SETTINGS

Description	Value
Maximum element size	6.7E-4
Minimum element size	3E-6
Curvature factor	0.3
Maximum element growth rate	1.3

Size 2 (size2)

SELECTION

Geometric entity level	Boundary
Selection	Geometry geom1: Dimension 0: Boundary 1

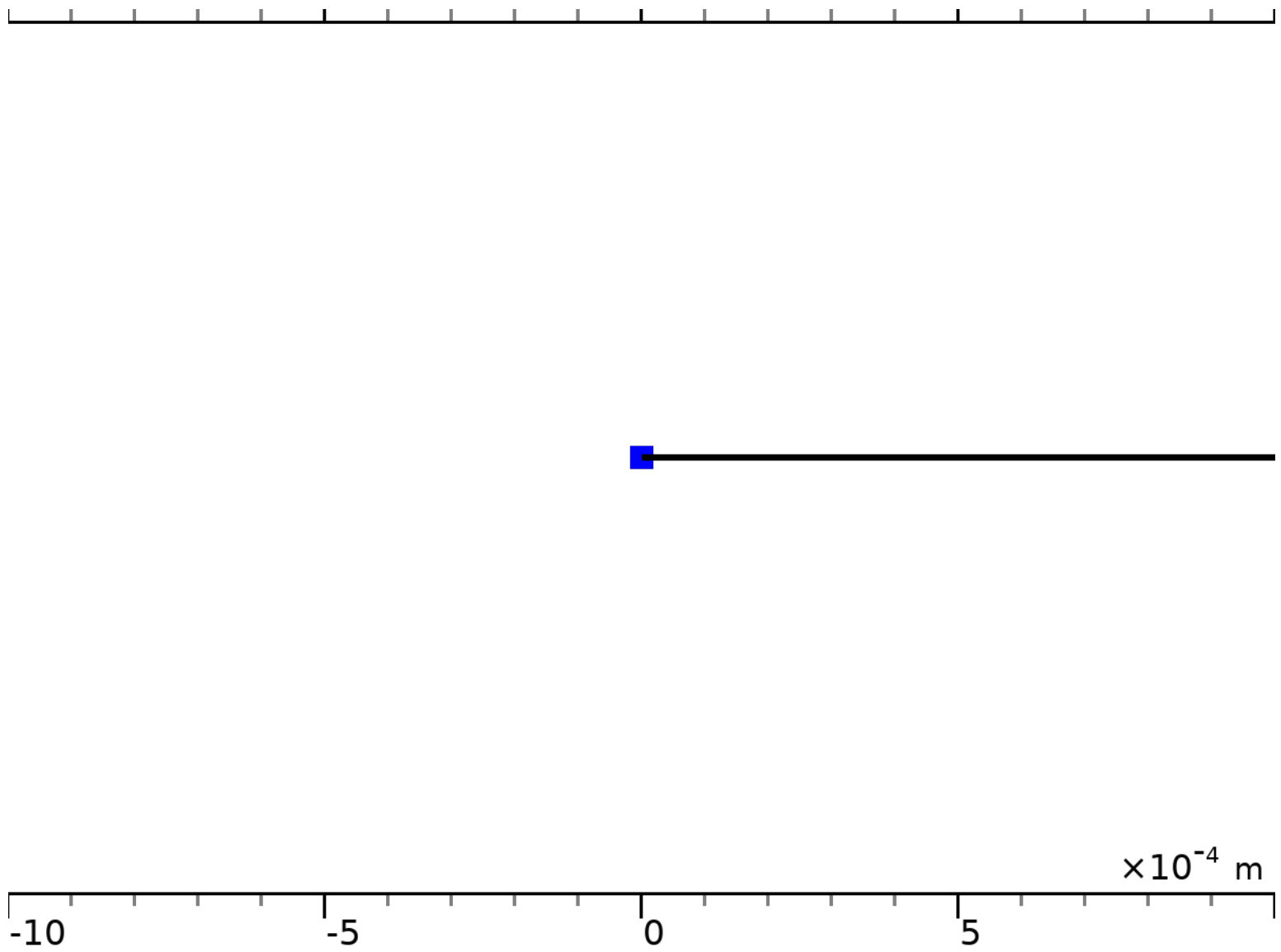

Size 2

SETTINGS

Description	Value
Maximum element size	mesh_1
Minimum element size	3E-6
Minimum element size	Off
Curvature factor	0.3
Curvature factor	Off
Resolution of narrow regions	Off
Custom element size	Custom

3 Study 1

COMPUTATION INFORMATION

Computation time	15 s

3.1 PARAMETRIC SWEEP 1

Parameter name	Parameter value list	Parameter unit
pH	range(0,1,14)	
SiteDensity	1E-13	mol/cm^2
ScanRate	10	V/s

STUDY SETTINGS

Description	Value
Sweep type	All combinations
Parameter name	{pH, SiteDensity, ScanRate}
Unit	{, mol/cm^2, V/s}

PARAMETERS

Parameter name	Parameter value list	Parameter unit
pH (Bulk pH)	range(0,1,14)	
SiteDensity (Total site density)	1E-13	mol/cm^2
ScanRate (Scan rate)	10	V/s

3.2 STATIONARY

STUDY SETTINGS

Description	Value
Include geometric nonlinearity	Off

PHYSICS AND VARIABLES SELECTION

Physics interface	Solve for	Equation form
Transport of Diluted Species (tds)	On	Automatic (Stationary)
Surface Reactions (sr)	On	Automatic (Stationary)

STORE IN OUTPUT

Interface	Output	Selection
Transport of Diluted Species (tds)	Physics controlled	
Surface Reactions (sr)	Physics controlled	

MESH SELECTION

Component	Mesh
Component 1	Mesh 1

3.3 TIME DEPENDENT

Times	Unit
range(0,t_step,SegmentPeriod*2)	s

STUDY SETTINGS

Description	Value
Include geometric nonlinearity	Off

STUDY SETTINGS

Description	Value
Output times	range(0,t_step,SegmentPeriod*2)
Tolerance	User controlled
Relative tolerance	Tol

PHYSICS AND VARIABLES SELECTION

Physics interface	Solve for	Equation form
Transport of Diluted Species (tds)	On	Automatic (Time dependent)
Surface Reactions (sr)	On	Automatic (Time dependent)

STORE IN OUTPUT

Interface	Output	Selection
Transport of Diluted Species (tds)	Physics controlled	
Surface Reactions (sr)	Physics controlled	

MESH SELECTION

Component	Mesh
Component 1	Mesh 1

3.4 SOLVER CONFIGURATIONS

3.4.1 Parametric Solutions 1

kp=1E-9 (su1)

GENERAL

Description	Value
Solution	kp=1E-9 (sol11)

kp=1E-6 (su2)

GENERAL

Description	Value
Solution	kp=1E-6 (sol12)

kp=0.001 (su3)

GENERAL

Description	Value
Solution	kp=0.001 (sol13)

kp=1 (su4)

GENERAL

Description	Value
Solution	kp=1 (sol14)

kp=1000 (su5)

GENERAL

Description	Value
Solution	kp=1000 (sol15)

kp=1E6 (su6)

GENERAL

Description	Value
Solution	kp=1E6 (sol16)

kp=1E9 (su7)

GENERAL

Description	Value
Solution	kp=1E9 (sol17)

3.4.2 Parametric Solutions 2

pH=0 (su1)

GENERAL

Description	Value
Solution	pH=0 (sol21)

pH=10 (su2)

GENERAL

Description	Value
Solution	pH=10 (sol22)

3.4.3 Solution 19

Compile Equations: Time Dependent (st1)

STUDY AND STEP

Description	Value
Use study	Study 1
Use study step	Time Dependent

Dependent Variables 2 (v2)

GENERAL

Description	Value
Defined by study step	Step 2: Time Dependent

RESIDUAL SCALING

Description	Value
Method	Manual

INITIAL VALUE CALCULATION CONSTANTS

Constant name	Initial value source
t	{range(0,t_step,SegmentPeriod*2)}[s]
timestep	0.026[s]

Surface Concentration (comp1.M) (comp1_M)

GENERAL

Description	Value
Field components	comp1.M

Surface Concentration (comp1.N) (comp1_N)

GENERAL

Description	Value
Field components	comp1.N

Surface Concentration (comp1.O) (comp1_O)

GENERAL

Description	Value
Field components	comp1.O

Surface Concentration (comp1.P) (comp1_P)

GENERAL

Description	Value
Field components	comp1.P

Surface Concentration (comp1.Q) (comp1_Q)

GENERAL

Description	Value
Field components	comp1.Q

Surface Concentration (comp1.R) (comp1_R)

GENERAL

Description	Value
Field components	comp1.R

Surface Concentration (comp1.S) (comp1_S)

GENERAL

Description	Value
Field components	comp1.S

Surface Concentration (comp1.T) (comp1_T)

GENERAL

Description	Value
Field components	comp1.T

Surface Concentration (comp1.V) (comp1_V)

GENERAL

Description	Value
Field components	comp1.V

Concentration (comp1.H) (comp1_H)

GENERAL

Description	Value
Field components	comp1.H
Internal variables	{comp1.uflux.H, comp1.dflux.H, comp1.tds.dt2Inv_H}

Concentration (comp1.OH) (comp1_OH)

GENERAL

Description	Value
Field components	comp1.OH
Internal variables	{comp1.uflux.OH, comp1.dflux.OH, comp1.tds.dt2Inv_OH}

Dependent Variables 1 (v1)

INITIAL VALUE CALCULATION CONSTANTS

Constant name	Initial value source
t	{range(0,t_step,SegmentPeriod*2)}[s]
timestep	0.026[s]

Surface Concentration (comp1.M) (comp1_M)

GENERAL

Description	Value
Field components	comp1.M

Surface Concentration (comp1.N) (comp1_N)

GENERAL

Description	Value
Field components	comp1.N

Surface Concentration (comp1.O) (comp1_O)

GENERAL

Description	Value
Field components	comp1.O

Surface Concentration (comp1.P) (comp1_P)

GENERAL

Description	Value
Field components	comp1.P

Surface Concentration (comp1.Q) (comp1_Q)

GENERAL

Description	Value
Field components	comp1.Q

Surface Concentration (comp1.R) (comp1_R)

GENERAL

Description	Value
Field components	comp1.R

Surface Concentration (comp1.S) (comp1_S)

GENERAL

Description	Value
Field components	comp1.S

Surface Concentration (comp1.T) (comp1_T)

GENERAL

Description	Value
Field components	comp1.T

Surface Concentration (comp1.V) (comp1_V)

GENERAL

Description	Value
Field components	comp1.V

Concentration (comp1.H) (comp1_H)

GENERAL

Description	Value
Field components	comp1.H
Internal variables	{comp1.uflux.H, comp1.dflux.H, comp1.tds.dt2Inv_H}

Concentration (comp1.OH) (comp1_OH)

GENERAL

Description	Value
Field components	comp1.OH
Internal variables	{comp1.uflux.OH, comp1.dflux.OH, comp1.tds.dt2Inv_OH}

Time-Dependent Solver 1 (t1)

GENERAL

Description	Value
Defined by study step	Step 2: Time Dependent
Output times	range(0,t_step,SegmentPeriod*2)
Relative tolerance	Tol

ABSOLUTE TOLERANCE

Field	Method	Tolerance method	Tolerance factor	Derivative tolerance method	Time derivative factor	Tolerance	Tolerance for time derivatives
Surface Concentration (comp1.M)	Use global	Factor	0.1	Automatic	1	0.001	0.001
Surface Concentration (comp1.N)	Use global	Factor	0.1	Automatic	1	0.001	0.001
Surface Concentration (comp1.O)	Use global	Factor	0.1	Automatic	1	0.001	0.001
Surface Concentration (comp1.P)	Use global	Factor	0.1	Automatic	1	0.001	0.001
Surface Concentration (comp1.Q)	Use global	Factor	0.1	Automatic	1	0.001	0.001
Surface Concentration (comp1.R)	Use global	Factor	0.1	Automatic	1	0.001	0.001
Surface Concentration (comp1.S)	Use global	Factor	0.1	Automatic	1	0.001	0.001
Surface Concentration (comp1.T)	Use global	Factor	0.1	Automatic	1	0.001	0.001
Surface Concentration (comp1.V)	Use global	Factor	0.1	Automatic	1	0.001	0.001
Concentration (comp1.H)	Use global	Factor	0.1	Automatic	1	0.001	0.001
Concentration	Use global	Factor	0.1	Automatic	1	0.001	0.001

Field	Method	Tolerance method	Tolerance factor	Derivative tolerance method	Time derivative factor	Tolerance	Tolerance for time derivatives
(comp1.OH)							

TIME STEPPING

Description	Value
Steps taken by solver	Intermediate
Maximum BDF order	2

Advanced (aDef)

ASSEMBLY SETTINGS

Description	Value
Reuse sparsity pattern	On

Fully Coupled 1 (fc1)

GENERAL

Description	Value
Linear solver	Direct 1

METHOD AND TERMINATION

Description	Value
Nonlinear method	Automatic (Newton)
Maximum number of iterations	8

Direct 1 (d1)

GENERAL

Description	Value
Solver	PARDISO
Pivoting perturbation	1E-13

Solution Store 1 (su1)

GENERAL

Description	Value
Solution	Solution Store 1 (sol25)

3.4.4 Parametric Solutions 3

pH=0, SiteDensity=1E-13, ScanRate=10 (su1)

GENERAL

Description	Value
Solution	pH=0, SiteDensity=1E-13, ScanRate=10 (sol26)

pH=1, SiteDensity=1E-13, ScanRate=10 (su2)

GENERAL

Description	Value
Solution	pH=1, SiteDensity=1E-13, ScanRate=10 (sol27)

pH=2, SiteDensity=1E-13, ScanRate=10 (su3)

GENERAL

Description	Value
Solution	pH=2, SiteDensity=1E-13, ScanRate=10 (sol28)

pH=3, SiteDensity=1E-13, ScanRate=10 (su4)

GENERAL

Description	Value
Solution	pH=3, SiteDensity=1E-13, ScanRate=10 (sol29)

pH=4, SiteDensity=1E-13, ScanRate=10 (su5)

GENERAL

Description	Value
Solution	pH=4, SiteDensity=1E-13, ScanRate=10 (sol30)

pH=5, SiteDensity=1E-13, ScanRate=10 (su6)

GENERAL

Description	Value
Solution	pH=5, SiteDensity=1E-13, ScanRate=10 (sol31)

pH=6, SiteDensity=1E-13, ScanRate=10 (su7)

GENERAL

Description	Value
Solution	pH=6, SiteDensity=1E-13, ScanRate=10 (sol32)

pH=7, SiteDensity=1E-13, ScanRate=10 (su8)

GENERAL

Description	Value
Solution	pH=7, SiteDensity=1E-13, ScanRate=10 (sol33)

pH=8, SiteDensity=1E-13, ScanRate=10 (su9)

GENERAL

Description	Value
Solution	pH=8, SiteDensity=1E-13, ScanRate=10 (sol34)

pH=9, SiteDensity=1E-13, ScanRate=10 (su10)

GENERAL

Description	Value
Solution	pH=9, SiteDensity=1E-13, ScanRate=10 (sol35)

pH=10, SiteDensity=1E-13, ScanRate=10 (su11)

GENERAL

Description	Value
Solution	pH=10, SiteDensity=1E-13, ScanRate=10 (sol36)

pH=11, SiteDensity=1E-13, ScanRate=10 (su12)

GENERAL

Description	Value
Solution	pH=11, SiteDensity=1E-13, ScanRate=10 (sol37)

pH=12, SiteDensity=1E-13, ScanRate=10 (su13)

GENERAL

Description	Value
Solution	pH=12, SiteDensity=1E-13, ScanRate=10 (sol38)

pH=13, SiteDensity=1E-13, ScanRate=10 (su14)

GENERAL

Description	Value
Solution	pH=13, SiteDensity=1E-13, ScanRate=10 (sol39)

pH=14, SiteDensity=1E-13, ScanRate=10 (su15)

GENERAL

Description	Value
Solution	pH=14, SiteDensity=1E-13, ScanRate=10 (sol40)

4 Results

4.1 DATASETS

4.1.1 No Solution (1)

SOLUTION

Description	Value
Component	Component 1 (comp1)

4.1.2 Study 1/Parametric Solutions 1

SOLUTION

Description	Value
Solution	Parametric Solutions 1 (sol2)
Component	Component 1 (comp1)

4.1.3 No Solution (3)

SOLUTION

Description	Value
Component	Component 1 (comp1)

4.1.4 Study 1/Parametric Solutions 2

SOLUTION

Description	Value
Solution	Parametric Solutions 2 (sol18)
Component	Component 1 (comp1)

4.1.5 Probe Solution 5

SOLUTION

Description	Value
Component	Component 1 (comp1)

4.1.6 Point Probe 1

SELECTION

Geometric entity level	Boundary
Selection	Geometry geom1: Dimension 0: Boundary 1

DATA

Description	Value
Dataset	Probe Solution 5

SETTINGS

Description	Value
Method	Integration
Integration order	4
Integration order	On

4.1.7 Study 1/Solution 19

SOLUTION

Description	Value
Solution	Solution 19 (sol19)
Component	Component 1 (comp1)

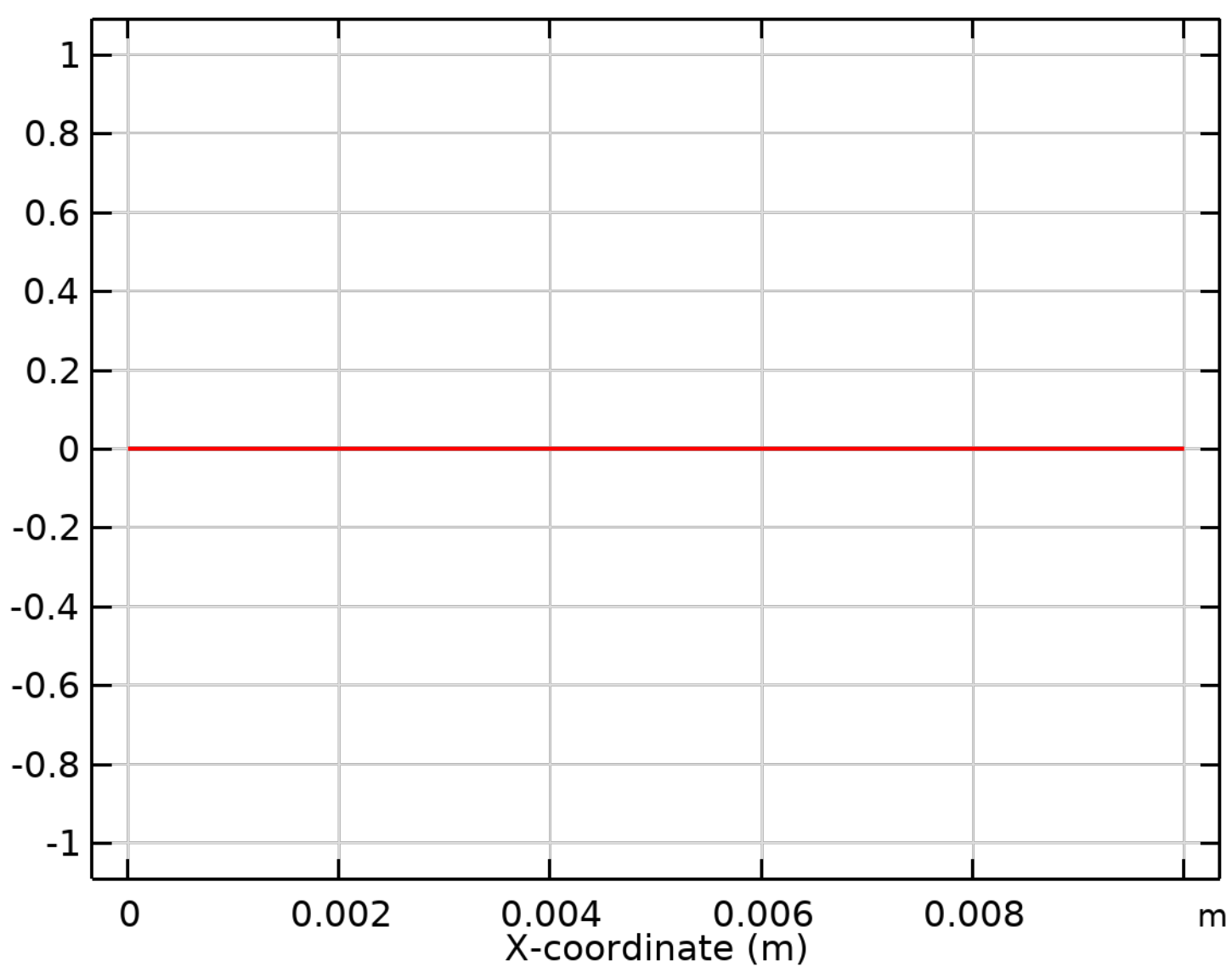

Dataset: Study 1/Solution 19

4.1.8 Study 1/Parametric Solutions 3

SOLUTION

Description	Value
Solution	Parametric Solutions 3 (sol23)
Component	Component 1 (comp1)

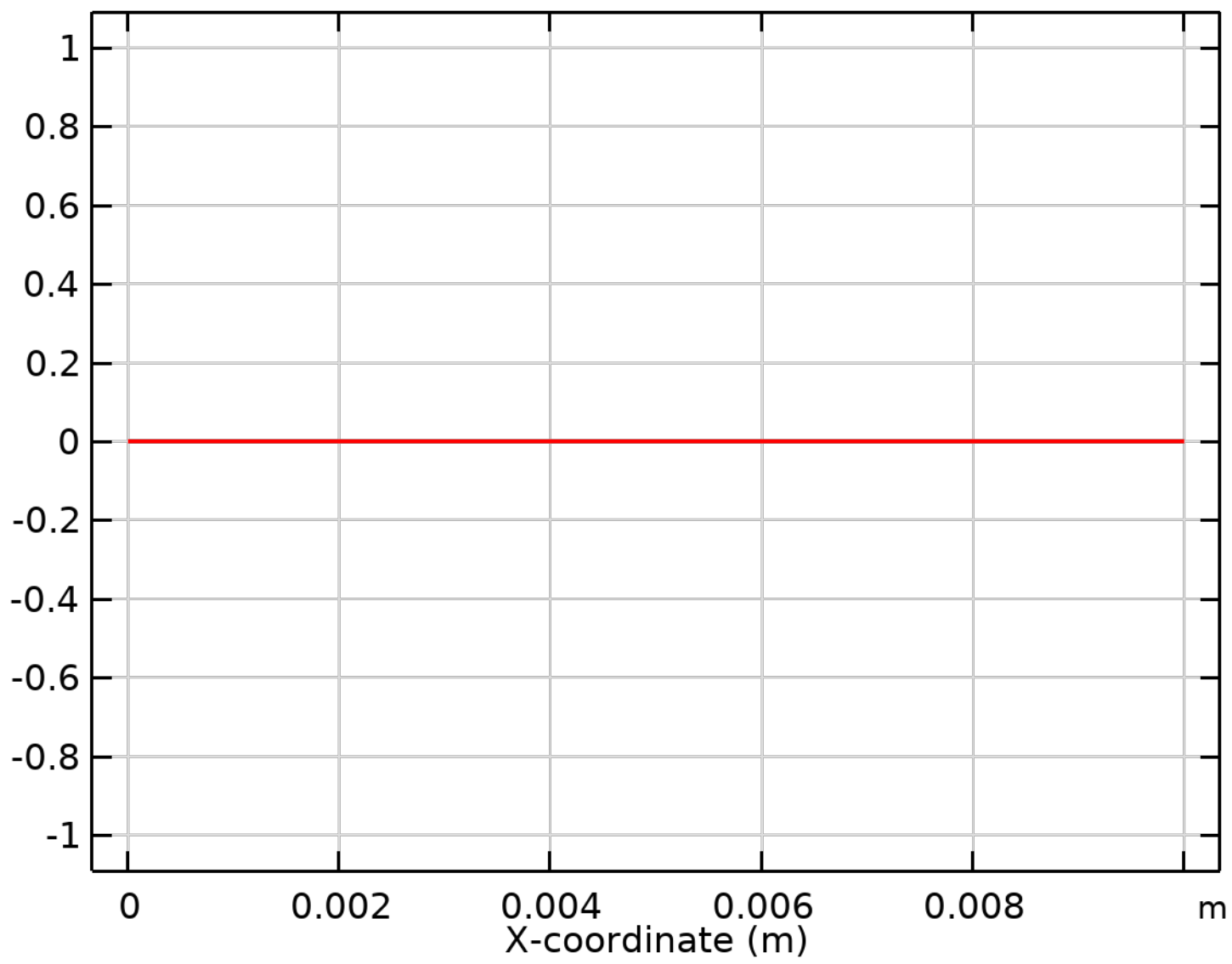

Dataset: Study 1/Parametric Solutions 3

4.1.9 Study 1/Solution Store 1

SOLUTION

Description	Value
Solution	Solution Store 1 (sol25)
Component	Component 1 (comp1)

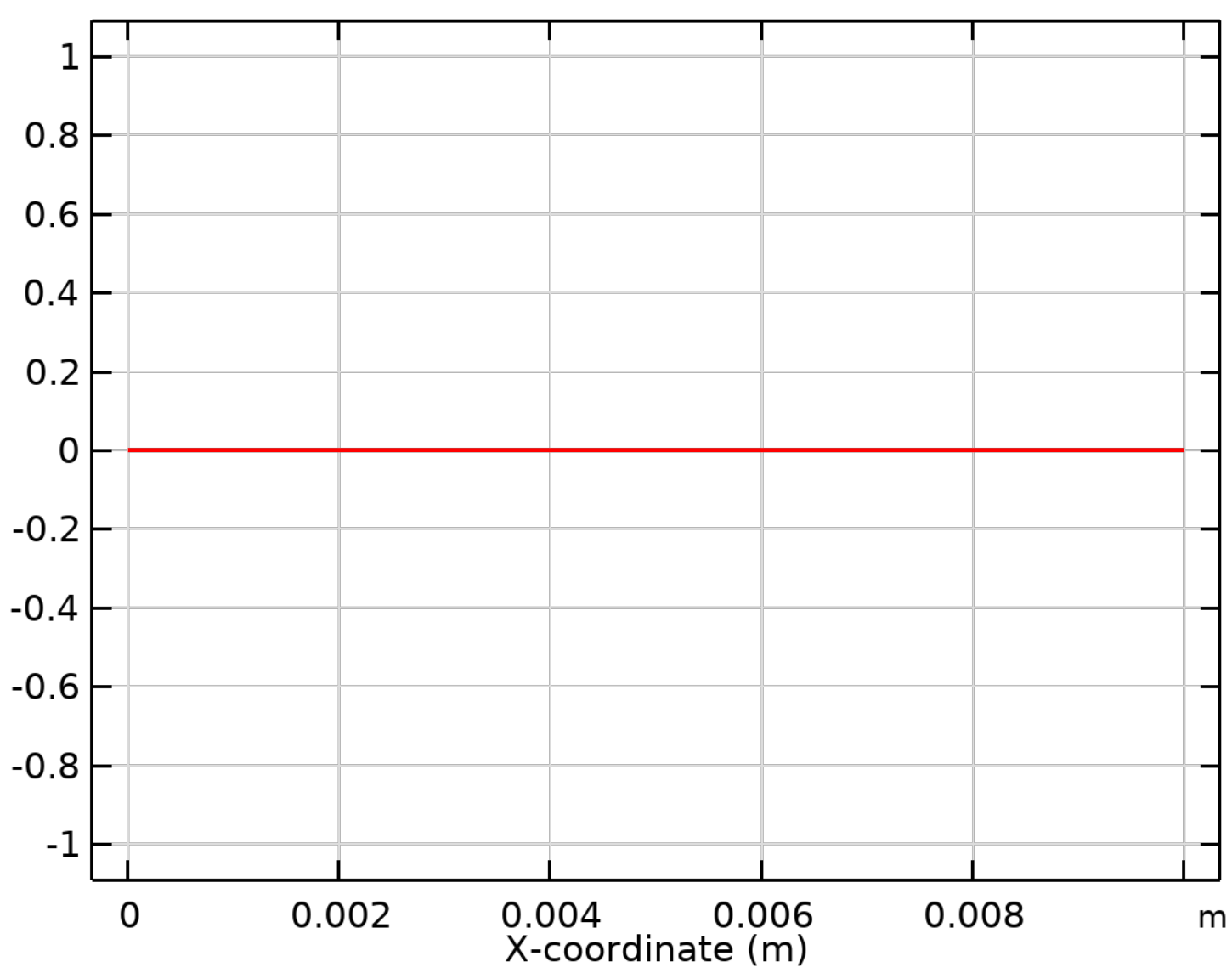

Dataset: Study 1/Solution Store 1

4.2 DERIVED VALUES

4.2.1 Point Probe 1

OUTPUT

Evaluated in	Accumulated Probe Table 2

DATA

Description	Value
Dataset	Point Probe 1

EXPRESSIONS

Expression	Unit	Description
d(S+T+V,t)*F_const - d(M+O+Q,t)*F_const	A/m^2	

4.3 TABLES

4.3.1 Probe Table 1

Time (s)	d(S+T+V,t)*F_const - d(M+O+Q,t)*F_const (A/m^2), Point Probe 1

4.4 PLOT GROUPS

4.4.1 Current Density

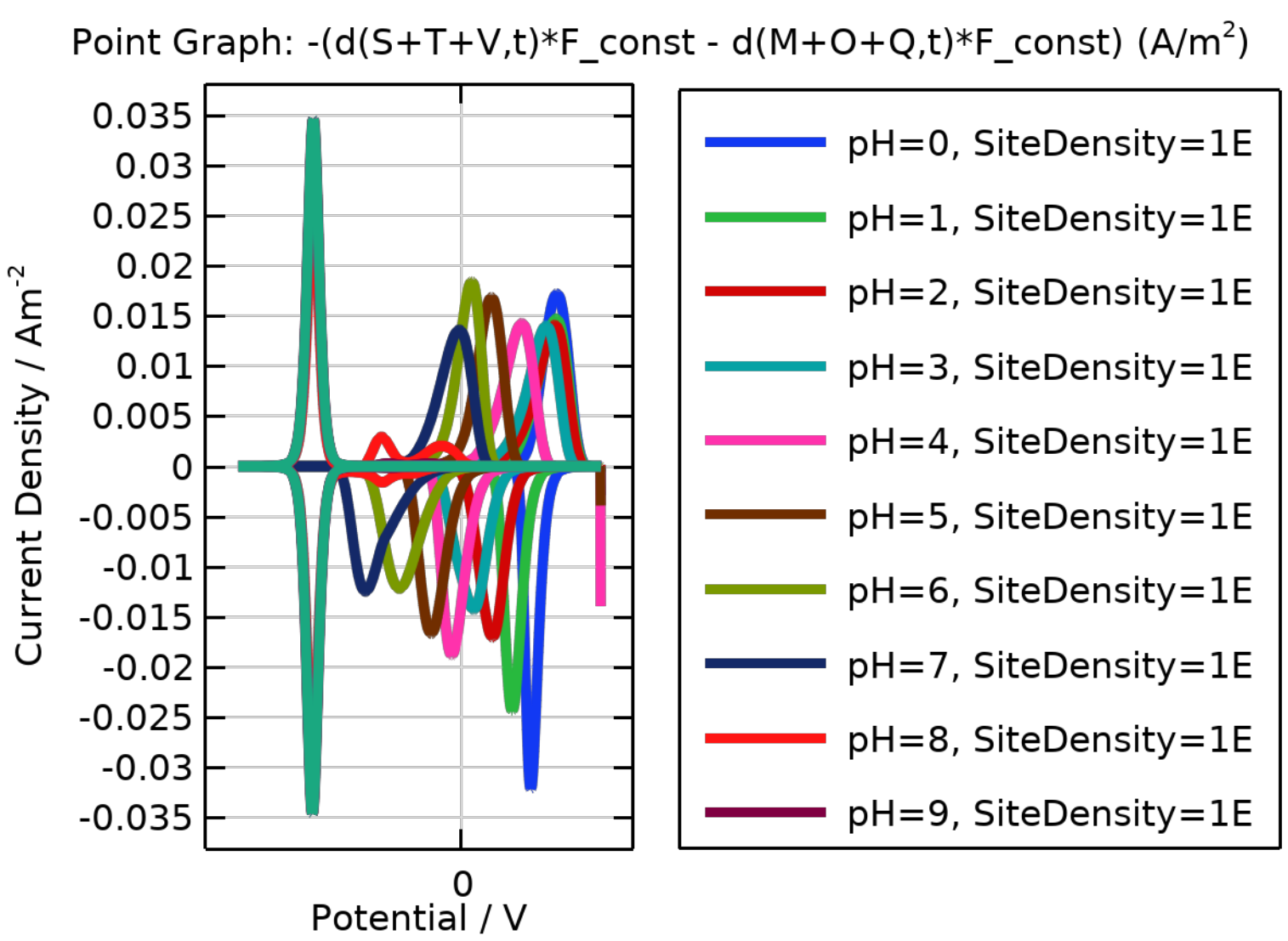

*Point Graph: -(d(S+T+V,t)*F_const - d(M+O+Q,t)*F_const) (A/m^2)*

4.4.2 Surface Species Concentration (sr)

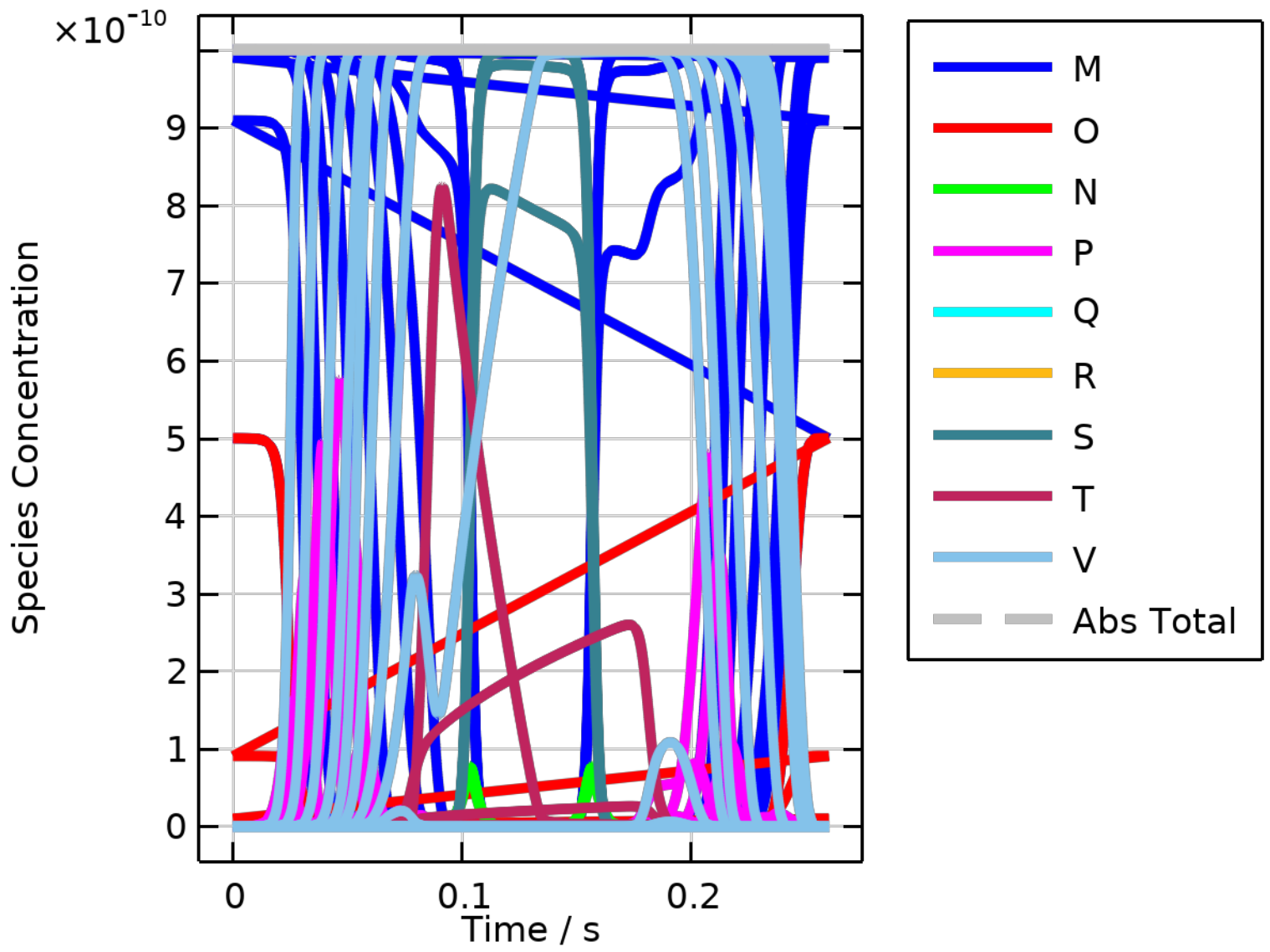

4.4.3 H+ Concentration

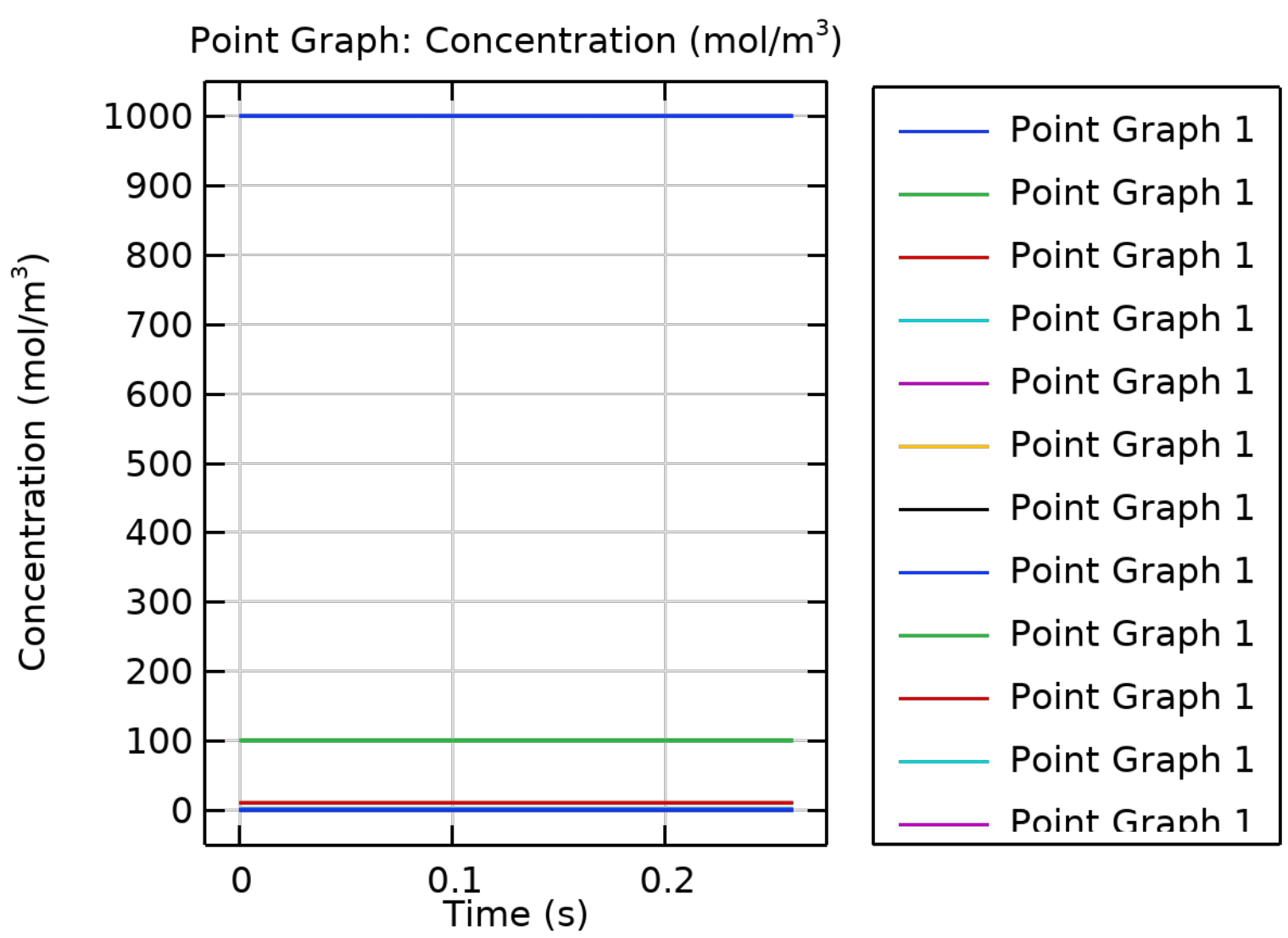

Point Graph: Concentration (mol/m³)

4.4.4 Probe Plot Group 11

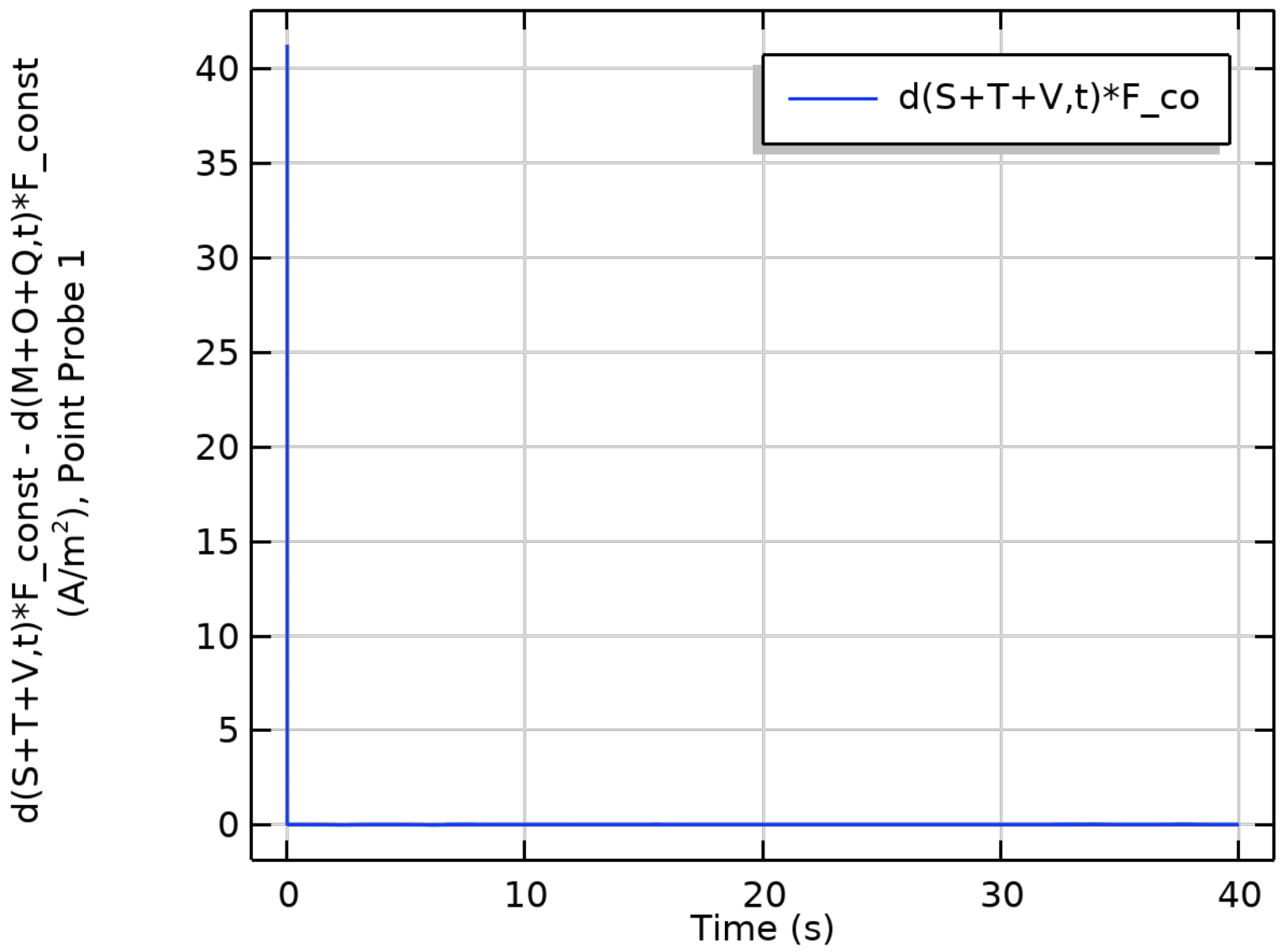

4.4.5 H+ Conc Across Domain

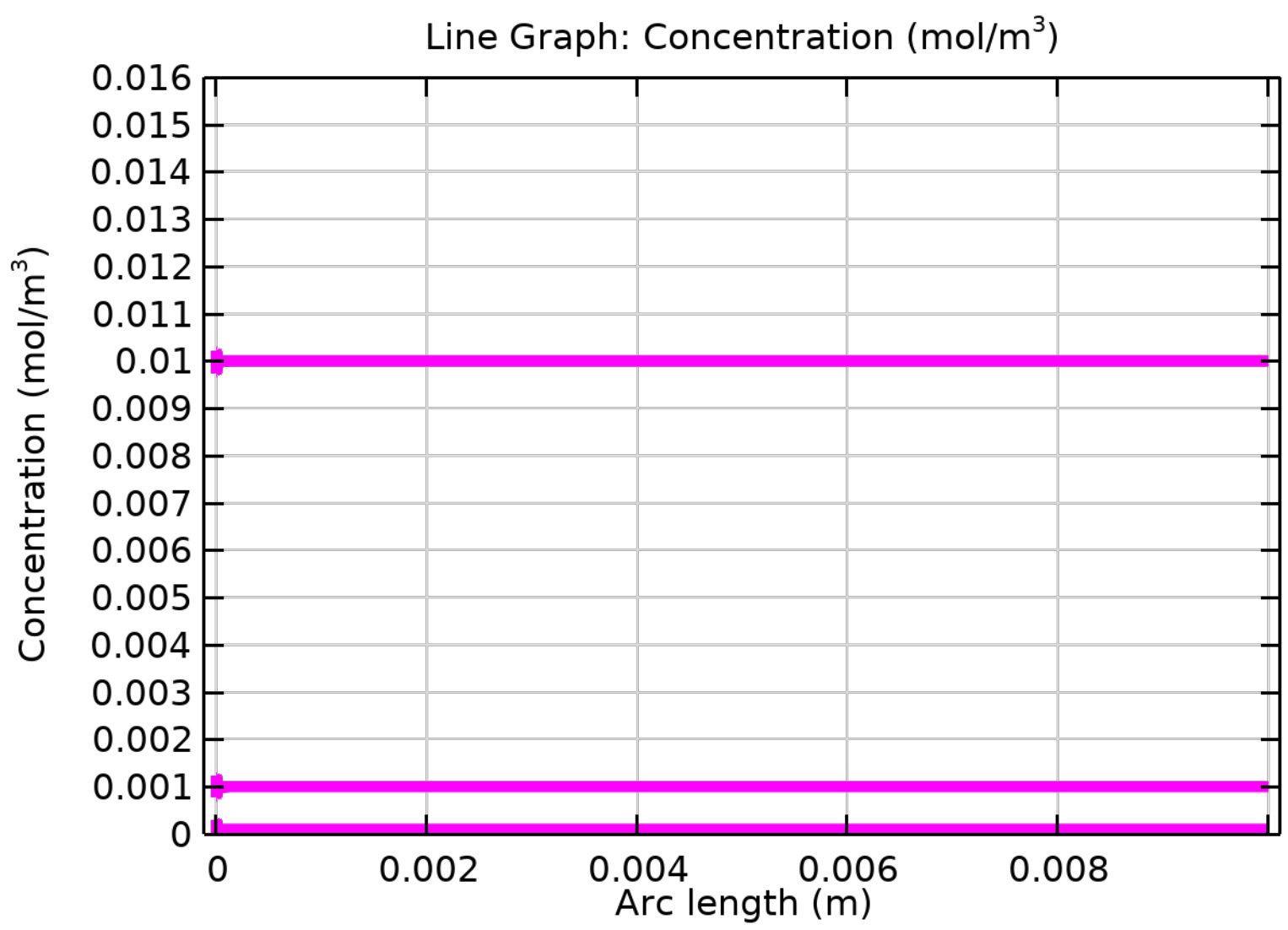

Line Graph: Concentration (mol/m^3)

4.4.6 Concentrations, All Species (tds)

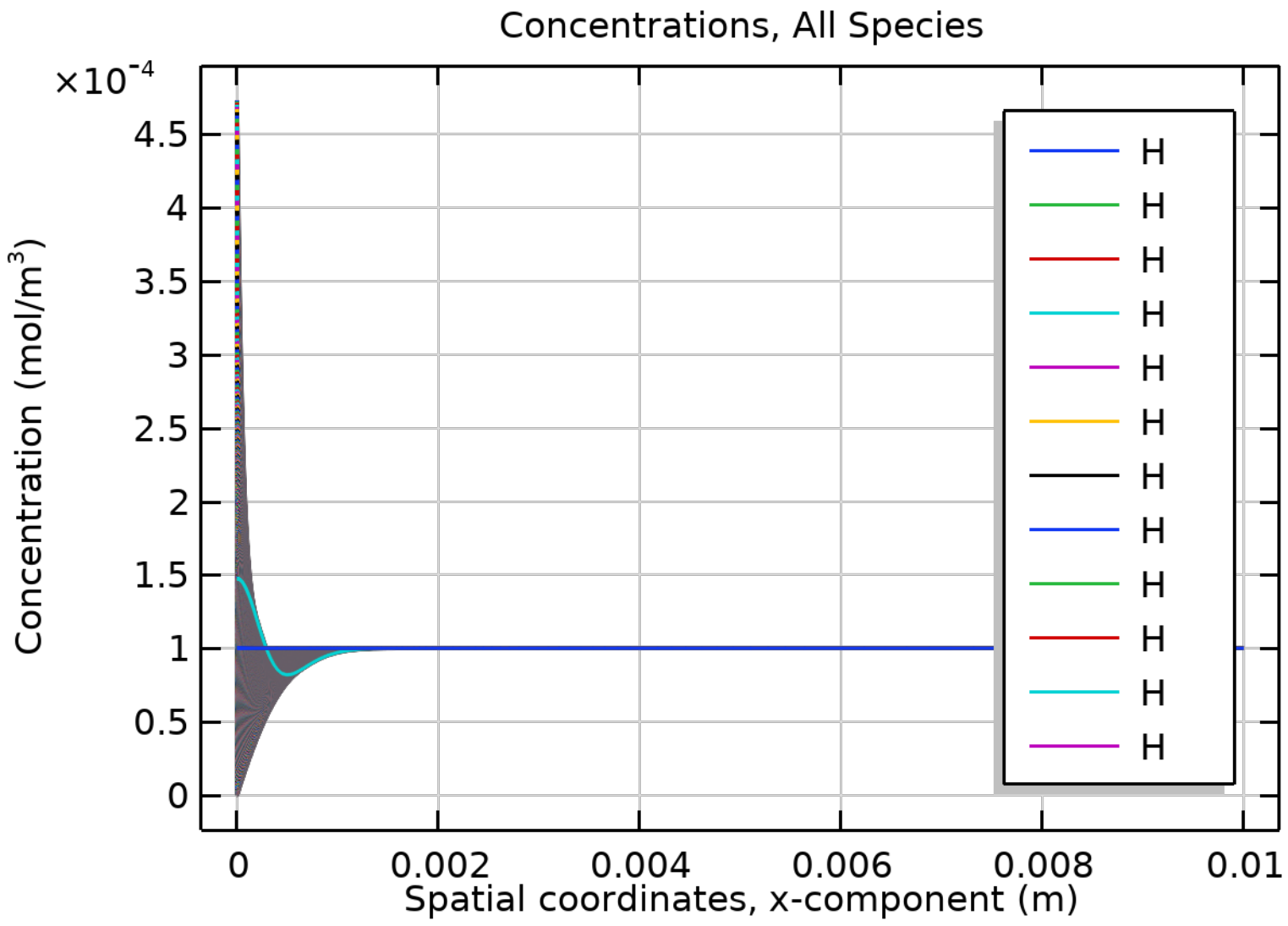

Concentrations, All Species

4.4.7 Concentration, H (tds)

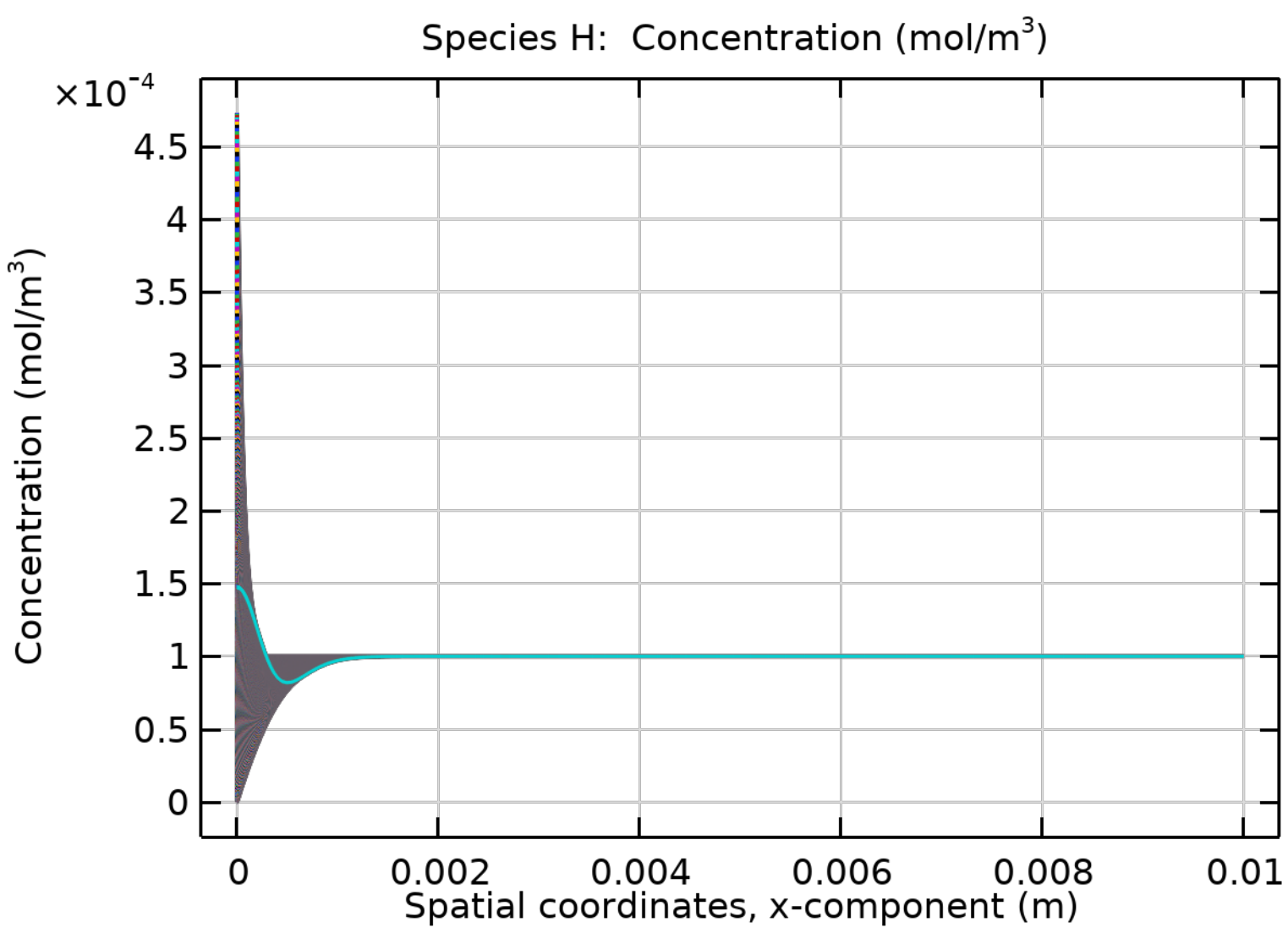

Species H: Concentration (mol/m^3)

4.4.8 Concentration, OH (tds)

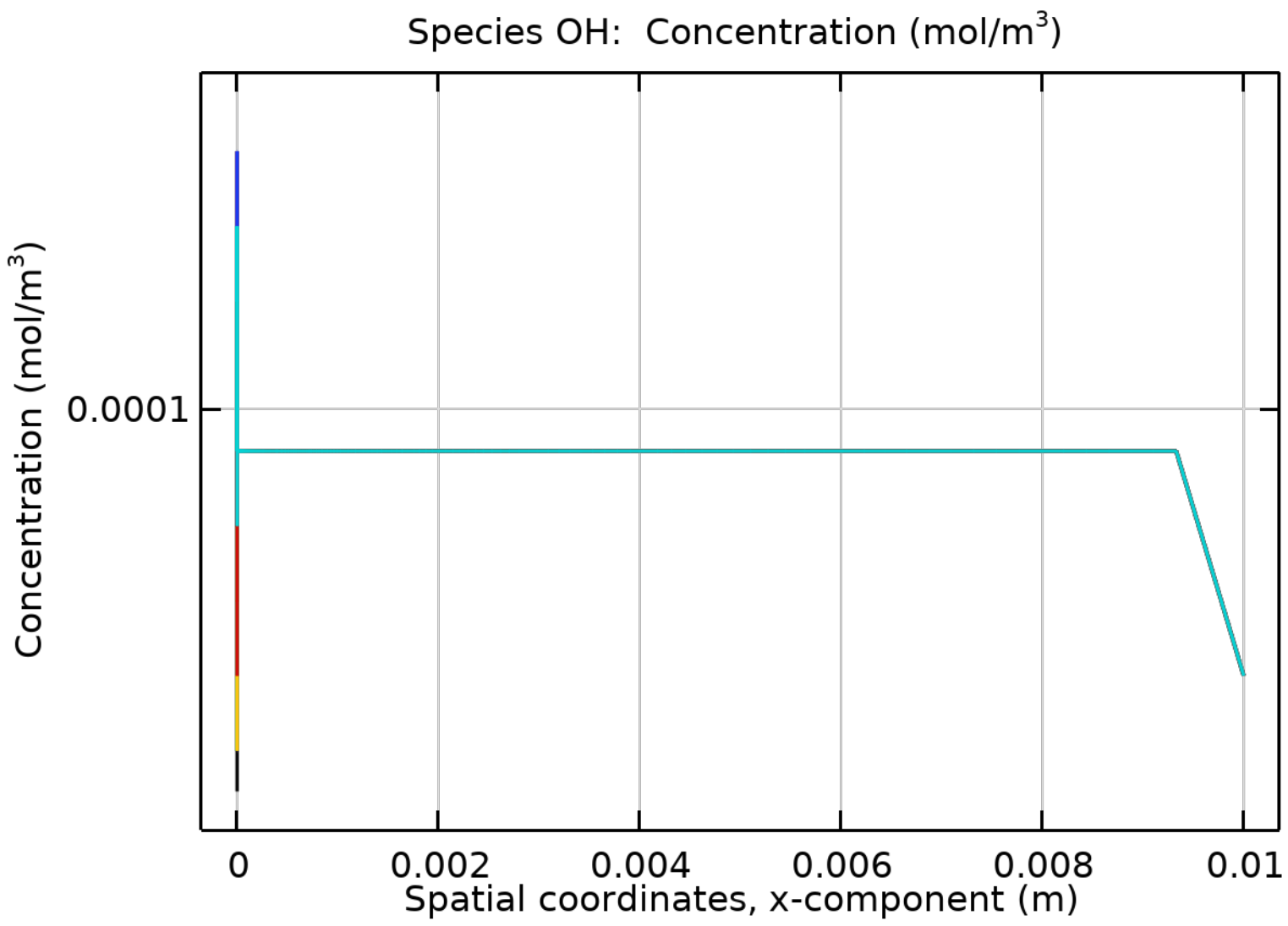

Species OH: Concentration (mol/m^3)

4.4.9 Surface Species Concentration (sr) 1

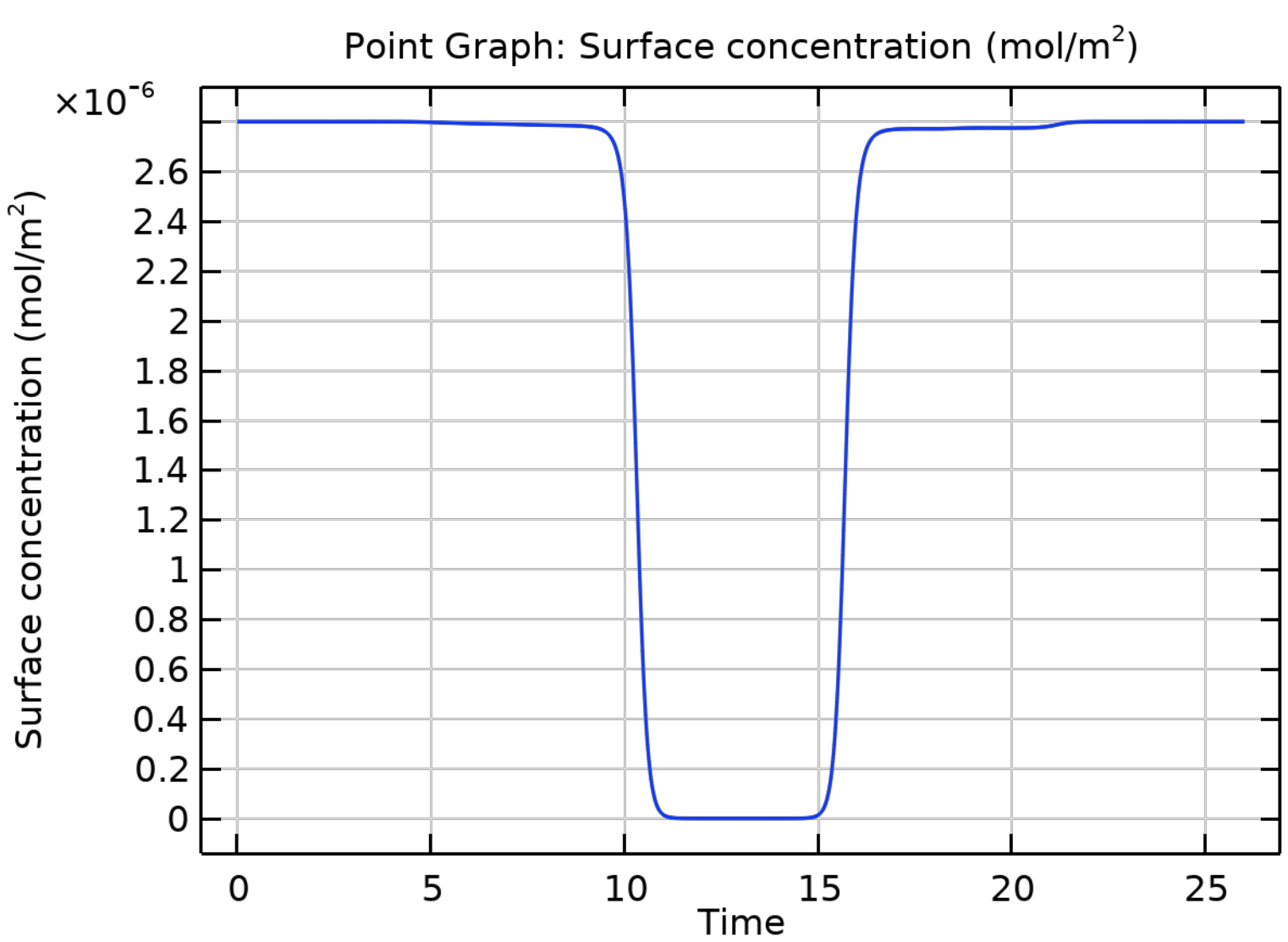

Point Graph: Surface concentration (mol/m^2)
